# Chiral bisoxazoline ligands designed to stabilize bimetallic complexes

**DOI:** 10.3762/bjoc.14.175

**Published:** 2018-08-01

**Authors:** Deepankar Das, Rudrajit Mal, Nisha Mittal, Zhengbo Zhu, Thomas J Emge, Daniel Seidel

**Affiliations:** 1 Department of Chemistry and Chemical Biology, Rutgers, The State University of New Jersey, Piscataway, NJ 08854, USA; 2 Center for Heterocyclic Compounds, Department of Chemistry, University of Florida, Gainesville, Florida 32611, USA

**Keywords:** bimetallic complexes, bisoxazolines, chiral ligands, heterocycles

## Abstract

Chiral bisoxazoline ligands containing naphthyridine, pyridazine, pyrazole, and phenol bridging units were prepared and shown to form bimetallic complexes with various metal salts. X-ray crystal structures of bis-nickel naphthyridine-bridged, bis-zinc pyridazine-bridged, and bis-nickel as well as bis-palladium pyrazole-bridged complexes were obtained.

## Introduction

Metal-centered asymmetric catalysis most commonly relies on monometallic complexes of various chiral ligands, among which chiral bisoxazolines have been highly successful in facilitating various Lewis acid-catalyzed asymmetric transformations [1–3]. In addition to monometallic catalysis, it has long been recognized that catalysts possessing two or more metal centers in close proximity can be uniquely effective in catalyzing certain types of reactions [4–6]. Serving as a main source of inspiration in the design of chiral small-molecule systems, nature utilizes a variety of bimetallic and multi-metallic protein complexes to perform a host of biological functions [7]. Urease [8], hemerythrin [9], methane monooxygenase [10], ribonucleotide reductase [11], catechol oxidase [12], and arginase [13], are prominent examples of such bimetallic enzymes.

**Figure 1:**
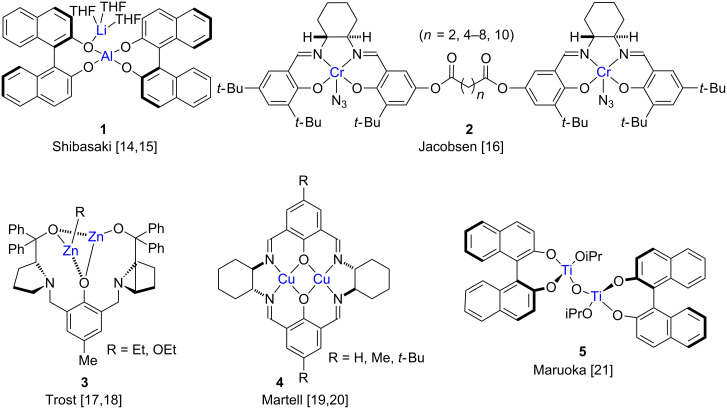
Examples of chiral bimetallic complexes utilized in asymmetric catalysis.

A range of bi- and multi-metallic complexes have been utilized in asymmetric catalysis (Figure 1) [6]. For instance, Shibasaki and co-workers introduced a number of chiral multi-metallic complexes such as the hetero-bimetallic complex **1**, in which the two different metals play distinct roles [14–15]. Jacobsen and co-workers reported dimeric salen complexes **2** which show cooperative reactivity between the two metal centers in the asymmetric ring opening of *meso*-epoxides [16]. Trost et al. disclosed the synthesis of dinuclear zinc complexes **3** and their application to enantioselective Aldol reactions [17–18] and a host of other asymmetric transformations [18]. Other notable contributions in this area were provided by the groups of Martell [19–20], Maruoka [21–22], Wuest [4,23–25], and others [26–46]. In the majority of cases where bimetallic complexes are used as the catalytically active species, the two metal centers perform different functions [47–48].

**Figure 2:**
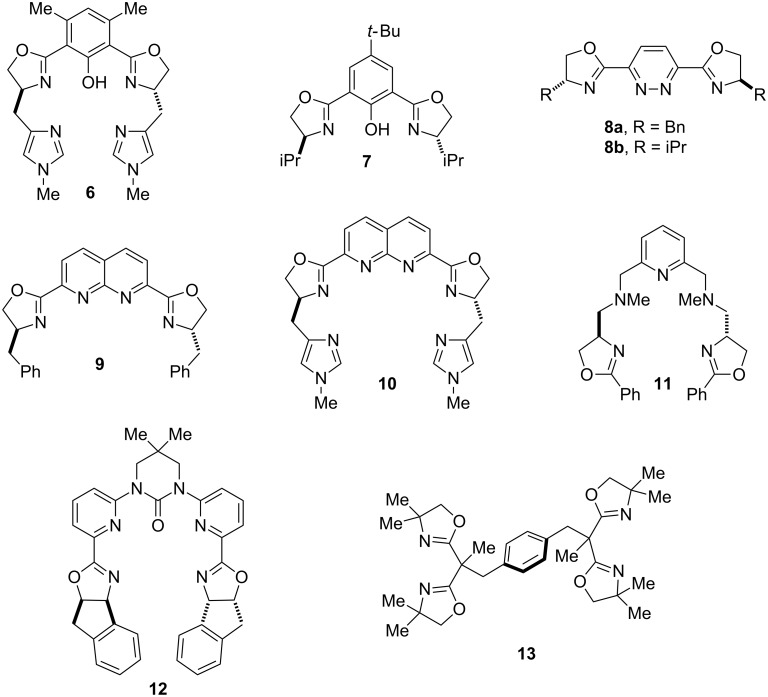
Previously reported bisoxazoline ligands capable of stabilizing bimetallic complexes.

Efforts toward synthesizing bisoxazoline ligands capable of concurrently binding to two metal centers have been rather limited. Previous work by Pfaltz, Fahrni, Tsukada, Bellemin-Laponnaz and our group resulted in the synthesis of bisoxazoline containing ligands **6**–**13** that can simultaneously bind to two metals (Figure 2). For instance, treatment of **6** with two equivalents of copper perchlorate hexahydrate led to the formation of a bis-copper complex [49–50]. The two Cu(ΙΙ) ions, which have a Cu∙∙∙Cu distance of 2.947 Å, are coordinated to the pentadentate bisoxazoline-imidazole moiety and are bridged by the central phenoxy group in addition to a hydroxide ligand. Ligand **7** forms complex binuclear complexes with ZnCl_2_ and NiCl_2_ involving two metal centers and three ligand units. The 2:3 ZnCl_2_/**7** complex crystal structure exhibits a Zn∙∙∙Zn distance of 3.056 Å. Compound **8a** forms 2:2 complexes with ZnCl_2_ and NiCl_2_, whereas **8b** was reported to form a bis-palladium complex with one equivalent of [(η^3^-C_3_H_3_)PdCl]_2_ [51]. Little is known about the complexing abilities of compounds **9** and **11** [49,52]. The naphthyridine-based ligand **10** forms a dinuclear complex with nickel(ΙΙ) acetate (Ni∙∙∙Ni distance = 3.132 Å). Compound **12**, possessing an urea backbone, forms a bis-copper complex with two equivalents of CuCl_2_ (Cu∙∙∙Cu distance = 4.291 Å) [53]. Tetraoxazoline ligand **13** undergoes formation of dinuclear complexes upon treatment with two equivalents of either ZnCl_2_, Ni(PPh_3_)_2_Br_2_, or Cu(OAc)_2_ (metal distances: Zn∙∙∙Zn = 8.963 Å; Ni∙∙∙Ni = 11.341 Å; Cu∙∙∙Cu = 9.432 Å) [54].

## Results and Discussion

A number of bisoxazoline ligands with different bridging units were designed. We rationalized that the presence of three binding sites per metal center would be ideal in order to achieve the desired 1:2 ligand to metal ratio, and to prevent the potential formation of 2:2 or other higher order complexes. Variation of the bridging moiety should allow for modulation of the distance between the metal centers. An important criterion for selecting bridging units was their known ability to engage in metal-binding, along with being readily available. This led to the selection of naphthyridine, pyridazine, pyrazole, and phenol building blocks. We opted to connect these linkers to oxazolines via amide bonds. The reasoning for this was twofold. Firstly, this should provide ligands with significantly improved stabilities over for instance imine linkers. In addition, each amide moiety, upon deprotonation (a requirement for complex formation), would provide a formal negative charge on the ligand, thus resulting in increased complex stability while reducing the number of spectator anions associated with the two metals. Different combinations of five- and six-membered chelate rings were considered, as those allow for further modulation of metal–metal distances. Convenient synthetic sequences were developed for six different ligands.

**Scheme 1:**
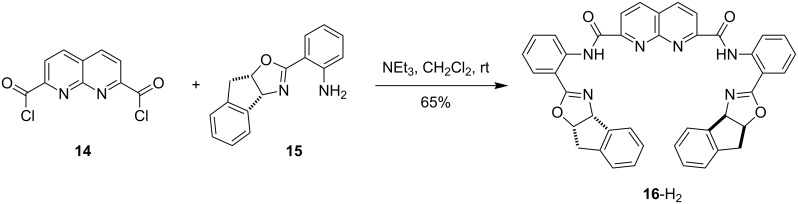
Synthesis of naphthyridine–bisoxazoline ligand **16**-H_2_.

**Naphthyridine-bridged bisoxazoline ligands.** The synthesis of naphthyridine bridged bisoxazoline ligand **16**-H_2_ is outlined in Scheme 1. 1,8-Naphthyridine-2,7-diacyl chloride **14**, obtained via a known procedure from the corresponding diacid [49], was allowed to react with aminoindanol-derived aminophenyloxazoline **15** [55] to provide bisoxazoline ligand **16**-H_2_ in 65% yield. Upon deprotonation, **16**-H_2_ provides a dianionic ligand with three nitrogen donor atoms per metal center. Ligand **16**-H_2_ was found to undergo complex formation with various copper, zinc, palladium and nickel salts.

**Figure 3:**
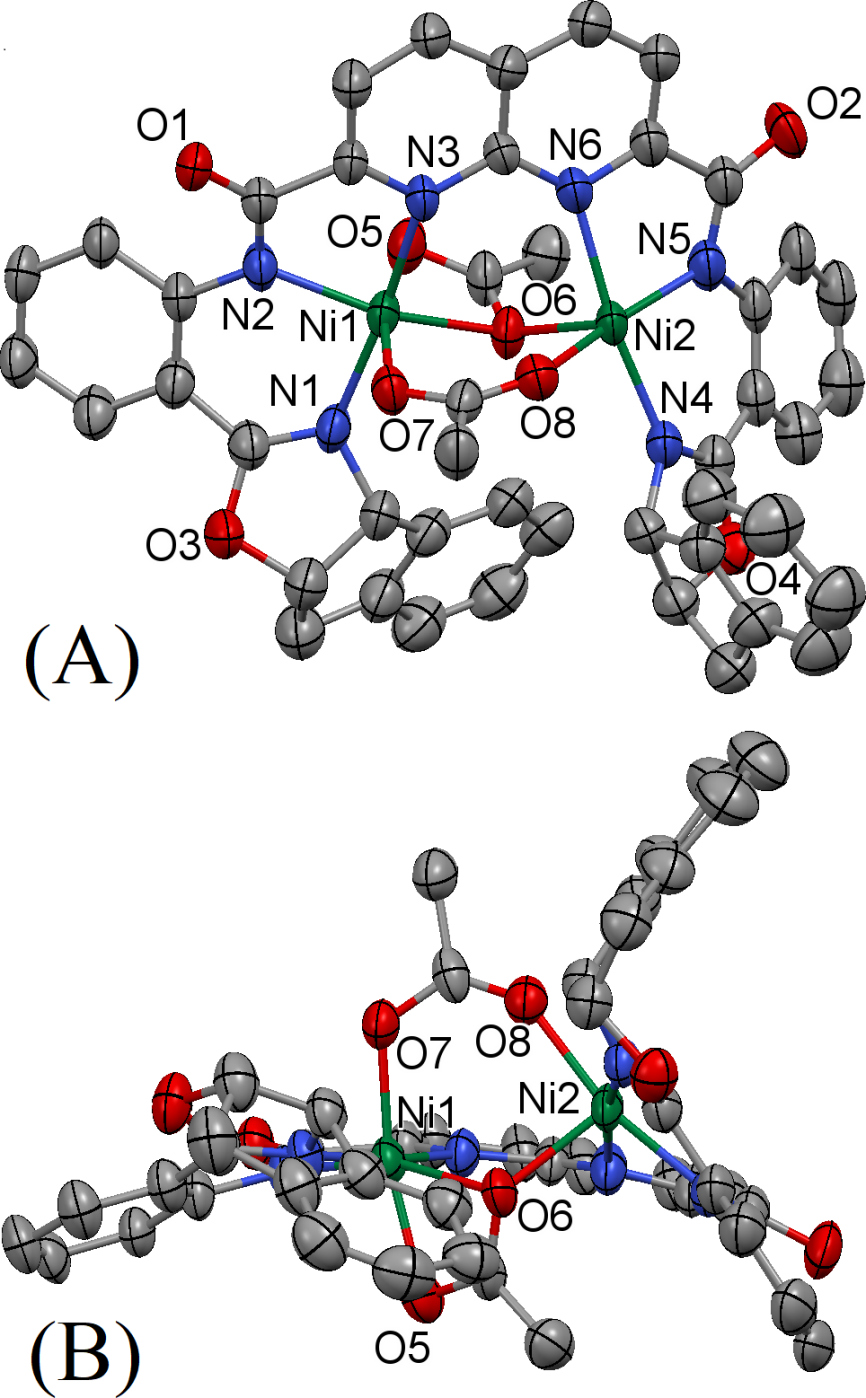
Thermal ellipsoid plot (50% probability) of the molecular structure of **16**·Ni_2_(OAc)_2_. Hydrogen and solvate atoms have been omitted for clarity: (A) view normal to the mean molecular plane; (B) side view nearly perpendicular to (A) to allow better view of the region of open access to the metal site. The complex **16**·Ni_2_(OAc)_2_ crystallizes in the orthorhombic space group *P*2_1_2_1_2_1_ with *a* = 14.1829(5) Å, *b* = 14.8645(6) Å, *c* = 25.2342(10) Å, α = 90°, β = 90°, γ = 90°, *V* = 5319.9(4) Å^3^, *Z* = 4, *D_c_* = 1.450 mg m^−3^ and μ(Mo Kα) = 1.017 mm^−1^.

Figure 3 shows the X-ray crystal structure of **16**·Ni_2_(OAc)_2_, obtained from ligand **16**-H_2_ and two equivalents of nickel(ΙΙ) acetate. The asymmetric unit of the crystal **16**·Ni_2_(OAc)_2_ contains two nickel(ΙΙ) centers held in close proximity by three donor nitrogen atoms per metal center and two differently bridged acetate ions inside the coordination sphere. The nitrogen atoms on the naphthyridine and amide moieties bind to the nickel(ΙΙ) center to form a five-membered metallacycle, subtending N(2)–Ni(1)–N(3) and N(5)–Ni(2)–N(6) angles of 81.53° and 80.34°, respectively. Additionally, the nitrogen atoms on the oxazoline and amide moieties form six-membered rings with the nickel(ΙΙ) center with N(1)–Ni(1)–N(2) and N(4)–Ni(2)–N(5) angles of 93.03° and 90.24°, respectively. All of the Ni–N distances are between 2.003 and 2.129 Å, typical for complexes of this type [56–57]. Interestingly, while one of the two acetate ions bridge the two nickel(ΙΙ) centers by binding through the two oxygens, the second acetate unit has a somewhat different binding pattern: one oxygen binds to Ni(1) and the other oxygen acts as the bridge between Ni(1) and Ni(2). The Ni(1)∙∙∙Ni(2) distance is 3.448 Å, which is slightly longer than the corresponding Ni∙∙∙Ni distance in the structurally related **10**. The sixth coordination of Ni(2) is fulfilled by the amide oxygen of a second molecule of the complex (not shown in the figure for clarity). Apparently, the coordination environment involving three O-atoms from acetate groups (the Ni(1) situation) for one of the two metals is preferred in **16**·Ni_2_(OAc)_2_, presumably for steric reasons, as crystal packing motivations would likely favor two situations as found for Ni(2), and possibly favoring formation of the 1D polymeric chain found here extending along the crystal b-axis. When this interaction is taken into account, both the nickel(ΙΙ) centers are in distorted octahedral environment. Overall, the ligand backbone of complex **16**·Ni_2_(OAc)_2_ shows a helical arrangement. This helicity is facilitated by the innate stereogenic centers of the oxazoline moieties which is further extended by the flexibility afforded by the amide connections.

**Scheme 2:**
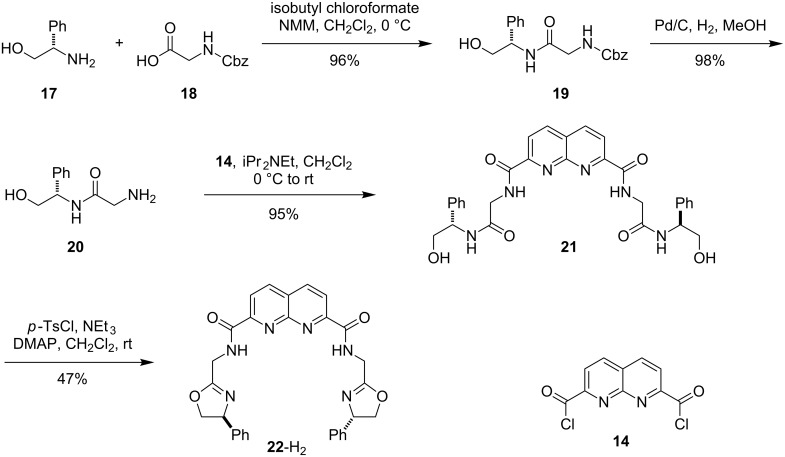
Synthesis of naphthyridine–bisoxazoline ligand **22**-H_2_.

Naphthyridine ligand **22**-H_2_ was designed as an analogue of ligand **16**-H_2_ that possesses a shortened amide bridge to allow for the formation of bimetallic complexes with all five-membered chelate rings. Ligand **22**-H_2_ was obtained starting from glycinol **17** and *N*-carboxybenzyl glycine (**20**) via the sequence of steps outlined in Scheme 2. While X-ray quality crystals have not yet been obtained, preliminary experiments have shown that ligand **22**-H_2_ undergoes complex formation with various nickel and palladium salts [58].

**Scheme 3:**
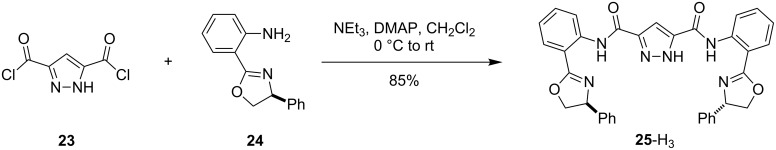
Synthesis of pyrazole–bisoxazoline ligand **25**-H_3_.

**Pyrazole-bridged bisoxazoline ligands.** The synthesis of an analog of bisoxazoline ligand **16**-H_2_ in which the naphthyridine bridge is replaced with a pyrazole linker is shown in Scheme 3. In addition to the change in geometry, compound **25**-H_3_ is a potentially trianionic as opposed to a dianionic ligand. A reaction of pyrazole-3,5-diacyl chloride **23**, obtained by treatment of pyrazol-3,5-dicarboxylic acid with thionyl chloride, with aminophenyloxazoline **24** [59] provided ligand **25**-H_3_ in 85% yield.

**Figure 4:**
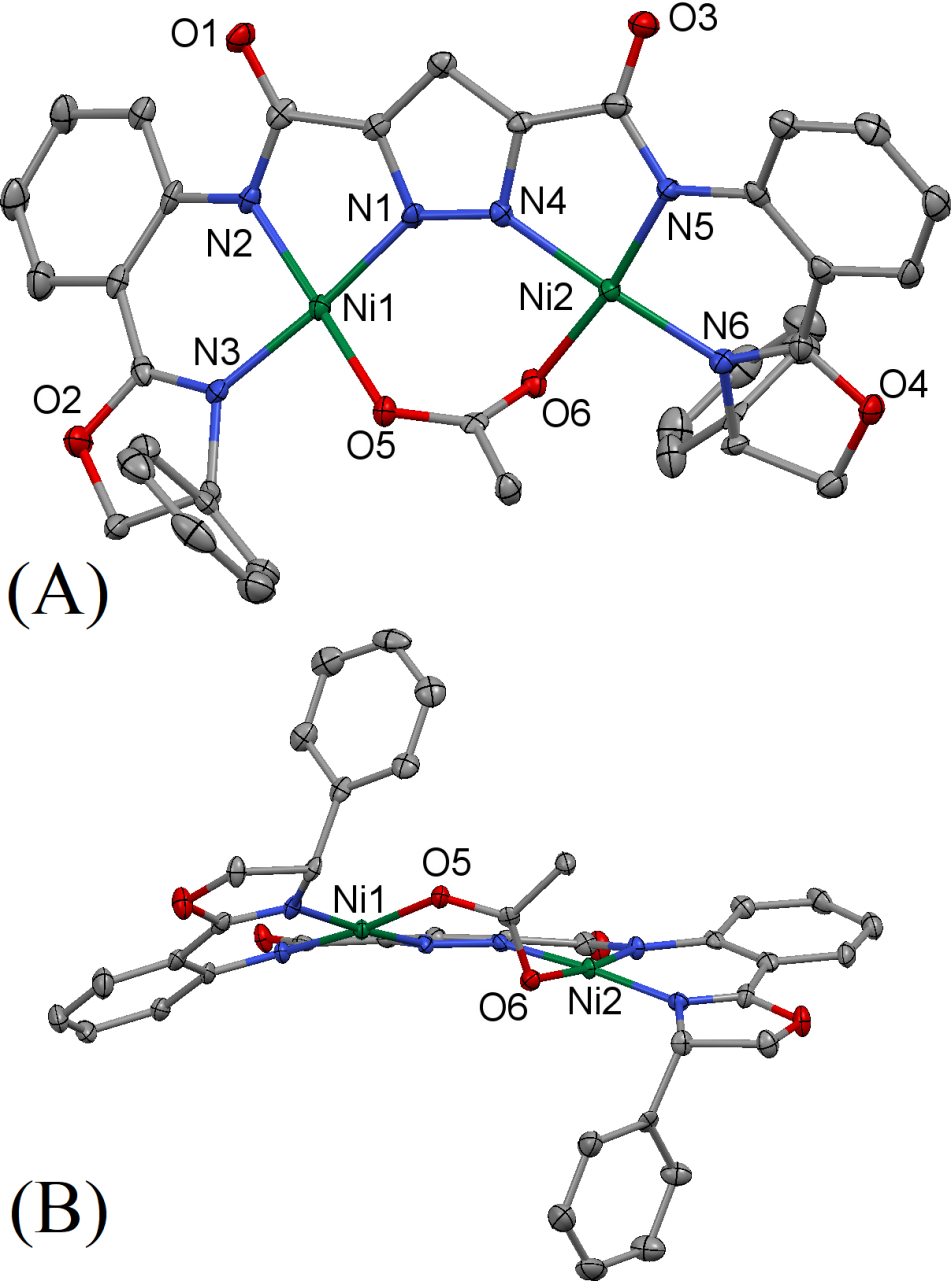
Thermal ellipsoid plot (50% probability) of the molecular structure of **25**·Ni_2_(OAc). Hydrogen and solvate atoms have been omitted for clarity: (A) view normal to the mean molecular plane; (B) side view nearly perpendicular to (A) to allow better view of the region of open access to the metal site. The complex **25**·Ni_2_(OAc) crystallizes in the monoclinic space group *P*2_1_ with *a* = 9.0473(9) Å, *b* = 17.5433(17) Å, *c* = 11.7671(11) Å, α = 90°, β = 109.643(2)°, γ = 90°, *V* = 1759.0(3) Å^3^, *Z* = 2, *D_c_* = 1.614 mg m^−3^ and μ(Mo Kα) = 1.281 mm^−1^.

Treatment of **25**-H_3_ with nickel(II) acetate provided the binuclear complex **25**·Ni_2_(OAc), the X-ray structure of which is shown in Figure 4. Each unit of the complex is comprised of two nickel(II) centers, each bound by three nitrogen atoms from the ligand skeleton and bridged by an acetate anion. The nitrogen atoms from pyrazole and amide moieties coordinate to the two nickel centers to form five-membered metallacycles, subtending N(1)–Ni(1)–N(2) and N(4)–Ni(2)–N(5) angles of 84.0° and 85.0°, respectively. Similarly, the nitrogen atoms on amide and oxazoline moieties form six-membered metallacyles with the two metal centers, with N(2)–Ni(1)–N(3) and N(5)–Ni(2)–N(6) angles of 92.63° and 94.33°, respectively. All the Ni–N distances are between 1.855 and 1.903 Å, typical for Ni–N bonds having this coordination geometry. The two nickel centers are bridged by an acetate ion and the pyrazole segment of the ligand, with a distorted square planar geometry observed for both metal centers. The resulting Ni(1)∙∙∙Ni(2) distance is 4.176 Å.

**Scheme 4:**
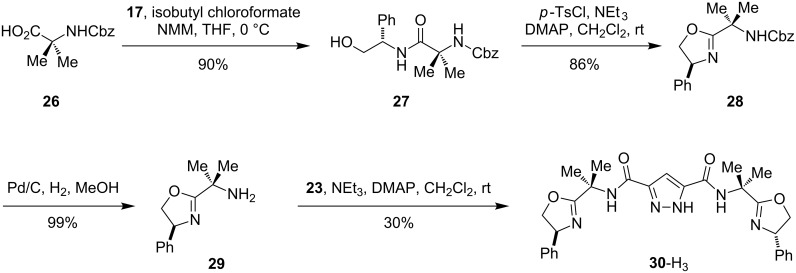
Synthesis of pyrazole–bisoxazoline ligand **30**-H_3_.

The synthesis of a pyrazole containing ligand potentially capable of forming bimetallic complexes with all five-membered chelate rings is shown in Scheme 4. Trianionic ligand **30**-H_3_ was prepared starting from the *N*-carboxybenzyl amino acid **26**. Coupling of **26** with phenylglycinol (**17**) resulted in the formation of the amide **27**. Subsequent treatment with *p*-toluenesulfonyl chloride and triethylamine in the presence of a catalytic amount of DMAP provided oxazoline **28**. Following deprotection, the resulting amine **29** was allowed to react with half an equivalent of the pyrazole diacyl chloride **23** to yield the pyrazole-bridged bisoxazoline ligand **30**-H_3_.

**Figure 5:**
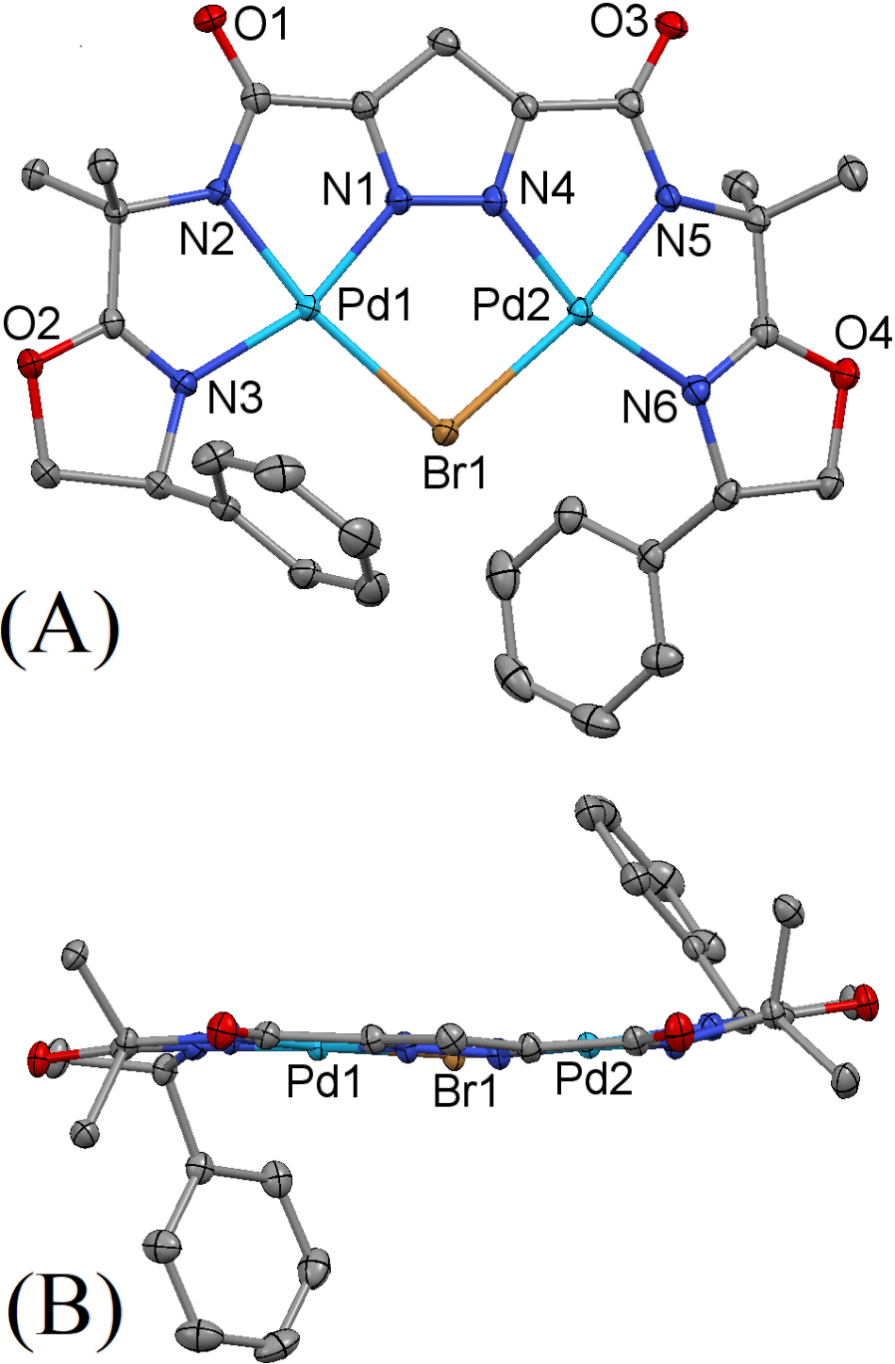
Thermal ellipsoid plot (50% probability) of the molecular structure of **30**·Pd_2_Br. Hydrogen and solvate atoms have been omitted for clarity: (A) view normal to the mean molecular plane; (B) side view nearly perpendicular to (A) to allow a better view of the region of open access to the metal site. The complex **30**·Pd_2_Br crystallizes in the monoclinic space group *P*2_1_ with *a* = 9.8982(7) Å, *b* = 10.0139(7) Å, *c* = 16.9675(12) Å, α = 90°, β = 101.277(1)°, γ = 90°, *V* = 1649.3(2) Å^3^, *Z* = 2, *D_c_* = 1.847 mg m^−3^ and μ(Mo Kα) = 2.510 mm^−1^.

Initial experiments have shown that the trianionic ligand **30**-H_3_ readily forms complexes with various nickel, copper and palladium salts. Figure 5 shows the X-ray crystal structure of **30**·Pd_2_Br, obtained from ligand **30**-H_3_ and two equivalents of palladium(ΙΙ) bromide. Each of the two palladium(ΙΙ) centers of the complex **30**·Pd_2_Br is confined in a slightly distorted square planar geometry by three donor nitrogen atoms from the ligand and a bridging bromide ion. Unlike the situation in **16**·Ni_2_(OAc)_2_, where the Ni–O bond from the carbonyl of an adjacent molecule yields both the five-coordinate Ni and 1D polymeric chain, there are no Pd–O bonds (not even dative) in **30**·Pd_2_Br and the Pd atoms remain nearly square planar. The donor nitrogen atoms from pyrazole, amide and oxazoline moieties coordinate to the palladium centers, forming five-membered chelate rings, with the subtended N–Pd–N angles ranging from 79.43° to 82.16°. The two palladium centers are bridged by the bromide ion and the pyrazole segment. All of the Pd–N bond lengths fall within a relatively narrow range of 1.925 to 2.025 Å, and are typical for Pd–N bonds with this coordination geometry. As expected, the Pd–Br bond lengths are slightly longer (2.519 and 2.529 Å) than the Pd–N bonds. The bromide ion bridges the two palladium centers with a Pd–Br–Pd angle of 98.48°. The two nitrogen donor atoms from the pyrazole moiety forms the second bridging link between the two metal centers, thereby restricting the N(1)–Pd(1)–Br(1) and N(4)–Pd(2)–Br(1) angles to 89.89° and 89.73°, respectively. The resulting Pd(1)∙∙∙Pd(2) distance was observed to be 3.824 Å.

**Scheme 5:**
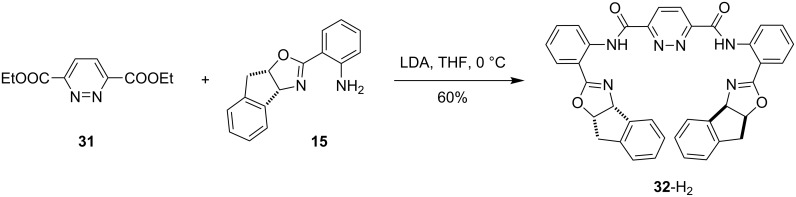
Synthesis of pyridazine–bisoxazoline ligand **32**-H_2_.

**Pyridazine-bridged bisoxazoline ligands.** Ligand **32**-H_2_ was synthesized from pyridazine diester **31** [60] and aminophenyloxazoline **15** (Scheme 5). Treatment of **15** with LDA, followed by addition of **31**, resulted in the formation of the pyridazine bridged bisoxazoline ligand **32**-H_2_.

**Figure 6:**
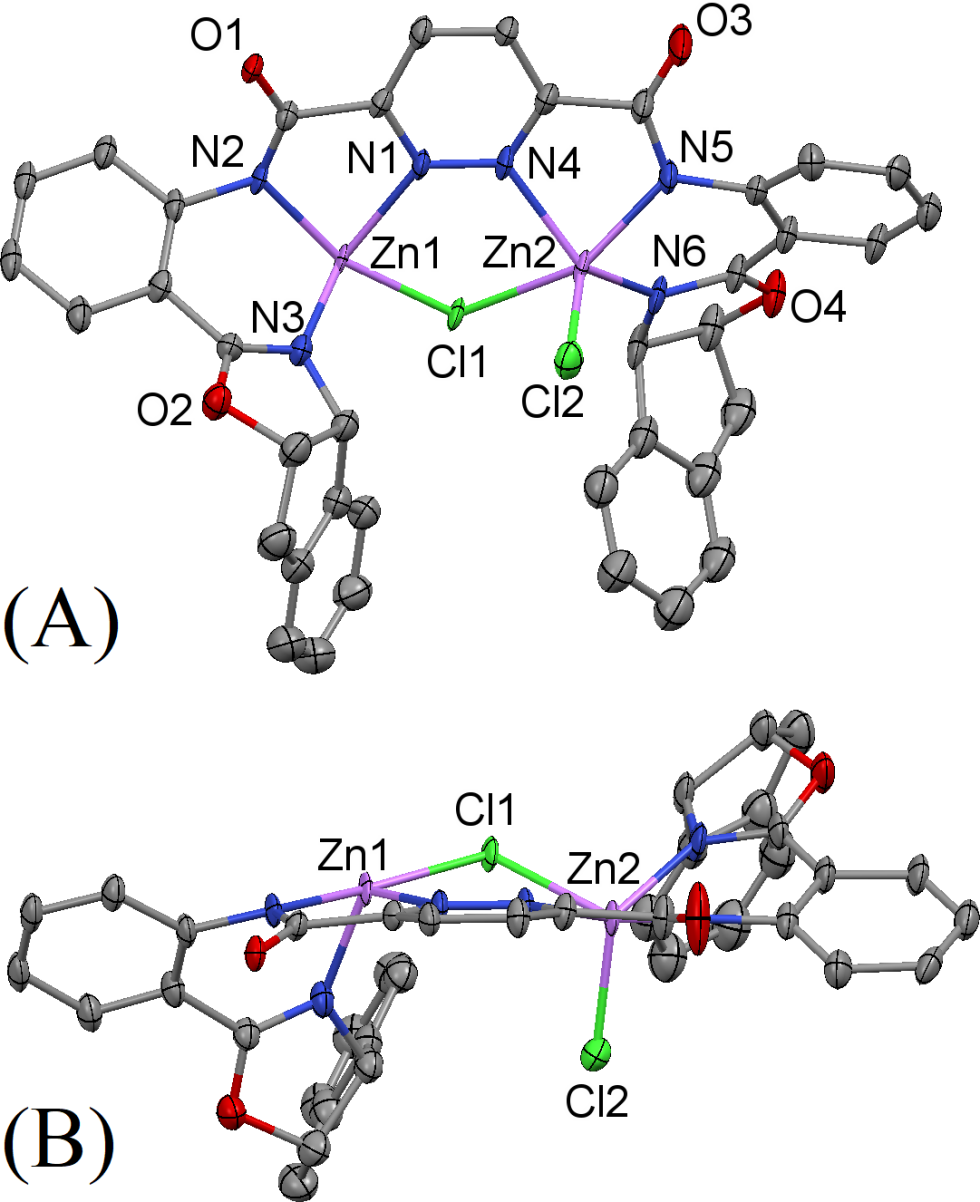
Thermal ellipsoid plot (50% probability) of the molecular structure of **32**·Zn**_2_**Cl**_2_**. Hydrogen and solvate atoms have been omitted for clarity: (A) view normal to the mean molecular plane; (B) side view nearly perpendicular to (A) to allow better view of the region of open access to the metal site. The complex **32**·Zn_2_Cl_2_ crystallizes in the monoclinic space group *C*_2_ with *a* = 40.820(5) Å, *b* = 7.4084(9) Å, *c* = 14.6489(17) Å, α = 90°, β = 108.589(2)°, γ = 90°, *V* = 4189.8(9) Å^3^, *Z* = 4, *D_c_* = 1.575 mg m^−3^ and μ(Mo Kα) = 1.453 mm^−1^.

Upon deprotonation, **32**-H_2_ provides a dianionic ligand with three donor nitrogen atoms per metal center. Ligand **32**-H_2_ forms complexes with different nickel, copper, zinc and palladium salts. Shown in Figure 6 is the molecular structure of **32**·Zn_2_Cl_2_. Each unit of the complex **32**·Zn_2_Cl_2_ consists of two zinc(ΙΙ) centers, with each bound by three donor nitrogen atoms from the ligand skeleton and bridged by a chloride ion. The nitrogen atoms on the pyridazine and amide moieties form five-membered chelate rings upon coordination to the zinc centers, subtending N(2)–Zn(1)–N(1) and N(5)–Zn(2)–N(4) angles of 74.74° and 77.04°, respectively. The coordination of the nitrogen atoms on oxazoline and amide moieties with the two zinc centers forms six-membered rings with N(3)–Zn(1)–N(2) and N(6)–Zn(2)–N(5) angles of 87.05° and 88.55°, respectively. All of the Zn–N distances are within 2.023–2.177 Å, as expected for such complexes. Interestingly, the two chloride ions bind in discrete manners. While one of them acts as a bridge between the two zinc centers with a Zn(1)–Cl(1)–Zn(2) angle of 105.97°, the other chloride ion binds to Zn(2) only with a Zn(2)–Cl(2) distance of 2.241 Å. Consistent with having a bridging chloride atom, the Zn(1)∙∙∙Zn(2) distance of 3.857 Å is somewhat long, compared to the M∙∙∙M distances in two of the crystal structures mentioned here, namely, the Ni∙∙∙Ni in **16**·Ni_2_(OAc)_2_ at 3.338 Å, shorter because of the smaller radius of the bridging μ_2_-O atom, and the **30**·Pd_2_Br at 3.824 Å, shorter because of both the Pd coordination geometry and the bridging μ_2_*-*Br atom. The Ni∙∙∙Ni in **25**·Ni_2_(OAc) at 4.176 Å is longer due to its longer three-atom bridge from OAc. In the Cambridge Structural Database (CSD), there are ten instances of Ni–N–N–Ni bond connectivity with sp^2^ N-atoms that are also bonded to a C-atom. However, all of those have *trans* related Ni atoms unlike the *cis* related here such that the Ni∙∙∙Ni distances range from 4.54 to 4.72 Å for those ten compounds. Similarly, for Pd–N–N–Pd and Zn–N–N–Zn, there are few and only all *trans* situations in the CSD with ranges of 4.83 to 4.84 Å and 4.53 to 4.78 Å, respectively, for the two Pd and three Zn compounds. For M–N–N–M with sp^3^ bridging N-atoms, only four instances are found – all with M = Zn and N–N from substituted aminohydrazido ligands and the distances are longer (4.93 to 5.04 Å) and the *trans* relationship maintains. Thus, the *cis* M–N–N–M geometry observed here is quite rare for M = Ni, Zn, Pd and is uncommon for M = any transition metal (about 90 instances in CSD). Different coordination environments are found for Zn(1) and Zn(2) in **32**·Zn_2_Cl_2_. Zn(1) is found to exist in a square planar environment that experiences a significant tetrahedral distortion. In contrast, a distorted square pyramidal binding mode is observed for Zn(2).

**Scheme 6:**
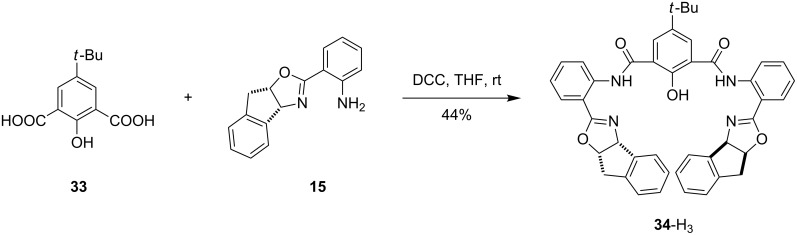
Synthesis of phenol–bisoxazoline ligand **34**-H_3_.

**Phenol-bridged bisoxazoline ligands.** Ligands incorporating naphthyridine, pyridazine and pyrazole linkers discussed thus far bridge two metal atoms by attachment to two different nitrogen donor atoms. As a result, metal∙∙∙metal distances tend to be relatively long. Ligand **34**-H_3_ was designed to explore the effect of a single atom linker, namely a phenoxy bridge. The *tert*-butyl group in the *para*-position was incorporated as it would likely increase the overall solubility of the ligand as well as its associated complexes. DCC-mediated coupling of **33** [49] with two equivalents of aminophenyloxazoline **15** led to the formation of phenol-bridged bisoxazoline ligand **34**-H_3_ in a single step and moderate yield (Scheme 6). While X-ray quality crystals have not yet been obtained, preliminary experiments have shown that ligand **34**-H_2_ forms complexes with nickel, copper and palladium salts [58].

## Conclusion

We have achieved the synthesis of chiral bisoxazoline ligands that incorporate naphthyridine, pyrazole, pyridazine and phenol bridges. These compounds readily form complexes with various transition metal salts and may provide a platform for the development of new catalytic enantioselective transformations.

## Supporting Information

File 1 Experimental procedures and characterization data.

File 2 Crystallographic information files of synthesized complexes (CCDC 1568876–1568879).
