# Supplementary material for: Chiral bisoxazoline ligands designed to stabilize bimetallic complexes
Source: Beilstein J Org Chem. 2018 Aug 1;14:2002–11. doi: 10.3762/bjoc.14.175 (PMC6122208; doi:10.3762/bjoc.14.175)

Supporting Information

for

Chiral bisoxazoline ligands designed to stabilize bimetallic complexes

Deepankar Das[1], Rudrajit Mal[1], Nisha Mittal[1], Zhengbo Zhu[2], Thomas J. Emge[1] and Daniel Seidel[1,2,*]

Address: [1]Department of Chemistry and Chemical Biology, Rutgers, The State University of New Jersey, Piscataway, NJ 08854, USA and [2] Center for Heterocyclic Compounds, Department of Chemistry, University of Florida, Gainesville, Florida 32611, USA

Email: Daniel Seidel - seidel@chem.ufl.edu

* Corresponding author

Experimental procedures and characterization data

General Information: Starting materials, reagents and solvents were purchased from commercial sources and were used as received. Dichloromethane was distilled over calcium hydride prior to use. Tetrahydrofuran was distilled over sodium/benzophenone prior to use. Methanol was distilled over magnesium methoxide prior to use. Reactions were run under an atmosphere of dry nitrogen unless otherwise mentioned. Analytical thin-layer chromatography was performed on EM Reagent 0.25 mm silica gel 60 F_{254} plates. Visualization was accomplished with UV light, potassium permanganate, and Dragendorff–Munier stains followed by heating. Purification of reaction products was carried out by flash column chromatography using Sorbent Technologies Standard Grade silica gel (60 Å, 230–400 mesh). Infrared spectra were recorded on an ATI Mattson Genesis Series FT-Infrared spectrophotometer. Melting points were recorded on a Electrothermal Mel-Temp 3.0 melting point apparatus and are uncorrected. Proton nuclear magnetic resonance spectra (^{1}H NMR) were recorded on a Varian VNMRS500 MHz and Varian VNMRS-400 MHz instrument and are reported in ppm using the solvent as an internal standard ($CDCl_3$ at 7.26 ppm, $(CD_3)_2SO$ at 2.50 ppm). Data are reported as app = apparent, br = broad, s = singlet, d = doublet, t = triplet, q = quartet, dd = doublet of doublets, m = multiplet, comp = complex, and coupling constant(s) are reported in Hz. Proton-decoupled carbon nuclear magnetic resonance spectra (^{13}C NMR) were recorded on a Varian VNMRS-500 MHz and VNMRS-400 MHz instrument and are reported in ppm using the solvent as an internal standard ($CDCl_3$ at 77.0 ppm, $(CD_3)_2SO$ at 39.5 ppm). HRMS spectrometry data were recorded on a Bruker Daltonics Apex-ultra 70 spectrometer operating on ESI-FTICR (MeCN, MeOH or DMSO as solvent). Optical rotations were measured using a 1 mL cell with a 1 dm path length on a Jasco P-2000 polarimeter at 589 nm and at 25 °C. Compounds **15**,[1] **24**,[2] **31**[3] and **33**[4] were synthesized according to known procedures.

***Synthesis and characterization data of Compound* 16–H$_2$:** A solution of the 1,8-naphthyridine 2,7-dicarboxylic acid (1.38 mmol, 0.3 g) in thionyl chloride (5 mL) was heated under reflux for 2 h. Excess solvent was then distilled off and the crude **14** dried under vacuum. To a solution of the crude **14** in anhydrous dichloromethane (14 mL), amine **15** (2.89 mmol, 0.723 g) was added followed by dropwise addition of triethylamine (4.13 mmol, 0.57 mL) and the reaction mixture allowed to stir at room temperature for 24 h. The mixture was then washed with 10% hydrochloric acid and water. The organic layer was separated, dried with anhydrous sodium sulfate and the solvent evaporated in vacuo. The crude residue was purified by flash column chromatography to give the title compound as a white solid in 65% yield (R_f = 0.18 in hexanes/EtOAc 75:25 v/v). mp: >230 °C; $[\alpha]_D^{25}$ –1317.7 (c 0.5, $CHCl_3$); IR (KBr) 2959, 1734, 1682, 1635, 1582, 1539, 1448, 1353, 1282 cm^{-1}; ^{1}H NMR (400 MHz, $CDCl_3$) δ 14.35 (s, 2H), 8.94 (d, *J* = 8.4 Hz, 2H), 8.61 (d, *J* = 8.3 Hz, 2H), 8.52 (d, *J* = 8.5 Hz, 2H), 7.96 (d, *J* = 7.5 Hz, 2H), 7.92 (d, *J* = 8.0 Hz, 2H), 7.55–7.46 (comp, 2H), 7.18–7.07 (comp, 4H), 7.03–6.95 (comp, 2H), 6.68–6.60 (comp, 2H), 6.24 (d, *J* = 7.8 Hz, 2H), 5.52–5.43 (m, 2H), 3.49 (dd, *J* = 18.0, 6.7 Hz, 2H), 3.36 (d, *J* = 18.0 Hz, 2H); ^{13}C NMR (100 MHz, $CDCl_3$) δ 163.6, 162.9, 154.9, 153.2, 142.1, 139.2, 139.0, 138.6, 132.1, 129.3, 128.1, 127.0, 126.6, 125.3, 124.7, 123.0, 121.4, 120.9, 115.4, 81.9, 77.1, 39.8; HRMS (ESI) *m/z* calculated for $C_{42}H_{31}N_6O_4$ $[M + H]^+$ 683.2401, found 683.2438.

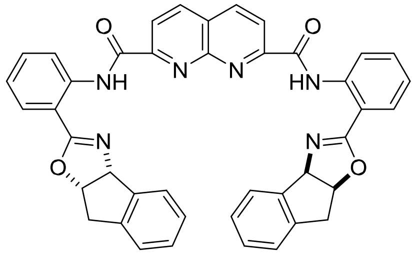

***Preparation of* 16•Ni$_2$(OAc)$_2$**

Compound **16–H$_2$** (0.088 mmol, 0.060 g) was dissolved in 3 mL of anhydrous methanol. Nickel(II) acetate tetrahydrate (0.180 mmol, 0.045 g) and triethylamine (0.180 mmol, 0.025 mL) were added and the resulting mixture stirred under reflux for 2 h. The reaction mixture was then cooled to room temperature and methanol evaporated off. The residue was dissolved in 10 mL of dichloromethane and filtered through a filter paper. The filtrate was then washed with water. The aqueous portion was washed twice with dichloromethane. The combined organics were dried with anhydrous sodium sulfate, filtered and concentrated to obtain the complex as an orange-brown solid.
A portion of the complex was dissolved in 1,2-dichloroethane and layered with heptane. Crystals suitable for single X-ray crystallographic analysis were obtained after several days.

***Synthesis and characterization data of Compound* 19:** *N*-Cbz glycine **18** (4.78 mmol, 1 g) was taken in dichloromethane (40 mL) and a few drops of methanol was added to dissolve the amino acid completely. The solution was cooled in an ice bath and *N*-methylmorpholine (5.23 mmol, 0.58 mL) and isobutylchloroformate (5.23 mmol, 0.69 mL) were added consecutively and stirred at 0 °C for 1 h. (*S*)-2-Phenylglycinol **17** (5.23 mmol, 0.72 g; dissolved in 8 mL of dichloromethane) was then added dropwise and the reaction mixture gradually warmed to room temperature. After stirring for 10 h, the reaction mixture was washed with 1 M hydrochloric acid. Combined organics were dried over anhydrous sodium sulfate, filtered, concentrated and purified by flash chromatography to give the product as a white solid in 96% yield (R_f = 0.35 in EtOAc/MeOH 98:2 v/v). mp: 105–107 °C; $[\alpha]_D^{25}$ +10.96 (c 0.5, $CHCl_3$); IR

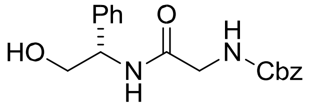

(KBr) 3349, 2925, 1694, 1660, 1538, 1249, 1058 cm^{-1}; ^{1}H NMR (500 MHz, $CDCl_3$) δ 7.37–7.27 (comp, 6H), 7.26–7.16 (comp, 4H), 5.87–5.80 (m, 1H), 5.07 (s, 2H), 5.07–5.01 (m, 1H), 3.98–3.91 (m, 1H), 3.87–3.80 (comp, 2H), 3.80–3.69 (comp, 2H); ^{13}C NMR (125 MHz, $CDCl_3$) δ 169.6, 156.9, 138.6, 136.0, 128.7, 128.5, 128.2, 128.0, 127.7, 126.6, 67.2, 65.7, 55.6, 44.5; HRMS (ESI) *m/z* calculated for $C_{18}H_{20}N_2NaO_4$ $[M + Na]^+$ 351.1315, found 351.1335.

***Synthesis and characterization data of Compound* 20:** To a solution of **19** (3.05 mmol, 1g) in 30 mL of anhydrous methanol, 0.15 g of 10% palladium on activated charcoal was added and the flask purged with hydrogen gas five times. The reaction mixture was then allowed to stir at room temperature under an atmosphere of hydrogen. After 5 h, the mixture was filtered through a pad of celite® and washed with methanol. After concentrating the filtrate, product **20** was obtained as a solid in near quantitative yield. $[\alpha]_D^{25}$ –6.2 (c 0.5, $CHCl_3$); IR (KBr) 3401, 2922, 1634, 1392, 1151, 1019 cm^{-1}; ^{1}H NMR (500 MHz, $CDCl_3$) δ 7.92 (br s, 1H), 7.40– 7.33 (comp, 2H), 7.33–7.27 (comp, 3H), 5.11–5.01 (m, 1H), 3.93–3.77 (comp, 2H), 3.39 (d, J = 17.3 Hz, 1H), 3.34 (d, J = 17.3 Hz, 1H), 1.54 (br s, 2H); ^{13}C NMR (125 MHz, $CDCl_3$) δ 173.5, 138.9, 128.8, 127.8, 126.7, 66.8, 55.9, 44.7; HRMS (ESI) *m/z* calculated for $C_{10}H_{15}N_2O_2$ $[M + H]^+$ 195.1128, found 195.1143.

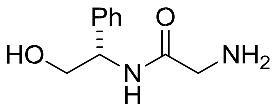

***Synthesis and characterization data of Compound* 21:** A solution of the 1,8-naphthyridine 2,7-dicarboxylic acid (0.92 mmol, 0.2 g) in thionyl chloride (4 mL) was heated under reflux for 2 h. Excess solvent was then distilled off and crude **14** dried under vacuum. A solution of the crude **14** and Hünig's base (2.75 mmol, 0.48 mL) in anhydrous dichloromethane (7 mL) was cooled in an ice bath and a solution of **20** (1.93 mmol, 0.364 g) in 2 mL dichloromethane was added dropwise. The reaction mixture was then gradually warmed to room temperature and allowed to stir for 24 h. Upon addition of water, the desired product precipitated out as an off-white solid which was then filtered out, washed with dichloromethane and dried. mp: 195–199 °C; IR (KBr) 3316, 3064, 2935, 1659, 1600, 1532, 1495, 1425, 1270 cm^{-1}; ^{1}H NMR (500 MHz, dmso-d_6) δ 9.05 (app t, J = 5.7 Hz, 2H), 8.80 (d, J = 8.4 Hz, 2H), 8.48 (d, J = 8.2 Hz, 2H), 8.35 (d, J = 8.5 Hz, 2H), 7.41–7.28 (comp, 8H), 7.27–7.20 (comp, 2H), 4.96–4.85 (comp, 4H), 4.18–4.06 (comp, 4H), 3.64–3.53 (comp, 4H); ^{13}C NMR (125 MHz, dmso-d_6) δ 167.9, 163.4, 153.5, 152.5, 140.9, 139.9, 128.1, 126.9, 126.8, 125.1, 120.7, 64.6, 55.2, 42.5; HRMS (ESI) *m/z* calculated for $C_{30}H_{30}N_6NaO_6$ $[M + Na]^+$ 593.2119, found 593.2142.

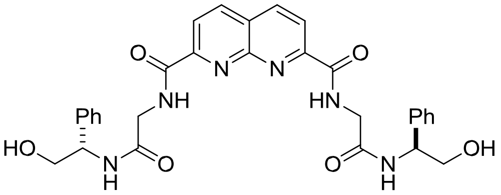

***Synthesis and characterization data of Compound* 22–H$_2$:** To a suspension of **21** (0.145 mmol, 0.080 g), *p*-toluenesulfonyl chloride (0.32 mmol, 0.061 g) and DMAP (0.029 mmol, 0.0035 g) in anhydrous dichloromethane, triethylamine (0.435 mmol, 0.061 mL) was added. The reaction mixture was stirred at room temperature for 24 h, after which it was poured into ice cold saturated aqueous solution of NH_4Cl and extracted with dichloromethane three times. The combined organic extracts were then dried with anhydrous sodium sulfate and solvent evaporated in vacuo. The crude mixture was then purified by flash column chromatography to give the title compound in 47% yield (R_f = 0.18 in EtOAc/MeOH 98:2 v/v). IR (KBr) 2982, 2902, 1680, 1550, 1052 cm^{-1}; ^{1}H NMR (400 MHz, $CDCl_3$) δ 9.02–8.94 (comp, 2H), 8.42 (d, *J* = 8.5 Hz, 2H), 8.32 (d, *J* = 8.4 Hz, 2H), 7.33–7.26 (comp, 4H), 7.25–7.19 (comp, 6H), 5.21–5.15 (m, 2H), 4.65 (dd, *J* = 10.2, 8.4 Hz, 2H), 4.51 (app d, *J* = 5.8 Hz, 4H), 4.12 (app t, *J* = 8.3 Hz, 2H); ^{13}C NMR (100 MHz, $CDCl_3$) δ 164.9, 163.9, 153.3, 152.6, 141.7, 138.6, 128.6, 127.5, 126.5, 125.3, 121.2, 75.3, 69.4, 37.0; *m*/*z* (ESI-MS) 557.1 [M + Na]$^+$.

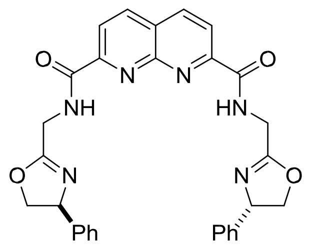

***Synthesis and characterization data of Compound* 25–H$_3$:** A solution of the pyrazole 3,5-dicarboxylic acid (1.60 mmol, 0.25 g) in thionyl chloride (4 mL) was heated under reflux for 2 h. Excess solvent was then distilled off and the crude **23** dried under vacuum. A solution of the crude pyrazole diacylchloride **23**, triethylamine (4.8 mmol, 0.67 mL) and DMAP (0.32 mmol, 0.039 g) in anhydrous dichloromethane (13 mL) was cooled in an ice bath and the amine **24** (3.28 mmol, 0.782 g) was added slowly as a solution in dichloromethane (3 mL). The reaction mixture was allowed to gradually warm up to room temperature and stirred overnight. The mixture was then washed with 10% hydrochloric acid and water, and the organic layer separated. The aqueous layer was further extracted two more times with dichloromethane. Combined organics were dried with anhydrous sodium sulfate, filtered, concentrated and purified by flash column chromatography to furnish the title compound a white solid in 85% yield. mp: 126 °C; $[\alpha]_D^{25}$ +359.7 (c 0.5, $CHCl_3$); IR (KBr) 3434, 3175, 3027, 2966, 1679, 1633, 1586, 1534, 1448, 1299 cm^{-1}; ^{1}H NMR (500 MHz, $CDCl_3$) δ 13.25 (s, 2H), 11.29 (br s, 1H), 8.98–8.72 (m, 2H), 7.96 (app d, *J* = 7.7 Hz, 2H), 7.61–7.50 (m, 2H), 7.41–7.35 (comp, 4H), 7.35–7.28 (comp, 4H), 7.27–7.21 (m, 2H), 7.20 (s, 1H), 7.19–7.13 (m, 2H), 5.65–5.52 (m, 2H), 4.84–4.74 (m, 2H), 4.45–4.25 (m, 2H); ^{13}C NMR (125 MHz, $CDCl_3$) δ 164.3, 141.2, 139.5, 132.8, 129.5, 128.8, 127.7, 126.4, 122.8, 120.1, 113.6, 105.8, 73.1, 69.6; HRMS (ESI) *m/z* calculated for $C_{35}H_{29}N_6O_4$ [M + H]$^+$ 597.2245, found 597.2266.

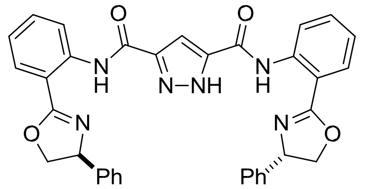

***Preparation of* 25•Ni$_2$(OAc)**

Compound **25**−H$_3$ (0.067 mmol, 0.040 g) was dissolved in 3 mL of anhydrous methanol. Nickel(II) acetate tetrahydrate (0.137 mmol, 0.034 g) and triethylamine (0.204 mmol, 0.028 mL) were added and the resulting mixture stirred under reflux for 2 h. The reaction mixture was then cooled to room temperature and methanol evaporated off. The residue was dissolved in 10 mL of dichloromethane and filtered through a filter paper. The filtrate was then washed with water. Aqueous portion washed twice with

dichloromethane. Combined organics was dried with anhydrous sodium sulfate, filtered and concentrated to obtain the complex as orange-brown solid.

A portion of the complex was dissolved in dichloromethane and layered with hexanes. Crystals suitable for single X-ray crystallographic analysis were obtained after several days.

***Synthesis and characterization data of Compound* 27:** *N*-Cbz-2-methylalanine **26** (8.43 mmol, 2 g) was dissolved in THF (70 mL) and the solution was cooled in an ice bath. *N*-Methylmorpholine (9.27 mmol, 1.02 mL) was then added, followed by isobutylchloroformate (9.27 mmol, 1.22 mL). The resulting mixture was allowed to stir at 0 °C for 1 h. (*S*)-2-Phenylglycinol **17** (8.43 mmol, 1.16 g; dissolved in 14 mL of THF) was then added dropwise and the reaction mixture gradually warmed to room temperature. After stirring for 10 h, the white solid formed was filtered out and the filtrate was washed with water. Combined organics were dried over anhydrous sodium sulfate, filtered, concentrated and purified by flash chromatography to give the product as a white solid in 90% yield (R_f = 0.30 in Hexanes/EtOAc 30:70 v/v). $[\alpha]_D^{25}$ +2.2 (c 0.5, $CHCl_3$); IR (KBr) 3306, 3032, 2941, 1538, 1519, 1258, 1088 cm^{-1}; ^{1}H NMR (500 MHz, $CDCl_3$) δ 7.36–7.26 (comp, 6H), 7.26– 7.16 (comp, 4H), 6.90 (br s, 1H), 5.63 (s, 1H), 5.12–4.96 (comp, 3H), 3.87–3.80 (m, 1H), 3.64 (dd, *J* = 11.6, 6.7 Hz, 1H), 3.05 (br s, 1H), 1.52 (s, 3H), 1.48 (s, 3H); ^{13}C NMR (125 MHz, $CDCl_3$) δ 174.3, 155.5, 138.9, 136.0, 128.6, 128.5, 128.2, 128.1, 127.6, 126.6, 66.9, 65.7, 57.1, 55.5, 25.9, 25.0; HRMS (ESI) *m/z* calculated for $C_{20}H_{24}N_2NaO_4$ $[M + Na]^+$ 379.1628, found 379.1633.

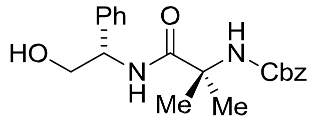

***Synthesis and characterization data of Compound* 29:** To a solution of **27** (2.24 mmol, 0.8 g), *p*-toluenesulfonyl chloride (2.47 mmol, 0.471 g) and DMAP (0.45 mmol, 0.055 g) in anhydrous dichloromethane, triethylamine (6.73 mmol, 0.94 mL) was added dropwise and allowed to stir at room temperature for 12 h. The reaction mixture was poured into an ice cold saturated aqueous solution of NH_4Cl and extracted with dichloromethane three times. The combined organic extracts were then dried with anhydrous sodium sulfate, filtered and the volatiles evaporated in vacuo. The crude mixture was then purified through a short pad of silica gel, eluting with 30:70 EtOAc/hexanes, to give the *N*-carboxybenzyl compound **28** which was used in the next step without further purification. Compound **28** was then dissolved in anhydrous methanol and after addition of 10% palladium on charcoal, the heterogenous mixture was stirred under hydrogen for 4 h. The reaction mixture was then filtered through a pad of celite and rinsed with methanol. After evaporating the solvent under vacuum, the crude mixture was purified by flash column chromatography to give the title compound as a colorless liquid in 85% yield over two steps (R_f = 0.15 in EtOAc/MeOH 90:10 v/v). $[\alpha]_D^{25}$ –14.4 (c 0.5, $CHCl_3$); IR (KBr) 3362, 2975, 1655, 1495, 1455, 1131 cm^{-1}; ^{1}H NMR (500 MHz, $CDCl_3$) δ 7.37–7.30 (comp, 2H), 7.28–7.24 (m, 1H), 7.23–7.14 (comp, 2H), 5.16 (dd, *J* = 10.0, 7.9 Hz, 1H), 4.64 (dd, *J* = 10.1, 8.5 Hz, 1H), 4.13 (dd, *J* = 8.3, 7.8 Hz, 1H), 1.76 (br s, 2H), 1.44 (s, 3H), 1.43 (s, 3H); ^{13}C NMR (125 MHz, $CDCl_3$) δ 174.7, 142.3, 128.7, 127.5, 126.4, 75.5, 69.4, 50.3, 28.5, 28.3; HRMS (ESI) *m/z* calculated for $C_{12}H_{16}N_2NaO$ $[M + Na]^+$ 227.1155, found 227.1177.

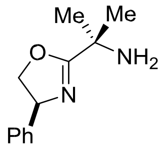

***Synthesis and characterization data of Compound* 30–H$_3$:** A solution of pyrazole 3,5-dicarboxylic acid (1.28 mmol, 0.2 g) in thionyl chloride (3 mL) was heated under reflux for 2 h. Excess solvent was then distilled off and the crude **23** dried under vacuum. The diacyl chloride **23** was dissolved in anhydrous dichloromethane (10 mL) along with DMAP (0.25 mmol, 0.031 g) and triethylamine (3.84 mmol 0.54 mL). The mixture was then cooled in an ice bath and a solution of **29** in 2 mL of dichloromethane was added dropwise. The reaction mixture was gradually warmed to room temperature and stirred for 24 h. The mixture was then washed with 10% hydrochloric acid and water, and the organic layer separated. The aqueous layer was further extracted two more times with dichloromethane. Combined organics were dried with anhydrous sodium sulfate, filtered, concentrated and purified by flash column chromatography. The title compound was obtained as a white solid in 30% yield. mp: 100–104 °C; IR (KBr) 2988, 2902, 1682, 1557, 1324, 1054 cm^{-1}; ^{1}H NMR (500 MHz, CDCl$_3$) δ 12.93 (br s, 1H), 7.66 (br s, 2H), 7.29–7.21 (comp, 4H), 7.21–7.12 (comp, 6H), 6.98 (s, 1H), 5.17 (dd, *J* = 9.7, 8.2 Hz, 2H), 4.67–4.57 (m, 2H), 4.15–4.07 (m, 2H), 1.70 (s, 12H) ; ^{13}C NMR (125 MHz, CDCl$_3$) δ 171.7, 141.9, 128.5, 127.5, 126.5, 105.2, 76.0, 69.0, 52.9, 25.4, 25.2; HRMS (ESI) *m/z* calculated for $C_{29}H_{32}N_6NaO_4$ [M + Na]$^+$ 551.2377, found 551.2414.

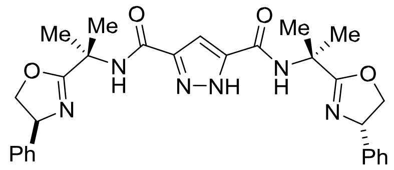

***Preparation of* 30•Pd$_2$Br**

Compound **30−H$_3$** (0.057 mmol, 0.030 g) was dissolved in 2 mL of anhydrous methanol. Palladium(II) bromide (0.116 mmol, 0.031 g) and triethylamine (0.174 mmol, 0.024 mL) were added and the resulting mixture stirred under reflux for 2 h. The reaction mixture was then cooled to room temperature and methanol evaporated off. The residue was dissolved in 7 mL of dichloromethane and filtered through a filter paper. The filtrate was then washed with water. The aqueous portion was washed twice with dichloromethane. Combined organics were dried with anhydrous sodium sulfate, filtered and concentrated to obtain the complex as orange-brown solid.
A portion of the complex was dissolved in 1,2-dichloroethane and layered with heptane. Crystals suitable for single X-ray crystallographic analysis were obtained after several days.

***Synthesis and characterization data of Compound* 32–H$_2$:** A solution of the amine **15** (2.81 mmol, 0.703 g) in anhydrous THF (8 mL) was cooled in an ice bath and freshly prepared LDA (1 M in THF, 2.81 mmol, 2.81 mL) was added slowly through cannula. After 5 mins, a solution of **31** (1.34 mmol, 0.3 g) in 2 mL of THF was added dropwise and the mixture gradually warmed to room temperature. After stirring for 24 h, the reaction mixture was quenched by the addition of ice cold saturated aqueous solution of NH$_4$Cl. The organic layer was isolated and aqueous part was extracted two times with EtOAc. Combined organic extracts were dried with anhydrous sodium sulfate, filtered, concentrated and purified by flash column chromatography to give the title compound as a white solid in 60% yield (R_f = 0.17 in hexanes/EtOAc 75:25 v/v). mp: 162–165 °C; $[\alpha]_D^{25}$ –523.3 (c 0.5, CHCl$_3$); IR (KBr) 3023, 2958, 1686, 1636, 1603, 1585, 1526, 1449, 1231 cm^{-1}; ^{1}H NMR (500 MHz, CDCl$_3$) δ 14.71 (s, 2H), 9.03 (d, *J* = 8.3 Hz, 2H), 8.56 (s, 2H), 8.45 (d, *J* = 7.5 Hz, 2H),

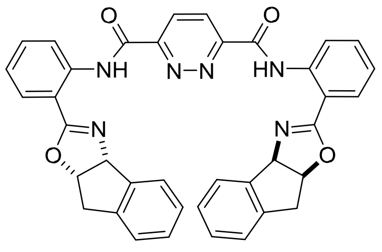

7.99–7.90 (comp, 2H), 7.59–7.52 (comp, 2H), 7.30–7.15 (comp, 8H), 5.93 (d, *J* = 7.8 Hz, 2H), 5.41–5.32 (m, 2H), 3.48 (dd, *J* = 18.2, 6.6 Hz, 2H), 3.38 (d, *J* = 18.1 Hz, 2H); ^{13}C NMR (125 MHz, $CDCl_3$) δ 163.1, 161.6, 154.6, 142.1, 139.3, 139.1, 132.4, 129.4, 128.6, 127.7, 127.0, 126.7, 125.1, 123.2, 120.6, 115.0, 81.9, 77.0(4), 39.6; HRMS (ESI) *m/z* calculated for $C_{38}H_{28}N_6NaO_4$ $[M + Na]^+$ 655.2064, found 655.2088.

***Preparation of* 32•2ZnCl**

Compound **32−H₂** (0.063 mmol, 0.040 g) was dissolved in 3 mL of anhydrous methanol. Zinc chloride (0.130 mmol, 0.018 g) and triethylamine (0.130 mmol, 0.018 mL) were added and the resulting mixture stirred under reflux for 2 h. The reaction mixture was then cooled to room temperature and methanol evaporated off. The residue was dissolved in 10 mL of dichloromethane and filtered through a filter paper. The filtrate was then washed with water. The aqueous portion was washed twice with dichloromethane. Combined organics were dried with anhydrous sodium sulfate, filtered and concentrated to obtain the complex as white solid.
A portion of the complex was dissolved in 1,2-dichloroethane/methanol (4:1) and layered with hexanes. Crystals suitable for single X-ray crystallographic analysis were obtained after several days.

***Synthesis and characterization data of Compound* 34–H₃:** To a stirring solution of the phenol dicarboxylic acid **33** (1.05 mmol, 0.25 g) and DCC (2.20 mmol, 0.455 g) in anhydrous THF (8 mL), a solution of the amine **15** (2.20 mmol, 0.551 g) in 2 mL THF was added over 15 min. The resulting milky white reaction mixture was allowed to stir for another 5 h, after which the white precipitate was filtered out and rinsed with THF. Solvent was evaporated under reduced pressure and product was purified by flash column chromatography to give the title compound as a white solid in 44% yield (R_f = 0.19 in hexanes/EtOAc 75:25 v/v). mp: 134–137 °C; $[\alpha]_D^{25}$ –324.3 (c 0.5, $CHCl_3$); IR (KBr) 3026, 2958, 2868, 1668, 1629, 1610, 1535, 1448, 1354, 1277, 1175, 1063, 1001 cm^{-1}; ^{1}H NMR (500 MHz, $CDCl_3$) δ 13.46 (br s, 1H), 13.02 (br s, 2H), 8.87 (d, *J* = 8.6 Hz, 2H), 8.32 (s, 2H), 7.90 (d, *J* = 8.0 Hz, 2H), 7.55–7.48 (comp, 2H), 7.47–7.43 (comp, 2H), 7.30–7.20 (comp, 6H), 7.15–7.09 (comp, 2H), 5.85 (d, *J* = 7.7 Hz, 2H), 5.47–5.40 (m, 2H), 3.50 (dd, *J* = 18.0, 6.5 Hz, 2H), 3.41 (d, *J* = 18.0 Hz, 2H), 1.55 (s, 9H); ^{13}C NMR (125 MHz, $CDCl_3$) δ 167.3, 163.9, 158.2, 141.4, 141.1, 139.4, 139.3, 132.4, 130.4, 129.5, 128.6, 127.7, 125.4, 125.3, 122.8, 121.0, 120.0, 114.6, 82.2, 76.7, 39.3, 34.5, 31.6; HRMS (ESI) *m/z* calculated for $C_{44}H_{38}N_4NaO_5$ $[M + Na]^+$ 725.2734, found 725.2770.

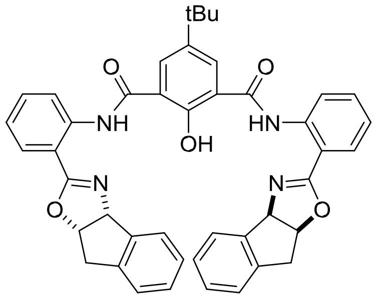

References:

(1) S. Doherty, J. G. Knight, A. McRae, R. W. Harrington and W. Clegg *Eur. J. Org. Chem.* **2008**, 1759.

(2) E. Wolińska *Tetrahedron: Asymmetry* **2014**, *25*, 1478.

(3) Y. Bessard, R. Crettaz, W. Brieden. European Patent Office. PCT Int. Appl. 2001007415, February 01, 2001.

(4) C. J. Fahrni and A. Pfaltz *Helv. Chim. Acta* **1998**, *81*, 491.

Figure S1. ORTEP diagram of **16•Ni2OAc2** with H atoms and solvate atoms removed and ellipsoids drawn at the 30% level.

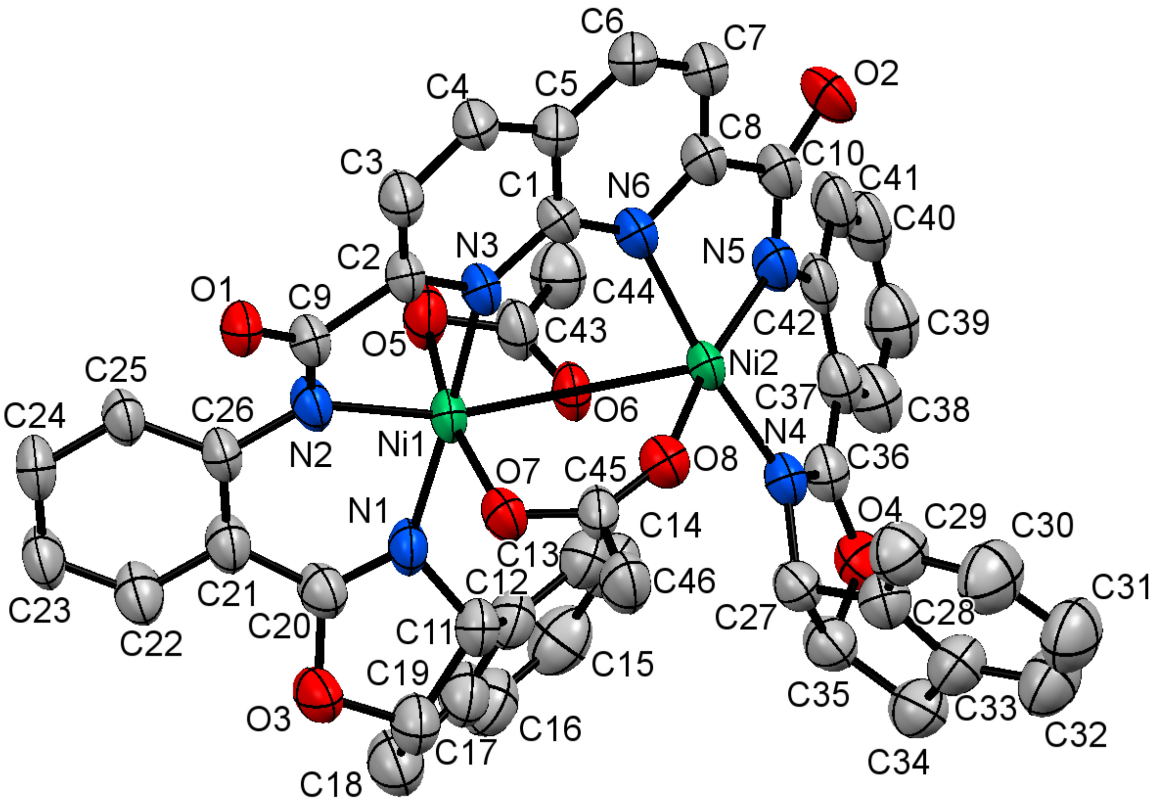

Table S1. Crystal data and structure refinement for **16•Ni2OAc2**.

Identification code	16-Ni2OAc2
Empirical formula	C51 H42 Cl5 N6 Ni2 O8
Formula weight	1161.57
Temperature	100(2) K
Wavelength	0.71073 Å
Crystal system	Orthorhombic
Space group	$P2_12_12_1$
Unit cell dimensions	a = 14.1829(5) Å α= 90°.
b = 14.8645(6) Å	β= 90°.
c = 25.2342(10) Å	γ = 90°.
Volume	5319.9(4) $Å^3$
Z	4
Density (calculated)	1.450 Mg/m^3
Absorption coefficient	1.017 mm^{-1}
F(000)	2380
Crystal size	0.210 x 0.120 x 0.080 mm^3
Theta range for data collection	1.590 to 27.875°.
Index ranges	-18<=h<=18, -19<=k<=19, -32<=l<=33
Reflections collected	56048
Independent reflections	12687 [R(int) = 0.0654]
Completeness to theta = 25.242°	100.0 %
Absorption correction	Semi-empirical from equivalents
Max. and min. transmission	0.7459 and 0.6655
Refinement method	Full-matrix least-squares on F^2
Data / restraints / parameters	12687 / 1046 / 642
Goodness-of-fit on F^2	1.051
Final R indices [I>2sigma(I)]	R1 = 0.0945, wR2 = 0.2581
R indices (all data)	R1 = 0.1290, wR2 = 0.2838
Absolute structure parameter	0.025(7)
Extinction coefficient	n/a
Largest diff. peak and hole	1.524 and -1.162 e.$Å^{-3}$

Table S2. Atomic coordinates (x 10^4) and equivalent isotropic displacement parameters (Å^{2}x 10^3) for **16•Ni2OAc2**. U(eq) is defined as one third of the trace of the orthogonalized U^{ij} tensor.

x	y	z	U(eq)	
Ni(1)	4820(1)	4598(1)	3435(1)	45(1)
Ni(2)	4492(1)	6888(1)	3320(1)	45(1)
O(1)	5769(5)	2766(4)	2361(3)	53(2)
O(2)	6561(6)	8733(5)	3252(4)	72(2)
O(3)	2987(6)	2890(5)	4212(3)	63(2)
O(4)	2745(6)	7988(5)	4479(3)	60(2)
N(1)	3988(6)	4040(5)	4018(3)	49(2)
N(2)	5208(6)	3383(5)	3149(3)	49(2)
N(3)	5599(6)	5043(5)	2764(3)	48(2)
N(4)	3411(6)	7355(5)	3761(3)	49(2)
N(5)	5322(6)	7872(5)	3596(4)	57(2)
N(6)	5732(6)	6576(5)	2927(4)	55(2)
C(1)	5952(7)	5863(6)	2616(4)	49(2)
C(2)	5811(7)	4340(6)	2458(4)	47(2)
C(3)	6336(8)	4394(7)	1986(5)	56(2)
C(4)	6734(8)	5205(7)	1845(5)	58(2)
C(5)	6579(8)	5959(7)	2167(5)	60(2)
C(6)	6973(8)	6812(7)	2087(5)	62(2)
C(7)	6807(8)	7499(7)	2417(5)	60(2)
C(8)	6173(8)	7351(7)	2850(5)	59(2)
C(9)	5585(7)	3405(6)	2663(4)	46(2)
C(10)	6013(7)	8083(7)	3259(5)	60(2)
C(11)	3169(8)	4465(7)	4282(4)	54(2)
C(12)	3353(8)	4945(7)	4798(4)	57(2)
C(13)	3720(8)	5779(8)	4880(5)	61(2)
C(14)	3804(9)	6141(10)	5399(5)	69(3)
C(15)	3436(10)	5622(10)	5814(5)	77(3)
C(16)	3051(11)	4808(10)	5737(5)	76(3)
C(17)	2975(10)	4448(8)	5206(5)	67(2)
C(18)	2490(11)	3608(9)	5050(6)	78(3)
C(19)	2531(10)	3690(7)	4422(5)	66(2)

C(20)	3839(8)	3181(7)	4013(4)	54(2)
C(21)	4464(8)	2473(7)	3840(4)	56(2)
C(22)	4376(9)	1654(7)	4106(4)	62(2)
C(23)	4917(9)	918(7)	3973(5)	62(2)
C(24)	5586(9)	996(7)	3566(5)	59(2)
C(25)	5680(7)	1798(6)	3288(4)	52(2)
C(26)	5113(7)	2557(6)	3416(4)	50(2)
C(27)	2404(7)	7121(7)	3732(4)	51(2)
C(28)	1866(8)	7686(7)	3345(5)	58(2)
C(29)	2032(10)	7778(9)	2810(5)	71(3)
C(30)	1447(11)	8391(12)	2542(7)	92(3)
C(31)	812(12)	8991(12)	2838(7)	94(3)
C(32)	656(11)	8827(11)	3333(7)	88(3)
C(33)	1179(9)	8178(9)	3601(5)	70(2)
C(34)	1132(9)	7911(9)	4181(5)	69(3)
C(35)	2009(9)	7397(8)	4274(5)	60(2)
C(36)	3525(8)	7857(6)	4181(4)	52(2)
C(37)	4380(9)	8305(6)	4364(5)	57(2)
C(38)	4332(11)	8716(8)	4852(5)	71(3)
C(39)	5079(11)	9173(8)	5086(6)	75(3)
C(40)	5900(10)	9165(7)	4823(6)	71(3)
C(41)	5991(10)	8793(7)	4330(5)	67(2)
C(42)	5223(8)	8344(6)	4086(5)	56(2)
O(5)	5990(6)	4869(5)	3940(3)	62(2)
O(6)	4853(5)	5843(4)	3837(3)	51(1)
C(43)	5651(9)	5635(6)	4037(5)	55(2)
C(44)	6165(10)	6265(8)	4384(5)	66(3)
O(7)	3598(5)	4627(4)	3013(3)	53(2)
O(8)	3569(5)	6104(5)	2892(3)	53(2)
C(45)	3270(8)	5364(6)	2818(4)	52(2)
C(46)	2410(9)	5260(8)	2458(6)	69(3)
Cl(1A)	-1946(5)	2321(5)	3664(3)	81(2)
C(51A)	-2599(15)	3242(15)	3880(11)	92(7)
C(52A)	-1940(30)	4003(10)	3969(11)	180(10)
Cl(2A)	-1370(13)	3780(13)	4583(7)	178(6)
Cl(1B)	-2020(20)	3760(20)	4031(14)	276(10)

C(51B)	-1240(30)	4600(20)	3780(30)	210(13)
C(52B)	-337(19)	4130(20)	3674(15)	230(16)
Cl(2B)	460(20)	4980(20)	3446(12)	312(15)
Cl(3A)	5141(9)	2827(8)	5208(4)	137(4)
C(53A)	5550(20)	3931(17)	5067(19)	197(10)
C(54A)	6600(20)	3870(30)	5070(16)	199(10)
Cl(4A)	6960(14)	4690(14)	5540(8)	188(5)
Cl(3B)	5690(20)	3750(20)	6336(12)	240(11)
C(53B)	5720(30)	3980(50)	5644(15)	209(10)
C(54B)	6700(40)	3780(20)	5480(20)	196(10)
Cl(4B)	7270(20)	4850(20)	5448(12)	188(5)
Cl(7A)	8553(12)	6673(11)	3665(6)	161(5)
C(57A)	9010(40)	6152(19)	3083(11)	172(13)
C(58A)	9280(50)	6910(20)	2731(11)	185(11)
Cl(8A)	9479(15)	6429(15)	2097(6)	202(7)

Table S3. Bond lengths [Å] and angles [°] for **16•Ni2OAc2**.

Ni(1)-N(2)	2.022(8)	C(3)-C(4)	1.378(14)
Ni(1)-O(7)	2.034(7)	C(3)-H(3)	0.9500
Ni(1)-N(1)	2.059(9)	C(4)-C(5)	1.402(16)
Ni(1)-O(6)	2.112(6)	C(4)-H(4)	0.9500
Ni(1)-N(3)	2.129(8)	C(5)-C(6)	1.401(15)
Ni(1)-O(5)	2.131(8)	C(6)-C(7)	1.340(16)
Ni(2)-N(5)	2.003(9)	C(6)-H(6)	0.9500
Ni(2)-N(4)	2.017(8)	C(7)-C(8)	1.432(17)
Ni(2)-O(8)	2.058(7)	C(7)-H(7)	0.9500
Ni(2)-N(6)	2.073(9)	C(8)-C(10)	1.516(16)
Ni(2)-O(6)	2.093(7)	C(11)-C(19)	1.507(16)
Ni(2)-O(1)#1	2.188(7)	C(11)-C(12)	1.507(16)
O(1)-C(9)	1.245(11)	C(11)-H(11)	1.0000
O(1)-Ni(2)#2	2.189(7)	C(12)-C(13)	1.359(16)
O(2)-C(10)	1.240(12)	C(12)-C(17)	1.377(17)
O(3)-C(20)	1.379(14)	C(13)-C(14)	1.421(18)
O(3)-C(19)	1.455(13)	C(13)-H(13)	0.9500
O(4)-C(36)	1.352(14)	C(14)-C(15)	1.40(2)
O(4)-C(35)	1.459(14)	C(14)-H(14)	0.9500
N(1)-C(20)	1.294(13)	C(15)-C(16)	1.34(2)
N(1)-C(11)	1.481(13)	C(15)-H(15)	0.9500
N(2)-C(9)	1.338(13)	C(16)-C(17)	1.446(19)
N(2)-C(26)	1.408(12)	C(16)-H(16)	0.9500
N(3)-C(2)	1.334(12)	C(17)-C(18)	1.480(19)
N(3)-C(1)	1.368(11)	C(18)-C(19)	1.591(19)
N(4)-C(36)	1.307(13)	C(18)-H(18A)	0.9900
N(4)-C(27)	1.472(13)	C(18)-H(18B)	0.9900
N(5)-C(10)	1.336(15)	C(19)-H(19)	1.0000
N(5)-C(42)	1.427(15)	C(20)-C(21)	1.443(15)
N(6)-C(8)	1.325(13)	C(21)-C(22)	1.395(15)
N(6)-C(1)	1.354(13)	C(21)-C(26)	1.417(15)
C(1)-C(5)	1.448(15)	C(22)-C(23)	1.378(15)
C(2)-C(3)	1.406(15)	C(22)-H(22)	0.9500
C(2)-C(9)	1.518(12)	C(23)-C(24)	1.403(17)

C(23)-H(23)	0.9500	C(43)-C(44)	1.474(16)
C(24)-C(25)	1.390(14)	C(44)-H(44A)	0.9800
C(24)-H(24)	0.9500	C(44)-H(44B)	0.9800
C(25)-C(26)	1.423(13)	C(44)-H(44C)	0.9800
C(25)-H(25)	0.9500	O(7)-C(45)	1.289(12)
C(27)-C(28)	1.496(15)	O(8)-C(45)	1.193(12)
C(27)-C(35)	1.534(16)	C(45)-C(46)	1.527(17)
C(27)-H(27)	1.0000	C(46)-H(46A)	0.9800
C(28)-C(29)	1.378(17)	C(46)-H(46B)	0.9800
C(28)-C(33)	1.378(17)	C(46)-H(46C)	0.9800
C(29)-C(30)	1.406(19)	Cl(1A)-C(51A)	1.741(13)
C(29)-H(29)	0.9500	C(51A)-C(52A)	1.485(14)
C(30)-C(31)	1.47(2)	C(51A)-H(51A)	0.9900
C(30)-H(30)	0.9500	C(51A)-H(51B)	0.9900
C(31)-C(32)	1.29(2)	C(52A)-Cl(2A)	1.779(14)
C(31)-H(31)	0.9500	C(52A)-H(52A)	0.9900
C(32)-C(33)	1.392(19)	C(52A)-H(52B)	0.9900
C(32)-H(32)	0.9500	Cl(1B)-C(51B)	1.789(14)
C(33)-C(34)	1.518(19)	C(51B)-C(52B)	1.482(14)
C(34)-C(35)	1.479(18)	C(51B)-H(51C)	0.9900
C(34)-H(34A)	0.9900	C(51B)-H(51D)	0.9900
C(34)-H(34B)	0.9900	C(52B)-Cl(2B)	1.788(14)
C(35)-H(35)	1.0000	C(52B)-H(52C)	0.9900
C(36)-C(37)	1.458(16)	C(52B)-H(52D)	0.9900
C(37)-C(38)	1.376(17)	Cl(3A)-C(53A)	1.777(14)
C(37)-C(42)	1.387(18)	C(53A)-C(54A)	1.483(14)
C(38)-C(39)	1.391(19)	C(53A)-H(53A)	0.9900
C(38)-H(38)	0.9500	C(53A)-H(53B)	0.9900
C(39)-C(40)	1.34(2)	C(54A)-Cl(4A)	1.781(14)
C(39)-H(39)	0.9500	C(54A)-H(54A)	0.9900
C(40)-C(41)	1.367(19)	C(54A)-H(54B)	0.9900
C(40)-H(40)	0.9500	Cl(3B)-C(53B)	1.780(14)
C(41)-C(42)	1.419(16)	Cl(3B)-Cl(8A)#3	1.95(3)
C(41)-H(41)	0.9500	C(53B)-C(54B)	1.488(14)
O(5)-C(43)	1.260(12)	C(53B)-H(53C)	0.9900
O(6)-C(43)	1.276(14)	C(53B)-H(53D)	0.9900

C(54B)-Cl(4B)	1.780(14)	C(57A)-C(58A)	1.486(14)
C(54B)-H(54C)	0.9900	C(58A)-Cl(8A)	1.776(14)
C(54B)-H(54D)	0.9900	Cl(8A)-Cl(3B)#4	1.95(3)
Cl(7A)-C(57A)	1.784(14)		

N(2)-Ni(1)-O(7)	93.6(3)	C(20)-O(3)-C(19)	105.4(8)
N(2)-Ni(1)-N(1)	93.0(3)	C(36)-O(4)-C(35)	107.6(8)
O(7)-Ni(1)-N(1)	84.0(3)	C(20)-N(1)-C(11)	107.3(9)
N(2)-Ni(1)-O(6)	161.6(3)	C(20)-N(1)-Ni(1)	118.9(7)
O(7)-Ni(1)-O(6)	104.6(3)	C(11)-N(1)-Ni(1)	126.8(6)
N(1)-Ni(1)-O(6)	91.3(3)	C(9)-N(2)-C(26)	119.9(8)
N(2)-Ni(1)-N(3)	81.5(3)	C(9)-N(2)-Ni(1)	114.5(6)
O(7)-Ni(1)-N(3)	91.1(3)	C(26)-N(2)-Ni(1)	125.6(6)
N(1)-Ni(1)-N(3)	172.3(3)	C(2)-N(3)-C(1)	117.2(8)
O(6)-Ni(1)-N(3)	95.6(3)	C(2)-N(3)-Ni(1)	109.5(6)
N(2)-Ni(1)-O(5)	99.8(3)	C(1)-N(3)-Ni(1)	133.1(7)
O(7)-Ni(1)-O(5)	166.6(3)	C(36)-N(4)-C(27)	107.2(8)
N(1)-Ni(1)-O(5)	95.5(3)	C(36)-N(4)-Ni(2)	123.3(7)
O(6)-Ni(1)-O(5)	61.9(3)	C(27)-N(4)-Ni(2)	129.0(6)
N(3)-Ni(1)-O(5)	90.7(3)	C(10)-N(5)-C(42)	120.6(9)
N(5)-Ni(2)-N(4)	90.2(4)	C(10)-N(5)-Ni(2)	112.4(8)
N(5)-Ni(2)-O(8)	166.0(3)	C(42)-N(5)-Ni(2)	127.0(7)
N(4)-Ni(2)-O(8)	90.0(3)	C(8)-N(6)-C(1)	119.2(9)
N(5)-Ni(2)-N(6)	80.3(4)	C(8)-N(6)-Ni(2)	106.0(7)
N(4)-Ni(2)-N(6)	170.5(3)	C(1)-N(6)-Ni(2)	130.4(7)
O(8)-Ni(2)-N(6)	99.3(3)	N(6)-C(1)-N(3)	117.1(9)
N(5)-Ni(2)-O(6)	100.4(3)	N(6)-C(1)-C(5)	121.1(8)
N(4)-Ni(2)-O(6)	95.6(3)	N(3)-C(1)-C(5)	121.7(9)
O(8)-Ni(2)-O(6)	93.6(3)	N(3)-C(2)-C(3)	124.4(9)
N(6)-Ni(2)-O(6)	85.7(3)	N(3)-C(2)-C(9)	118.2(9)
N(5)-Ni(2)-O(1)#1	86.4(3)	C(3)-C(2)-C(9)	116.9(8)
N(4)-Ni(2)-O(1)#1	95.7(3)	C(4)-C(3)-C(2)	119.0(10)
O(8)-Ni(2)-O(1)#1	79.6(3)	C(4)-C(3)-H(3)	120.5
N(6)-Ni(2)-O(1)#1	84.3(3)	C(2)-C(3)-H(3)	120.5
O(6)-Ni(2)-O(1)#1	166.8(3)	C(3)-C(4)-C(5)	119.0(10)
C(9)-O(1)-Ni(2)#2	154.1(7)	C(3)-C(4)-H(4)	120.5

C(5)-C(4)-H(4)	120.5	C(14)-C(15)-H(15)	118.7
C(6)-C(5)-C(4)	125.3(10)	C(15)-C(16)-C(17)	119.9(13)
C(6)-C(5)-C(1)	116.6(10)	C(15)-C(16)-H(16)	120.1
C(4)-C(5)-C(1)	118.1(9)	C(17)-C(16)-H(16)	120.1
C(7)-C(6)-C(5)	122.0(11)	C(12)-C(17)-C(16)	117.8(12)
C(7)-C(6)-H(6)	119.0	C(12)-C(17)-C(18)	115.8(11)
C(5)-C(6)-H(6)	119.0	C(16)-C(17)-C(18)	126.4(12)
C(6)-C(7)-C(8)	117.9(10)	C(17)-C(18)-C(19)	100.5(10)
C(6)-C(7)-H(7)	121.0	C(17)-C(18)-H(18A)	111.7
C(8)-C(7)-H(7)	121.0	C(19)-C(18)-H(18A)	111.7
N(6)-C(8)-C(7)	122.8(10)	C(17)-C(18)-H(18B)	111.7
N(6)-C(8)-C(10)	117.1(10)	C(19)-C(18)-H(18B)	111.7
C(7)-C(8)-C(10)	120.1(9)	H(18A)-C(18)-H(18B)	109.4
O(1)-C(9)-N(2)	128.7(8)	O(3)-C(19)-C(11)	105.8(9)
O(1)-C(9)-C(2)	116.5(8)	O(3)-C(19)-C(18)	108.5(10)
N(2)-C(9)-C(2)	114.8(8)	C(11)-C(19)-C(18)	108.4(11)
O(2)-C(10)-N(5)	130.6(11)	O(3)-C(19)-H(19)	111.3
O(2)-C(10)-C(8)	117.2(10)	C(11)-C(19)-H(19)	111.3
N(5)-C(10)-C(8)	112.0(9)	C(18)-C(19)-H(19)	111.3
N(1)-C(11)-C(19)	104.5(8)	N(1)-C(20)-O(3)	116.7(10)
N(1)-C(11)-C(12)	117.1(9)	N(1)-C(20)-C(21)	128.5(10)
C(19)-C(11)-C(12)	105.3(10)	O(3)-C(20)-C(21)	114.7(9)
N(1)-C(11)-H(11)	109.9	C(22)-C(21)-C(26)	119.9(9)
C(19)-C(11)-H(11)	109.9	C(22)-C(21)-C(20)	115.9(10)
C(12)-C(11)-H(11)	109.9	C(26)-C(21)-C(20)	124.2(9)
C(13)-C(12)-C(17)	121.6(11)	C(23)-C(22)-C(21)	121.7(11)
C(13)-C(12)-C(11)	129.0(11)	C(23)-C(22)-H(22)	119.1
C(17)-C(12)-C(11)	108.9(10)	C(21)-C(22)-H(22)	119.1
C(12)-C(13)-C(14)	121.2(12)	C(22)-C(23)-C(24)	119.3(9)
C(12)-C(13)-H(13)	119.4	C(22)-C(23)-H(23)	120.4
C(14)-C(13)-H(13)	119.4	C(24)-C(23)-H(23)	120.4
C(15)-C(14)-C(13)	116.6(13)	C(25)-C(24)-C(23)	120.4(9)
C(15)-C(14)-H(14)	121.7	C(25)-C(24)-H(24)	119.8
C(13)-C(14)-H(14)	121.7	C(23)-C(24)-H(24)	119.8
C(16)-C(15)-C(14)	122.7(13)	C(24)-C(25)-C(26)	120.7(10)
C(16)-C(15)-H(15)	118.7	C(24)-C(25)-H(25)	119.6

C(26)-C(25)-H(25)	119.6	C(34)-C(35)-C(27)	107.7(10)
N(2)-C(26)-C(21)	120.1(8)	O(4)-C(35)-H(35)	112.0
N(2)-C(26)-C(25)	121.9(9)	C(34)-C(35)-H(35)	112.0
C(21)-C(26)-C(25)	118.0(8)	C(27)-C(35)-H(35)	112.0
N(4)-C(27)-C(28)	113.3(8)	N(4)-C(36)-O(4)	115.6(9)
N(4)-C(27)-C(35)	104.3(8)	N(4)-C(36)-C(37)	128.4(10)
C(28)-C(27)-C(35)	104.2(9)	O(4)-C(36)-C(37)	116.0(9)
N(4)-C(27)-H(27)	111.5	C(38)-C(37)-C(42)	118.5(11)
C(28)-C(27)-H(27)	111.5	C(38)-C(37)-C(36)	116.4(11)
C(35)-C(27)-H(27)	111.5	C(42)-C(37)-C(36)	125.1(10)
C(29)-C(28)-C(33)	121.8(12)	C(37)-C(38)-C(39)	124.0(14)
C(29)-C(28)-C(27)	127.4(11)	C(37)-C(38)-H(38)	118.0
C(33)-C(28)-C(27)	110.7(10)	C(39)-C(38)-H(38)	118.0
C(28)-C(29)-C(30)	115.7(13)	C(40)-C(39)-C(38)	116.6(12)
C(28)-C(29)-H(29)	122.1	C(40)-C(39)-H(39)	121.7
C(30)-C(29)-H(29)	122.1	C(38)-C(39)-H(39)	121.7
C(29)-C(30)-C(31)	120.7(14)	C(39)-C(40)-C(41)	122.4(12)
C(29)-C(30)-H(30)	119.6	C(39)-C(40)-H(40)	118.8
C(31)-C(30)-H(30)	119.6	C(41)-C(40)-H(40)	118.8
C(32)-C(31)-C(30)	118.8(15)	C(40)-C(41)-C(42)	120.9(13)
C(32)-C(31)-H(31)	120.6	C(40)-C(41)-H(41)	119.6
C(30)-C(31)-H(31)	120.6	C(42)-C(41)-H(41)	119.6
C(31)-C(32)-C(33)	120.6(15)	C(37)-C(42)-C(41)	117.5(11)
C(31)-C(32)-H(32)	119.7	C(37)-C(42)-N(5)	120.2(9)
C(33)-C(32)-H(32)	119.7	C(41)-C(42)-N(5)	122.1(11)
C(28)-C(33)-C(32)	121.1(13)	C(43)-O(5)-Ni(1)	89.4(7)
C(28)-C(33)-C(34)	110.1(11)	C(43)-O(6)-Ni(2)	130.0(7)
C(32)-C(33)-C(34)	128.7(13)	C(43)-O(6)-Ni(1)	89.9(6)
C(35)-C(34)-C(33)	104.5(10)	Ni(2)-O(6)-Ni(1)	110.2(3)
C(35)-C(34)-H(34A)	110.8	O(5)-C(43)-O(6)	118.7(10)
C(33)-C(34)-H(34A)	110.8	O(5)-C(43)-C(44)	120.0(11)
C(35)-C(34)-H(34B)	110.8	O(6)-C(43)-C(44)	121.3(9)
C(33)-C(34)-H(34B)	110.8	C(43)-C(44)-H(44A)	109.5
H(34A)-C(34)-H(34B)	108.9	C(43)-C(44)-H(44B)	109.5
O(4)-C(35)-C(34)	110.3(9)	H(44A)-C(44)-H(44B)	109.5
O(4)-C(35)-C(27)	102.5(9)	C(43)-C(44)-H(44C)	109.5

H(44A)-C(44)-H(44C)	109.5	Cl(2B)-C(52B)-H(52C)	110.6
H(44B)-C(44)-H(44C)	109.5	C(51B)-C(52B)-H(52D)	110.6
C(45)-O(7)-Ni(1)	121.7(7)	Cl(2B)-C(52B)-H(52D)	110.6
C(45)-O(8)-Ni(2)	146.2(8)	H(52C)-C(52B)-H(52D)	108.7
O(8)-C(45)-O(7)	126.5(11)	C(54A)-C(53A)-Cl(3A)	105.6(12)
O(8)-C(45)-C(46)	118.1(9)	C(54A)-C(53A)-H(53A)	110.6
O(7)-C(45)-C(46)	115.4(9)	Cl(3A)-C(53A)-H(53A)	110.6
C(45)-C(46)-H(46A)	109.5	C(54A)-C(53A)-H(53B)	110.6
C(45)-C(46)-H(46B)	109.5	Cl(3A)-C(53A)-H(53B)	110.6
H(46A)-C(46)-H(46B)	109.5	H(53A)-C(53A)-H(53B)	108.8
C(45)-C(46)-H(46C)	109.5	C(53A)-C(54A)-Cl(4A)	104.5(12)
H(46A)-C(46)-H(46C)	109.5	C(53A)-C(54A)-H(54A)	110.9
H(46B)-C(46)-H(46C)	109.5	Cl(4A)-C(54A)-H(54A)	110.9
C(52A)-C(51A)-Cl(1A)	108.2(12)	C(53A)-C(54A)-H(54B)	110.9
C(52A)-C(51A)-H(51A)	110.1	Cl(4A)-C(54A)-H(54B)	110.9
Cl(1A)-C(51A)-H(51A)	110.1	H(54A)-C(54A)-H(54B)	108.9
C(52A)-C(51A)-H(51B)	110.1	C(53B)-Cl(3B)-Cl(8A)#3	173(3)
Cl(1A)-C(51A)-H(51B)	110.1	C(54B)-C(53B)-Cl(3B)	105.0(12)
H(51A)-C(51A)-H(51B)	108.4	C(54B)-C(53B)-H(53C)	110.7
C(51A)-C(52A)-Cl(2A)	106.1(12)	Cl(3B)-C(53B)-H(53C)	110.7
C(51A)-C(52A)-H(52A)	110.5	C(54B)-C(53B)-H(53D)	110.7
Cl(2A)-C(52A)-H(52A)	110.5	Cl(3B)-C(53B)-H(53D)	110.7
C(51A)-C(52A)-H(52B)	110.5	H(53C)-C(53B)-H(53D)	108.8
Cl(2A)-C(52A)-H(52B)	110.5	C(53B)-C(54B)-Cl(4B)	105.2(12)
H(52A)-C(52A)-H(52B)	108.7	C(53B)-C(54B)-H(54C)	110.7
C(52B)-C(51B)-Cl(1B)	105.7(12)	Cl(4B)-C(54B)-H(54C)	110.7
C(52B)-C(51B)-H(51C)	110.6	C(53B)-C(54B)-H(54D)	110.7
Cl(1B)-C(51B)-H(51C)	110.6	Cl(4B)-C(54B)-H(54D)	110.7
C(52B)-C(51B)-H(51D)	110.6	H(54C)-C(54B)-H(54D)	108.8
Cl(1B)-C(51B)-H(51D)	110.6	C(58A)-C(57A)-Cl(7A)	104.8(12)
H(51C)-C(51B)-H(51D)	108.7	C(57A)-C(58A)-Cl(8A)	105.8(12)
C(51B)-C(52B)-Cl(2B)	105.6(12)	C(58A)-Cl(8A)-Cl(3B)#4	157(3)
C(51B)-C(52B)-H(52C)	110.6		

Symmetry transformations used to generate equivalent atoms:

#1 -x+1,y+1/2,-z+1/2 #2 -x+1,y-1/2,-z+1/2 #3 -x+3/2,-y+1,z+1/2 #4 -x+3/2,-y+1,z-1/2

Table S4. Anisotropic displacement parameters ($Å^2$x 10^3) for **16•Ni2OAc2**. The anisotropic displacement factor exponent takes the form: $-2\pi^2[h^2 a^{*2}U^{11} + ... + 2 h k a^* b^* U^{12}]$

U^{11}	U^{22}	U^{33}	U^{23}	U^{13}	U^{12}	
Ni(1)	51(1)	25(1)	59(1)	1(1)	1(1)	6(1)
Ni(2)	47(1)	22(1)	67(1)	0(1)	2(1)	-2(1)
O(1)	59(4)	31(3)	68(4)	-3(3)	7(3)	5(3)
O(2)	69(5)	39(4)	107(6)	2(4)	-2(4)	-20(3)
O(3)	69(4)	37(3)	84(4)	-7(3)	18(4)	-5(3)
O(4)	72(4)	53(4)	54(4)	-6(3)	4(3)	-4(3)
N(1)	59(4)	29(3)	59(4)	0(3)	2(3)	9(3)
N(2)	61(4)	27(3)	59(4)	0(3)	1(3)	-1(3)
N(3)	52(4)	26(3)	65(4)	-3(3)	2(3)	-1(3)
N(4)	54(4)	30(3)	62(4)	-1(3)	1(3)	0(3)
N(5)	53(4)	33(3)	85(5)	-1(3)	-5(3)	-2(3)
N(6)	51(4)	31(3)	83(5)	2(3)	6(3)	-3(3)
C(1)	45(4)	30(3)	70(5)	3(3)	6(4)	-4(3)
C(2)	50(4)	27(3)	65(4)	-3(3)	4(4)	2(3)
C(3)	65(5)	36(4)	68(5)	2(4)	9(4)	3(4)
C(4)	60(5)	38(4)	77(5)	9(4)	17(4)	4(4)
C(5)	56(5)	41(4)	83(5)	8(4)	18(4)	7(4)
C(6)	55(5)	45(4)	85(6)	8(4)	12(4)	5(4)
C(7)	54(5)	37(4)	89(6)	5(4)	10(4)	2(4)
C(8)	52(4)	34(4)	90(5)	1(4)	3(4)	-2(3)
C(9)	51(4)	29(3)	60(4)	-1(3)	2(4)	0(3)
C(10)	57(4)	32(4)	91(5)	-2(4)	0(4)	-2(3)
C(11)	59(4)	37(4)	67(4)	-2(3)	3(4)	7(3)
C(12)	62(5)	50(4)	60(4)	-1(4)	4(4)	14(4)
C(13)	59(5)	54(5)	71(5)	-4(4)	4(4)	9(4)
C(14)	63(5)	73(5)	71(5)	-7(4)	-3(5)	14(5)
C(15)	78(6)	87(6)	65(5)	-16(5)	-2(5)	17(5)
C(16)	84(6)	74(5)	69(5)	1(5)	9(5)	18(5)
C(17)	79(5)	51(4)	70(4)	0(4)	10(4)	13(4)
C(18)	93(6)	56(5)	86(5)	4(5)	17(5)	2(5)
C(19)	72(5)	45(4)	82(5)	1(4)	12(4)	8(4)

C(20)	64(4)	39(4)	59(4)	-3(4)	5(4)	-3(4)
C(21)	68(5)	36(4)	63(4)	0(3)	2(4)	5(4)
C(22)	83(6)	44(4)	59(5)	5(4)	9(4)	5(4)
C(23)	81(6)	35(4)	70(5)	13(4)	5(5)	7(4)
C(24)	75(5)	31(4)	72(5)	5(4)	-3(4)	11(4)
C(25)	60(5)	30(3)	65(5)	0(3)	-2(4)	2(3)
C(26)	58(4)	27(3)	64(4)	3(3)	4(4)	1(3)
C(27)	53(4)	41(4)	58(4)	0(3)	4(3)	-5(3)
C(28)	60(4)	50(4)	65(4)	3(4)	0(4)	1(4)
C(29)	74(6)	72(6)	66(5)	10(5)	-1(5)	6(5)
C(30)	91(6)	99(7)	86(6)	23(6)	-6(5)	19(6)
C(31)	89(7)	88(7)	105(6)	16(6)	-7(6)	25(6)
C(32)	77(6)	87(6)	99(6)	4(6)	-4(6)	22(5)
C(33)	61(5)	68(5)	81(5)	0(4)	1(4)	9(4)
C(34)	68(5)	64(5)	74(5)	-6(5)	9(4)	0(4)
C(35)	64(4)	51(4)	64(4)	1(4)	4(4)	-5(4)
C(36)	62(4)	36(4)	59(4)	1(3)	-4(4)	2(3)
C(37)	71(4)	33(4)	68(5)	1(3)	-11(4)	-2(4)
C(38)	87(6)	51(5)	74(5)	0(4)	-11(5)	-4(5)
C(39)	98(6)	48(5)	78(6)	-8(5)	-18(5)	-2(5)
C(40)	85(6)	36(4)	92(6)	-8(4)	-27(5)	-4(4)
C(41)	77(5)	30(4)	94(6)	-4(4)	-15(5)	-3(4)
C(42)	66(4)	23(3)	78(5)	3(3)	-11(4)	0(3)
O(5)	68(4)	37(3)	82(4)	-4(3)	-10(4)	11(3)
O(6)	63(4)	27(3)	64(4)	0(2)	-6(3)	5(3)
C(43)	67(5)	29(4)	69(5)	-2(3)	-10(4)	1(3)
C(44)	78(7)	56(6)	63(6)	-7(5)	-10(5)	4(5)
O(7)	57(3)	32(3)	70(4)	-1(3)	-2(3)	-4(3)
O(8)	53(3)	43(3)	65(4)	1(3)	2(3)	-2(3)
C(45)	60(4)	33(4)	63(5)	6(4)	14(4)	9(3)
C(46)	65(6)	45(6)	96(8)	-3(5)	-10(6)	1(5)

Table S5. Hydrogen coordinates (x 10^4) and isotropic displacement parameters ($Å^2$x 10^3) for **16•Ni2OAc2**.

x	y	z	U(eq)	
H(3)	6414	3879	1767	67
H(4)	7109	5252	1534	70
H(6)	7370	6906	1789	74
H(7)	7100	8067	2366	72
H(11)	2841	4878	4030	65
H(13)	3924	6125	4585	74
H(14)	4096	6707	5462	83
H(15)	3459	5857	6164	92
H(16)	2830	4468	6031	91
H(18A)	1832	3592	5182	94
H(18B)	2831	3069	5178	94
H(19)	1887	3772	4268	80
H(22)	3932	1603	4386	74
H(23)	4838	363	4155	74
H(24)	5978	498	3480	71
H(25)	6127	1839	3008	62
H(27)	2311	6465	3663	61
H(29)	2512	7449	2633	85
H(30)	1461	8418	2166	110
H(31)	522	9489	2668	113
H(32)	181	9153	3516	105
H(34A)	1106	8450	4411	82
H(34B)	570	7534	4251	82
H(35)	1902	6865	4509	72
H(38)	3753	8686	5040	85
H(39)	5011	9476	5415	90
H(40)	6439	9426	4985	85
H(41)	6576	8837	4148	80
H(44A)	5853	6853	4377	98
H(44B)	6815	6328	4257	98

H(44C)	6168	6031	4747	98
H(46A)	1837	5240	2675	103
H(46B)	2465	4700	2255	103
H(46C)	2377	5771	2214	103
H(51A)	-3074	3409	3609	111
H(51B)	-2934	3093	4213	111
H(52A)	-2289	4579	3986	215
H(52B)	-1472	4039	3679	215
H(51C)	-1493	4862	3445	252
H(51D)	-1150	5090	4037	252
H(52C)	-421	3658	3402	276
H(52D)	-97	3847	4003	276
H(53A)	5322	4134	4716	237
H(53B)	5332	4360	5340	237
H(54A)	6856	4003	4715	239
H(54B)	6803	3259	5178	239
H(53C)	5269	3589	5452	251
H(53D)	5558	4616	5575	251
H(54C)	6715	3487	5126	235
H(54D)	7021	3388	5737	235

Table S6. Torsion angles [°] for **16•Ni2OAc2**.

Symmetry transformations used to generate equivalent atoms:

#1 -x+1,y+1/2,-z+1/2 #2 -x+1,y-1/2,-z+1/2 #3 -x+3/2,-y+1,z+1/2 #4 -x+3/2,-y+1,z-1/2

C(8)-N(6)-C(1)-N(3)	169.4(10)	C(26)-N(2)-C(9)-O(1)	10.7(16)
Ni(2)-N(6)-C(1)-N(3)	-37.8(14)	Ni(1)-N(2)-C(9)-O(1)	-169.4(9)
C(8)-N(6)-C(1)-C(5)	-7.0(16)	C(26)-N(2)-C(9)-C(2)	-169.5(9)
Ni(2)-N(6)-C(1)-C(5)	145.8(9)	Ni(1)-N(2)-C(9)-C(2)	10.4(11)
C(2)-N(3)-C(1)-N(6)	179.8(9)	N(3)-C(2)-C(9)-O(1)	178.1(9)
Ni(1)-N(3)-C(1)-N(6)	-5.2(15)	C(3)-C(2)-C(9)-O(1)	-9.5(14)
C(2)-N(3)-C(1)-C(5)	-3.9(16)	N(3)-C(2)-C(9)-N(2)	-1.7(13)
Ni(1)-N(3)-C(1)-C(5)	171.1(8)	C(3)-C(2)-C(9)-N(2)	170.7(9)
C(1)-N(3)-C(2)-C(3)	-2.9(16)	C(42)-N(5)-C(10)-O(2)	-11.2(18)
Ni(1)-N(3)-C(2)-C(3)	-179.0(9)	Ni(2)-N(5)-C(10)-O(2)	167.3(10)
C(1)-N(3)-C(2)-C(9)	169.0(9)	C(42)-N(5)-C(10)-C(8)	163.5(9)
Ni(1)-N(3)-C(2)-C(9)	-7.1(11)	Ni(2)-N(5)-C(10)-C(8)	-18.0(11)
N(3)-C(2)-C(3)-C(4)	6.0(17)	N(6)-C(8)-C(10)-O(2)	165.7(10)
C(9)-C(2)-C(3)-C(4)	-166.0(10)	C(7)-C(8)-C(10)-O(2)	-11.9(16)
C(2)-C(3)-C(4)-C(5)	-2.1(17)	N(6)-C(8)-C(10)-N(5)	-9.8(14)
C(3)-C(4)-C(5)-C(6)	176.9(12)	C(7)-C(8)-C(10)-N(5)	172.6(10)
C(3)-C(4)-C(5)-C(1)	-4.1(17)	C(20)-N(1)-C(11)-C(19)	-0.2(12)
N(6)-C(1)-C(5)-C(6)	2.6(17)	Ni(1)-N(1)-C(11)-C(19)	149.7(8)
N(3)-C(1)-C(5)-C(6)	-173.6(10)	C(20)-N(1)-C(11)-C(12)	115.7(10)
N(6)-C(1)-C(5)-C(4)	-176.5(11)	Ni(1)-N(1)-C(11)-C(12)	-94.3(10)
N(3)-C(1)-C(5)-C(4)	7.3(17)	N(1)-C(11)-C(12)-C(13)	79.2(14)
C(4)-C(5)-C(6)-C(7)	-178.7(12)	C(19)-C(11)-C(12)-C(13)	-165.2(12)
C(1)-C(5)-C(6)-C(7)	2.3(18)	N(1)-C(11)-C(12)-C(17)	-108.4(11)
C(5)-C(6)-C(7)-C(8)	-2.7(18)	C(19)-C(11)-C(12)-C(17)	7.2(12)
C(1)-N(6)-C(8)-C(7)	6.7(16)	C(17)-C(12)-C(13)-C(14)	5.5(18)
Ni(2)-N(6)-C(8)-C(7)	-152.1(9)	C(11)-C(12)-C(13)-C(14)	177.1(11)
C(1)-N(6)-C(8)-C(10)	-170.9(9)	C(12)-C(13)-C(14)-C(15)	-4.0(17)
Ni(2)-N(6)-C(8)-C(10)	30.3(11)	C(13)-C(14)-C(15)-C(16)	2(2)
C(6)-C(7)-C(8)-N(6)	-1.8(18)	C(14)-C(15)-C(16)-C(17)	-2(2)
C(6)-C(7)-C(8)-C(10)	175.7(10)	C(13)-C(12)-C(17)-C(16)	-5.1(18)
Ni(2)#2-O(1)-C(9)-N(2)	102.6(16)	C(11)-C(12)-C(17)-C(16)	-178.2(11)
Ni(2)#2-O(1)-C(9)-C(2)	-77.2(17)	C(13)-C(12)-C(17)-C(18)	172.3(11)

C(11)-C(12)-C(17)-C(18)	-0.8(15)	C(24)-C(25)-C(26)-N(2)	178.5(10)
C(15)-C(16)-C(17)-C(12)	3(2)	C(24)-C(25)-C(26)-C(21)	1.0(16)
C(15)-C(16)-C(17)-C(18)	-173.6(13)	C(36)-N(4)-C(27)-C(28)	100.2(10)
C(12)-C(17)-C(18)-C(19)	-5.5(15)	Ni(2)-N(4)-C(27)-C(28)	-87.8(10)
C(16)-C(17)-C(18)-C(19)	171.6(13)	C(36)-N(4)-C(27)-C(35)	-12.5(10)
C(20)-O(3)-C(19)-C(11)	4.5(12)	Ni(2)-N(4)-C(27)-C(35)	159.5(7)
C(20)-O(3)-C(19)-C(18)	-111.5(11)	N(4)-C(27)-C(28)-C(29)	58.4(15)
N(1)-C(11)-C(19)-O(3)	-2.7(12)	C(35)-C(27)-C(28)-C(29)	171.2(12)
C(12)-C(11)-C(19)-O(3)	-126.6(10)	N(4)-C(27)-C(28)-C(33)	-117.6(11)
N(1)-C(11)-C(19)-C(18)	113.4(10)	C(35)-C(27)-C(28)-C(33)	-4.8(12)
C(12)-C(11)-C(19)-C(18)	-10.5(12)	C(33)-C(28)-C(29)-C(30)	-1(2)
C(17)-C(18)-C(19)-O(3)	124.1(11)	C(27)-C(28)-C(29)-C(30)	-176.8(12)
C(17)-C(18)-C(19)-C(11)	9.7(13)	C(28)-C(29)-C(30)-C(31)	10(2)
C(11)-N(1)-C(20)-O(3)	3.5(13)	C(29)-C(30)-C(31)-C(32)	-14(3)
Ni(1)-N(1)-C(20)-O(3)	-149.2(8)	C(30)-C(31)-C(32)-C(33)	9(3)
C(11)-N(1)-C(20)-C(21)	-174.2(11)	C(29)-C(28)-C(33)-C(32)	-3(2)
Ni(1)-N(1)-C(20)-C(21)	33.1(16)	C(27)-C(28)-C(33)-C(32)	172.9(12)
C(19)-O(3)-C(20)-N(1)	-5.3(14)	C(29)-C(28)-C(33)-C(34)	177.9(12)
C(19)-O(3)-C(20)-C(21)	172.7(10)	C(27)-C(28)-C(33)-C(34)	-5.9(14)
N(1)-C(20)-C(21)-C(22)	148.2(12)	C(31)-C(32)-C(33)-C(28)	-1(3)
O(3)-C(20)-C(21)-C(22)	-29.5(15)	C(31)-C(32)-C(33)-C(34)	177.6(16)
N(1)-C(20)-C(21)-C(26)	-34.3(19)	C(28)-C(33)-C(34)-C(35)	14.4(15)
O(3)-C(20)-C(21)-C(26)	148.0(10)	C(32)-C(33)-C(34)-C(35)	-164.2(14)
C(26)-C(21)-C(22)-C(23)	1.0(19)	C(36)-O(4)-C(35)-C(34)	-129.6(10)
C(20)-C(21)-C(22)-C(23)	178.6(11)	C(36)-O(4)-C(35)-C(27)	-15.2(11)
C(21)-C(22)-C(23)-C(24)	1.2(19)	C(33)-C(34)-C(35)-O(4)	94.1(12)
C(22)-C(23)-C(24)-C(25)	-2.3(18)	C(33)-C(34)-C(35)-C(27)	-16.9(13)
C(23)-C(24)-C(25)-C(26)	1.2(17)	N(4)-C(27)-C(35)-O(4)	16.5(10)
C(9)-N(2)-C(26)-C(21)	-159.8(10)	C(28)-C(27)-C(35)-O(4)	-102.5(9)
Ni(1)-N(2)-C(26)-C(21)	20.3(14)	N(4)-C(27)-C(35)-C(34)	132.8(9)
C(9)-N(2)-C(26)-C(25)	22.7(15)	C(28)-C(27)-C(35)-C(34)	13.8(12)
Ni(1)-N(2)-C(26)-C(25)	-157.2(8)	C(27)-N(4)-C(36)-O(4)	3.1(12)
C(22)-C(21)-C(26)-N(2)	-179.6(10)	Ni(2)-N(4)-C(36)-O(4)	-169.5(6)
C(20)-C(21)-C(26)-N(2)	2.9(16)	C(27)-N(4)-C(36)-C(37)	-175.8(10)
C(22)-C(21)-C(26)-C(25)	-2.0(16)	Ni(2)-N(4)-C(36)-C(37)	11.6(14)
C(20)-C(21)-C(26)-C(25)	-179.5(10)	C(35)-O(4)-C(36)-N(4)	8.4(12)

C(35)-O(4)-C(36)-C(37)	-172.6(9)
N(4)-C(36)-C(37)-C(38)	-173.2(10)
O(4)-C(36)-C(37)-C(38)	7.9(14)
N(4)-C(36)-C(37)-C(42)	6.7(17)
O(4)-C(36)-C(37)-C(42)	-172.2(9)
C(42)-C(37)-C(38)-C(39)	0.1(17)
C(36)-C(37)-C(38)-C(39)	-180.0(11)
C(37)-C(38)-C(39)-C(40)	-3.3(19)
C(38)-C(39)-C(40)-C(41)	5.3(19)
C(39)-C(40)-C(41)-C(42)	-4.1(18)
C(38)-C(37)-C(42)-C(41)	1.3(15)
C(36)-C(37)-C(42)-C(41)	-178.7(9)
C(38)-C(37)-C(42)-N(5)	175.5(10)
C(36)-C(37)-C(42)-N(5)	-4.5(15)
C(40)-C(41)-C(42)-C(37)	0.6(15)
C(40)-C(41)-C(42)-N(5)	-173.5(10)
C(10)-N(5)-C(42)-C(37)	162.3(9)
Ni(2)-N(5)-C(42)-C(37)	-16.1(13)
C(10)-N(5)-C(42)-C(41)	-23.8(15)
Ni(2)-N(5)-C(42)-C(41)	157.9(8)
Ni(1)-O(5)-C(43)-O(6)	3.2(11)
Ni(1)-O(5)-C(43)-C(44)	-178.1(10)
Ni(2)-O(6)-C(43)-O(5)	-119.8(9)
Ni(1)-O(6)-C(43)-O(5)	-3.2(11)
Ni(2)-O(6)-C(43)-C(44)	61.5(15)
Ni(1)-O(6)-C(43)-C(44)	178.1(10)
Ni(2)-O(8)-C(45)-O(7)	-9(2)
Ni(2)-O(8)-C(45)-C(46)	171.0(9)
Ni(1)-O(7)-C(45)-O(8)	-7.0(15)
Ni(1)-O(7)-C(45)-C(46)	173.3(7)
Cl(1A)-C(51A)-C(52A)-Cl(2A)	-77(2)
Cl(1B)-C(51B)-C(52B)-Cl(2B)	-178(3)
Cl(3A)-C(53A)-C(54A)-Cl(4A)	-124(2)
Cl(3B)-C(53B)-C(54B)-Cl(4B)	-100(3)
Cl(7A)-C(57A)-C(58A)-Cl(8A)	-167(3)
C(57A)-C(58A)-Cl(8A)-Cl(3B)#4	122(5)

Figure S2. ORTEP diagram of **25•Ni2OAc** with H atoms and solvate atoms removed and ellipsoids drawn at the 30% level.

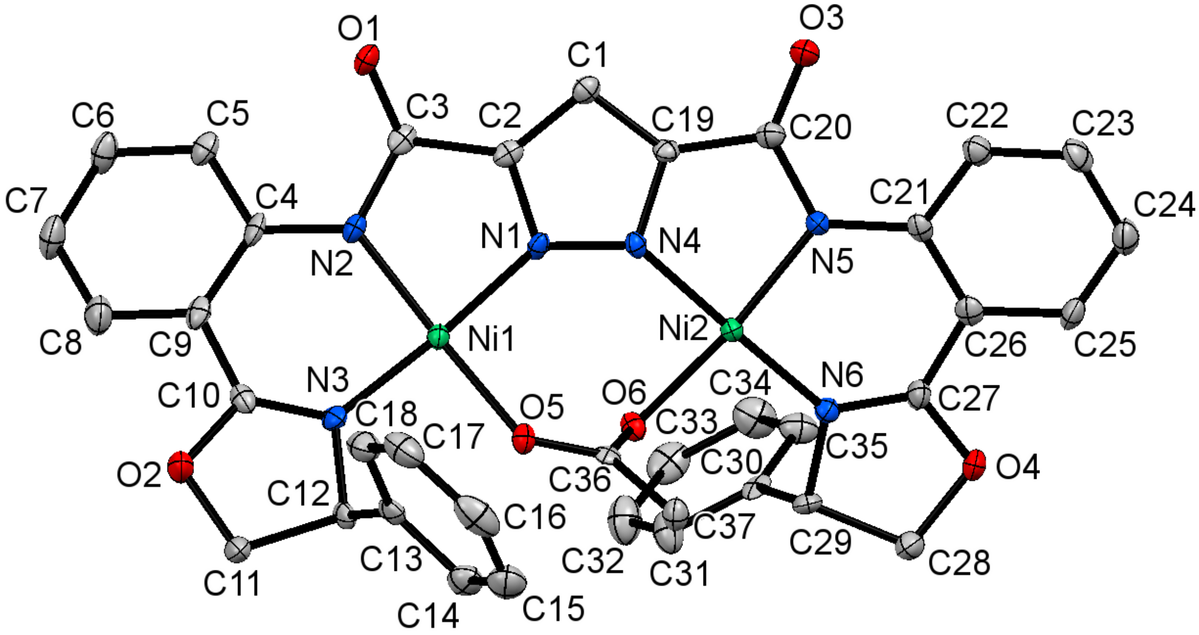

Table S7. Crystal data and structure refinement for **25•Ni2OAc**.

Identification code	25-Ni2OAc	
Empirical formula	C38 H30 Cl2 N6 Ni2 O6	
Formula weight	855.00	
Temperature	100(2) K	
Wavelength	0.71073 Å	
Crystal system	Monoclinic	
Space group	$P2_1$	
Unit cell dimensions	a = 9.0473(9) Å	α= 90°.
b = 17.5433(17) Å	β= 109.643(2)°.	
c = 11.7671(11) Å	γ = 90°.	
Volume	1759.0(3) $Å^3$	
Z	2	
Density (calculated)	1.614 Mg/m^3	
Absorption coefficient	1.281 mm^{-1}	
F(000)	876	
Crystal size	0.250 x 0.040 x 0.020 mm^3	
Theta range for data collection	1.838 to 28.333°.	
Index ranges	-12<=h<=12, -21<=k<=23, -15<=l<=13	
Reflections collected	12601	
Independent reflections	7091 [R(int) = 0.0627]	
Completeness to theta = 25.242°	100.0 %	
Absorption correction	Semi-empirical from equivalents	
Max. and min. transmission	.9999 and 0.7329	
Refinement method	Full-matrix least-squares on F^2	
Data / restraints / parameters	7091 / 874 / 488	
Goodness-of-fit on F^2	1.011	
Final R indices [I>2sigma(I)]	R1 = 0.0607, wR2 = 0.1234	
R indices (all data)	R1 = 0.0861, wR2 = 0.1355	
Absolute structure parameter	-0.02(2)	
Extinction coefficient	n/a	
Largest diff. peak and hole	0.902 and -0.637 e.$Å^{-3}$	

Table S8. Atomic coordinates (x 10^4) and equivalent isotropic displacement parameters ($Å^2$x 10^3) for **25•Ni2OAc**. U(eq) is defined as one third of the trace of the orthogonalized U^{ij} tensor.

x	y	z	U(eq)	
Ni(1)	852(1)	-267(1)	3605(1)	12(1)
Ni(2)	-706(1)	1301(1)	782(1)	11(1)
O(1)	-3062(6)	-871(3)	4111(5)	19(1)
O(2)	4049(6)	-1629(4)	5957(5)	23(1)
O(3)	-5069(6)	1746(3)	559(5)	21(1)
O(4)	19(6)	2541(3)	-1858(5)	20(1)
N(1)	-1016(7)	221(4)	2642(6)	12(1)
N(2)	-545(7)	-952(4)	3983(6)	14(1)
N(3)	2637(7)	-736(4)	4709(6)	14(1)
N(4)	-1443(7)	824(4)	1894(6)	12(1)
N(5)	-2570(7)	1890(4)	398(6)	13(1)
N(6)	-80(7)	1726(4)	-435(6)	13(1)
C(1)	-3534(9)	483(5)	2391(7)	15(2)
C(2)	-2257(8)	22(4)	2959(7)	13(1)
C(3)	-2036(9)	-648(5)	3749(7)	14(2)
C(4)	-212(9)	-1674(5)	4490(7)	16(2)
C(5)	-1406(10)	-2215(5)	4421(7)	18(2)
C(6)	-1035(10)	-2950(5)	4913(8)	23(2)
C(7)	526(11)	-3157(5)	5479(8)	26(2)
C(8)	1695(11)	-2644(5)	5570(8)	26(2)
C(9)	1353(9)	-1906(5)	5096(7)	17(2)
C(10)	2650(9)	-1390(5)	5216(7)	15(2)
C(11)	5214(9)	-1068(5)	5910(8)	19(2)
C(12)	4260(8)	-416(5)	5111(7)	15(2)
C(13)	4417(8)	314(5)	5792(7)	17(2)
C(14)	5267(9)	925(5)	5527(8)	22(2)
C(15)	5507(10)	1592(6)	6186(9)	28(2)
C(16)	4912(10)	1667(6)	7114(9)	32(2)
C(17)	4111(10)	1076(6)	7414(8)	29(2)
C(18)	3836(9)	411(6)	6734(8)	23(2)
C(19)	-2971(8)	984(4)	1718(7)	13(1)

C(20)	-3667(9)	1586(5)	843(7)	17(2)
C(21)	-2892(8)	2560(5)	-300(7)	14(2)
C(22)	-4126(9)	3059(5)	-300(7)	18(2)
C(23)	-4445(10)	3704(5)	-1011(8)	22(2)
C(24)	-3558(10)	3894(5)	-1732(8)	22(2)
C(25)	-2332(9)	3445(5)	-1715(7)	19(2)
C(26)	-1975(9)	2774(5)	-1002(7)	15(2)
C(27)	-671(9)	2312(5)	-1069(7)	16(2)
C(28)	1385(9)	2058(5)	-1669(8)	20(2)
C(29)	1192(8)	1418(5)	-843(7)	15(1)
C(30)	748(9)	661(5)	-1485(7)	15(2)
C(31)	1686(11)	19(5)	-1103(8)	26(2)
C(32)	1300(12)	-662(6)	-1737(9)	33(2)
C(33)	-17(11)	-710(6)	-2756(8)	30(2)
C(34)	-959(10)	-88(6)	-3147(9)	31(2)
C(35)	-591(9)	599(6)	-2505(8)	24(2)
O(5)	2197(6)	482(3)	3350(5)	15(1)
O(6)	1109(6)	679(3)	1382(5)	15(1)
C(36)	1983(8)	886(5)	2418(7)	12(2)
C(37)	2863(9)	1628(5)	2582(7)	18(2)
C(38)	1635(10)	4230(5)	163(9)	28(2)
Cl(1)	3145(3)	4891(1)	769(2)	33(1)
Cl(2)	2155(3)	3329(1)	835(2)	34(1)

Table S9. Bond lengths [Å] and angles [°] for **25•Ni2OAc**.

Ni(1)-O(5)	1.881(5)	C(6)-H(6)	0.9500
Ni(1)-N(3)	1.887(6)	C(7)-C(8)	1.366(12)
Ni(1)-N(1)	1.894(6)	C(7)-H(7)	0.9500
Ni(1)-N(2)	1.903(6)	C(8)-C(9)	1.402(12)
Ni(2)-N(4)	1.855(7)	C(8)-H(8)	0.9500
Ni(2)-N(6)	1.864(6)	C(9)-C(10)	1.451(11)
Ni(2)-N(5)	1.898(6)	C(11)-C(12)	1.546(11)
Ni(2)-O(6)	1.899(5)	C(11)-H(11A)	0.9900
O(1)-C(3)	1.210(9)	C(11)-H(11B)	0.9900
O(2)-C(10)	1.340(9)	C(12)-C(13)	1.492(12)
O(2)-C(11)	1.457(10)	C(12)-H(12)	1.0000
O(3)-C(20)	1.231(9)	C(13)-C(18)	1.389(12)
O(4)-C(27)	1.342(9)	C(13)-C(14)	1.413(12)
O(4)-C(28)	1.453(9)	C(14)-C(15)	1.380(13)
N(1)-C(2)	1.343(10)	C(14)-H(14)	0.9500
N(1)-N(4)	1.347(9)	C(15)-C(16)	1.376(13)
N(2)-C(4)	1.389(10)	C(15)-H(15)	0.9500
N(2)-C(3)	1.389(10)	C(16)-C(17)	1.377(14)
N(3)-C(10)	1.292(10)	C(16)-H(16)	0.9500
N(3)-C(12)	1.492(9)	C(17)-C(18)	1.388(13)
N(4)-C(19)	1.357(9)	C(17)-H(17)	0.9500
N(5)-C(20)	1.376(10)	C(18)-H(18)	0.9500
N(5)-C(21)	1.407(10)	C(19)-C(20)	1.461(11)
N(6)-C(27)	1.278(10)	C(21)-C(26)	1.404(11)
N(6)-C(29)	1.489(9)	C(21)-C(22)	1.420(11)
C(1)-C(2)	1.384(11)	C(22)-C(23)	1.379(12)
C(1)-C(19)	1.389(11)	C(22)-H(22)	0.9500
C(1)-H(1)	0.9500	C(23)-C(24)	1.390(13)
C(2)-C(3)	1.470(11)	C(23)-H(23)	0.9500
C(4)-C(9)	1.414(11)	C(24)-C(25)	1.355(12)
C(4)-C(5)	1.421(11)	C(24)-H(24)	0.9500
C(5)-C(6)	1.406(12)	C(25)-C(26)	1.418(11)
C(5)-H(5)	0.9500	C(25)-H(25)	0.9500
C(6)-C(7)	1.392(12)	C(26)-C(27)	1.456(11)

C(28)-C(29)	1.534(11)	C(34)-C(35)	1.402(13)
C(28)-H(28A)	0.9900	C(34)-H(34)	0.9500
C(28)-H(28B)	0.9900	C(35)-H(35)	0.9500
C(29)-C(30)	1.514(12)	O(5)-C(36)	1.266(9)
C(29)-H(29)	1.0000	O(6)-C(36)	1.263(9)
C(30)-C(31)	1.391(12)	C(36)-C(37)	1.503(11)
C(30)-C(35)	1.394(11)	C(37)-H(37A)	0.9800
C(31)-C(32)	1.389(13)	C(37)-H(37B)	0.9800
C(31)-H(31)	0.9500	C(37)-H(37C)	0.9800
C(32)-C(33)	1.380(13)	C(38)-Cl(1)	1.751(9)
C(32)-H(32)	0.9500	C(38)-Cl(2)	1.759(9)
C(33)-C(34)	1.365(13)	C(38)-H(38A)	0.9900
C(33)-H(33)	0.9500	C(38)-H(38B)	0.9900

O(5)-Ni(1)-N(3)	88.0(3)	C(12)-N(3)-Ni(1)	125.3(5)
O(5)-Ni(1)-N(1)	94.8(3)	N(1)-N(4)-C(19)	108.4(6)
N(3)-Ni(1)-N(1)	173.7(3)	N(1)-N(4)-Ni(2)	136.3(5)
O(5)-Ni(1)-N(2)	174.0(3)	C(19)-N(4)-Ni(2)	112.9(5)
N(3)-Ni(1)-N(2)	92.6(3)	C(20)-N(5)-C(21)	120.8(6)
N(1)-Ni(1)-N(2)	84.0(3)	C(20)-N(5)-Ni(2)	113.3(5)
N(4)-Ni(2)-N(6)	175.1(3)	C(21)-N(5)-Ni(2)	125.9(5)
N(4)-Ni(2)-N(5)	85.0(3)	C(27)-N(6)-C(29)	108.7(7)
N(6)-Ni(2)-N(5)	94.3(3)	C(27)-N(6)-Ni(2)	126.5(6)
N(4)-Ni(2)-O(6)	87.1(3)	C(29)-N(6)-Ni(2)	124.8(5)
N(6)-Ni(2)-O(6)	93.9(2)	C(2)-C(1)-C(19)	103.8(7)
N(5)-Ni(2)-O(6)	171.6(3)	C(2)-C(1)-H(1)	128.1
C(10)-O(2)-C(11)	107.5(6)	C(19)-C(1)-H(1)	128.1
C(27)-O(4)-C(28)	106.8(6)	N(1)-C(2)-C(1)	110.7(7)
C(2)-N(1)-N(4)	107.6(6)	N(1)-C(2)-C(3)	115.8(7)
C(2)-N(1)-Ni(1)	112.2(5)	C(1)-C(2)-C(3)	133.4(7)
N(4)-N(1)-Ni(1)	138.4(5)	O(1)-C(3)-N(2)	127.9(8)
C(4)-N(2)-C(3)	119.5(6)	O(1)-C(3)-C(2)	121.7(7)
C(4)-N(2)-Ni(1)	127.6(5)	N(2)-C(3)-C(2)	110.5(6)
C(3)-N(2)-Ni(1)	112.8(5)	N(2)-C(4)-C(9)	121.0(7)
C(10)-N(3)-C(12)	109.4(6)	N(2)-C(4)-C(5)	122.3(7)
C(10)-N(3)-Ni(1)	125.3(5)	C(9)-C(4)-C(5)	116.7(7)

C(6)-C(5)-C(4)	121.1(8)	C(16)-C(15)-C(14)	119.8(9)
C(6)-C(5)-H(5)	119.4	C(16)-C(15)-H(15)	120.1
C(4)-C(5)-H(5)	119.4	C(14)-C(15)-H(15)	120.1
C(7)-C(6)-C(5)	120.0(8)	C(15)-C(16)-C(17)	120.8(9)
C(7)-C(6)-H(6)	120.0	C(15)-C(16)-H(16)	119.6
C(5)-C(6)-H(6)	120.0	C(17)-C(16)-H(16)	119.6
C(8)-C(7)-C(6)	119.9(8)	C(16)-C(17)-C(18)	119.5(9)
C(8)-C(7)-H(7)	120.0	C(16)-C(17)-H(17)	120.2
C(6)-C(7)-H(7)	120.0	C(18)-C(17)-H(17)	120.2
C(7)-C(8)-C(9)	121.1(8)	C(17)-C(18)-C(13)	121.4(9)
C(7)-C(8)-H(8)	119.5	C(17)-C(18)-H(18)	119.3
C(9)-C(8)-H(8)	119.5	C(13)-C(18)-H(18)	119.3
C(8)-C(9)-C(4)	121.1(8)	N(4)-C(19)-C(1)	109.4(7)
C(8)-C(9)-C(10)	118.3(7)	N(4)-C(19)-C(20)	115.6(7)
C(4)-C(9)-C(10)	120.6(7)	C(1)-C(19)-C(20)	134.8(7)
N(3)-C(10)-O(2)	116.1(7)	O(3)-C(20)-N(5)	128.1(8)
N(3)-C(10)-C(9)	128.9(7)	O(3)-C(20)-C(19)	121.2(7)
O(2)-C(10)-C(9)	115.0(7)	N(5)-C(20)-C(19)	110.7(6)
O(2)-C(11)-C(12)	105.1(6)	C(26)-C(21)-N(5)	121.3(7)
O(2)-C(11)-H(11A)	110.7	C(26)-C(21)-C(22)	117.1(7)
C(12)-C(11)-H(11A)	110.7	N(5)-C(21)-C(22)	121.6(7)
O(2)-C(11)-H(11B)	110.7	C(23)-C(22)-C(21)	120.7(8)
C(12)-C(11)-H(11B)	110.7	C(23)-C(22)-H(22)	119.7
H(11A)-C(11)-H(11B)	108.8	C(21)-C(22)-H(22)	119.7
C(13)-C(12)-N(3)	113.5(6)	C(22)-C(23)-C(24)	121.4(8)
C(13)-C(12)-C(11)	112.1(6)	C(22)-C(23)-H(23)	119.3
N(3)-C(12)-C(11)	101.8(6)	C(24)-C(23)-H(23)	119.3
C(13)-C(12)-H(12)	109.8	C(25)-C(24)-C(23)	119.3(8)
N(3)-C(12)-H(12)	109.8	C(25)-C(24)-H(24)	120.4
C(11)-C(12)-H(12)	109.8	C(23)-C(24)-H(24)	120.4
C(18)-C(13)-C(14)	117.6(8)	C(24)-C(25)-C(26)	120.9(8)
C(18)-C(13)-C(12)	122.8(8)	C(24)-C(25)-H(25)	119.5
C(14)-C(13)-C(12)	119.6(7)	C(26)-C(25)-H(25)	119.5
C(15)-C(14)-C(13)	120.9(8)	C(21)-C(26)-C(25)	120.5(8)
C(15)-C(14)-H(14)	119.5	C(21)-C(26)-C(27)	122.2(7)
C(13)-C(14)-H(14)	119.5	C(25)-C(26)-C(27)	117.3(7)

N(6)-C(27)-O(4)	116.4(7)	C(34)-C(33)-H(33)	119.9
N(6)-C(27)-C(26)	127.2(7)	C(32)-C(33)-H(33)	119.9
O(4)-C(27)-C(26)	116.4(7)	C(33)-C(34)-C(35)	119.9(9)
O(4)-C(28)-C(29)	104.6(6)	C(33)-C(34)-H(34)	120.1
O(4)-C(28)-H(28A)	110.8	C(35)-C(34)-H(34)	120.1
C(29)-C(28)-H(28A)	110.8	C(30)-C(35)-C(34)	120.7(9)
O(4)-C(28)-H(28B)	110.8	C(30)-C(35)-H(35)	119.6
C(29)-C(28)-H(28B)	110.8	C(34)-C(35)-H(35)	119.6
H(28A)-C(28)-H(28B)	108.9	C(36)-O(5)-Ni(1)	127.7(5)
N(6)-C(29)-C(30)	112.0(6)	C(36)-O(6)-Ni(2)	112.1(5)
N(6)-C(29)-C(28)	101.8(6)	O(6)-C(36)-O(5)	122.5(7)
C(30)-C(29)-C(28)	113.2(6)	O(6)-C(36)-C(37)	120.5(7)
N(6)-C(29)-H(29)	109.9	O(5)-C(36)-C(37)	117.0(7)
C(30)-C(29)-H(29)	109.9	C(36)-C(37)-H(37A)	109.5
C(28)-C(29)-H(29)	109.9	C(36)-C(37)-H(37B)	109.5
C(31)-C(30)-C(35)	118.3(8)	H(37A)-C(37)-H(37B)	109.5
C(31)-C(30)-C(29)	121.2(7)	C(36)-C(37)-H(37C)	109.5
C(35)-C(30)-C(29)	120.4(7)	H(37A)-C(37)-H(37C)	109.5
C(32)-C(31)-C(30)	120.5(8)	H(37B)-C(37)-H(37C)	109.5
C(32)-C(31)-H(31)	119.8	Cl(1)-C(38)-Cl(2)	111.0(5)
C(30)-C(31)-H(31)	119.8	Cl(1)-C(38)-H(38A)	109.4
C(33)-C(32)-C(31)	120.4(9)	Cl(2)-C(38)-H(38A)	109.4
C(33)-C(32)-H(32)	119.8	Cl(1)-C(38)-H(38B)	109.4
C(31)-C(32)-H(32)	119.8	Cl(2)-C(38)-H(38B)	109.4
C(34)-C(33)-C(32)	120.2(9)	H(38A)-C(38)-H(38B)	108.0

Table S10. Anisotropic displacement parameters ($Å^2$x 10^3) for **25•Ni2OAc**. The anisotropic displacement factor exponent takes the form: $-2\pi^2[h^2 a^{*2}U^{11} + ... + 2 h k a^* b^* U^{12}]$

U^{11}	U^{22}	U^{33}	U^{23}	U^{13}	U^{12}	
Ni(1)	13(1)	11(1)	12(1)	2(1)	4(1)	1(1)
Ni(2)	11(1)	11(1)	13(1)	2(1)	4(1)	1(1)
O(1)	21(3)	14(3)	26(3)	6(3)	11(2)	-2(2)
O(2)	19(3)	20(3)	27(3)	10(3)	5(2)	1(2)
O(3)	14(3)	20(3)	27(3)	6(3)	6(2)	1(2)
O(4)	20(3)	17(3)	24(3)	10(2)	9(2)	2(2)
N(1)	14(2)	10(3)	12(3)	1(2)	3(2)	-4(2)
N(2)	18(3)	11(3)	14(3)	2(2)	7(2)	-2(2)
N(3)	14(3)	19(3)	12(3)	4(3)	7(2)	1(2)
N(4)	13(3)	8(3)	13(3)	0(2)	3(2)	1(2)
N(5)	11(3)	13(3)	15(3)	3(2)	5(2)	2(2)
N(6)	14(3)	12(3)	14(3)	0(2)	6(2)	1(2)
C(1)	16(3)	13(4)	16(4)	-2(3)	5(3)	-3(3)
C(2)	16(3)	13(3)	12(3)	-5(3)	7(3)	-4(3)
C(3)	21(3)	10(3)	15(3)	-5(3)	10(3)	-2(3)
C(4)	26(3)	12(3)	13(3)	3(3)	11(3)	-2(3)
C(5)	24(3)	17(4)	15(4)	1(3)	9(3)	-3(3)
C(6)	31(4)	15(4)	18(4)	1(3)	3(3)	-7(3)
C(7)	37(4)	16(4)	24(4)	6(3)	7(3)	-4(3)
C(8)	31(4)	22(4)	25(4)	9(3)	8(3)	2(3)
C(9)	25(3)	16(3)	12(3)	2(3)	10(3)	0(3)
C(10)	18(3)	18(4)	11(3)	2(3)	6(3)	7(3)
C(11)	15(3)	21(4)	23(4)	9(3)	9(3)	3(3)
C(12)	13(3)	20(4)	11(3)	6(3)	3(3)	5(3)
C(13)	10(3)	24(4)	14(3)	5(3)	0(3)	4(3)
C(14)	16(3)	27(4)	21(4)	0(3)	2(3)	-3(3)
C(15)	20(4)	31(4)	31(4)	1(4)	4(3)	-3(3)
C(16)	24(4)	32(4)	30(4)	-13(4)	-4(3)	6(3)
C(17)	22(4)	37(5)	26(4)	-1(3)	6(3)	10(3)
C(18)	19(3)	30(4)	21(4)	3(3)	8(3)	4(3)
C(19)	12(3)	13(3)	15(3)	-1(3)	7(3)	1(3)

C(20)	13(3)	18(4)	19(4)	0(3)	5(3)	-1(3)
C(21)	12(3)	11(3)	14(3)	-1(3)	-1(3)	-1(2)
C(22)	16(3)	16(4)	21(4)	0(3)	4(3)	0(3)
C(23)	20(3)	14(4)	30(4)	0(3)	5(3)	5(3)
C(24)	24(4)	15(4)	24(4)	5(3)	4(3)	0(3)
C(25)	22(3)	16(4)	18(4)	5(3)	6(3)	-5(3)
C(26)	14(3)	15(3)	15(3)	-1(3)	0(3)	-2(3)
C(27)	17(3)	17(4)	14(3)	5(3)	4(3)	0(3)
C(28)	19(3)	18(4)	25(4)	3(3)	11(3)	0(3)
C(29)	11(3)	18(4)	19(3)	2(3)	8(2)	-1(3)
C(30)	20(3)	17(3)	14(3)	0(3)	15(3)	0(3)
C(31)	35(4)	23(4)	19(4)	3(3)	8(3)	7(3)
C(32)	48(5)	23(4)	28(4)	1(4)	14(4)	6(4)
C(33)	43(4)	25(4)	28(4)	-10(4)	21(3)	-5(4)
C(34)	30(4)	30(5)	30(4)	-9(4)	7(3)	-1(3)
C(35)	20(3)	25(4)	25(4)	-6(3)	8(3)	-1(3)
O(5)	18(2)	14(3)	13(3)	3(2)	5(2)	1(2)
O(6)	16(2)	15(3)	14(2)	1(2)	5(2)	4(2)
C(36)	11(3)	13(4)	16(3)	-1(3)	9(3)	4(3)
C(37)	20(4)	14(4)	17(4)	2(3)	3(3)	-1(3)
C(38)	27(4)	23(4)	30(5)	9(4)	4(4)	-3(4)
Cl(1)	40(1)	24(1)	32(1)	8(1)	6(1)	-9(1)
Cl(2)	43(1)	18(1)	34(1)	0(1)	4(1)	-4(1)

Table S11. Hydrogen coordinates (x 10^4) and isotropic displacement parameters ($Å^2$x 10^3) for **25•Ni2OAc**.

x	y	z	U(eq)	
H(1)	-4555	462	2449	18
H(5)	-2475	-2079	4036	22
H(6)	-1849	-3305	4858	27
H(7)	778	-3656	5803	32
H(8)	2757	-2789	5960	31
H(11A)	5959	-1292	5550	23
H(11B)	5810	-879	6729	23
H(12)	4610	-342	4398	18
H(14)	5679	876	4887	27
H(15)	6080	1998	5999	34
H(16)	5057	2131	7553	38
H(17)	3749	1123	8080	34
H(18)	3237	15	6918	27
H(22)	-4738	2948	196	22
H(23)	-5290	4025	-1009	27
H(24)	-3809	4334	-2231	26
H(25)	-1702	3582	-2186	23
H(28A)	2365	2347	-1279	24
H(28B)	1408	1849	-2445	24
H(29)	2180	1361	-137	18
H(31)	2597	46	-404	31
H(32)	1947	-1097	-1467	39
H(33)	-270	-1177	-3187	36
H(34)	-1861	-121	-3851	37
H(35)	-1261	1028	-2769	28
H(37A)	2121	2053	2448	27
H(37B)	3423	1658	2001	27
H(37C)	3618	1656	3404	27
H(38A)	1411	4188	-718	34
H(38B)	669	4410	297	34

Table S12. Torsion angles [°] for **25•Ni2OAc**.

O(5)-Ni(1)-N(1)-C(2)	-155.5(5)	N(1)-C(2)-C(3)-O(1)	178.6(7)
N(2)-Ni(1)-N(1)-C(2)	18.5(5)	C(1)-C(2)-C(3)-O(1)	-6.1(14)
O(5)-Ni(1)-N(1)-N(4)	6.8(8)	N(1)-C(2)-C(3)-N(2)	-1.4(9)
N(2)-Ni(1)-N(1)-N(4)	-179.1(8)	C(1)-C(2)-C(3)-N(2)	173.9(8)
O(5)-Ni(1)-N(3)-C(10)	-172.4(7)	C(3)-N(2)-C(4)-C(9)	-159.2(7)
N(2)-Ni(1)-N(3)-C(10)	13.6(7)	Ni(1)-N(2)-C(4)-C(9)	17.8(11)
O(5)-Ni(1)-N(3)-C(12)	6.1(6)	C(3)-N(2)-C(4)-C(5)	21.2(11)
N(2)-Ni(1)-N(3)-C(12)	-167.9(6)	Ni(1)-N(2)-C(4)-C(5)	-161.7(6)
C(2)-N(1)-N(4)-C(19)	-1.9(8)	N(2)-C(4)-C(5)-C(6)	178.2(8)
Ni(1)-N(1)-N(4)-C(19)	-164.8(6)	C(9)-C(4)-C(5)-C(6)	-1.4(12)
C(2)-N(1)-N(4)-Ni(2)	-162.0(6)	C(4)-C(5)-C(6)-C(7)	0.0(13)
Ni(1)-N(1)-N(4)-Ni(2)	35.1(12)	C(5)-C(6)-C(7)-C(8)	0.8(14)
N(5)-Ni(2)-N(4)-N(1)	172.9(7)	C(6)-C(7)-C(8)-C(9)	-0.2(14)
O(6)-Ni(2)-N(4)-N(1)	-9.9(7)	C(7)-C(8)-C(9)-C(4)	-1.2(13)
N(5)-Ni(2)-N(4)-C(19)	13.4(6)	C(7)-C(8)-C(9)-C(10)	-179.6(8)
O(6)-Ni(2)-N(4)-C(19)	-169.4(6)	N(2)-C(4)-C(9)-C(8)	-177.6(8)
N(4)-Ni(2)-N(5)-C(20)	-14.8(6)	C(5)-C(4)-C(9)-C(8)	2.0(12)
N(6)-Ni(2)-N(5)-C(20)	160.3(6)	N(2)-C(4)-C(9)-C(10)	0.6(12)
N(4)-Ni(2)-N(5)-C(21)	167.2(7)	C(5)-C(4)-C(9)-C(10)	-179.7(7)
N(6)-Ni(2)-N(5)-C(21)	-17.7(7)	C(12)-N(3)-C(10)-O(2)	0.0(10)
N(5)-Ni(2)-N(6)-C(27)	11.4(7)	Ni(1)-N(3)-C(10)-O(2)	178.7(5)
O(6)-Ni(2)-N(6)-C(27)	-166.5(7)	C(12)-N(3)-C(10)-C(9)	179.9(8)
N(5)-Ni(2)-N(6)-C(29)	-167.2(6)	Ni(1)-N(3)-C(10)-C(9)	-1.4(12)
O(6)-Ni(2)-N(6)-C(29)	14.9(6)	C(11)-O(2)-C(10)-N(3)	-3.5(10)
N(4)-N(1)-C(2)-C(1)	1.7(9)	C(11)-O(2)-C(10)-C(9)	176.6(7)
Ni(1)-N(1)-C(2)-C(1)	169.5(5)	C(8)-C(9)-C(10)-N(3)	169.0(9)
N(4)-N(1)-C(2)-C(3)	178.0(6)	C(4)-C(9)-C(10)-N(3)	-9.3(13)
Ni(1)-N(1)-C(2)-C(3)	-14.2(8)	C(8)-C(9)-C(10)-O(2)	-11.1(11)
C(19)-C(1)-C(2)-N(1)	-0.8(9)	C(4)-C(9)-C(10)-O(2)	170.5(7)
C(19)-C(1)-C(2)-C(3)	-176.2(8)	C(10)-O(2)-C(11)-C(12)	5.2(8)
C(4)-N(2)-C(3)-O(1)	13.8(12)	C(10)-N(3)-C(12)-C(13)	-117.5(8)
Ni(1)-N(2)-C(3)-O(1)	-163.7(7)	Ni(1)-N(3)-C(12)-C(13)	63.8(8)
C(4)-N(2)-C(3)-C(2)	-166.2(7)	C(10)-N(3)-C(12)-C(11)	3.2(8)
Ni(1)-N(2)-C(3)-C(2)	16.3(8)	Ni(1)-N(3)-C(12)-C(11)	-175.5(5)

O(2)-C(11)-C(12)-C(13)	116.7(7)	C(23)-C(24)-C(25)-C(26)	2.0(13)
O(2)-C(11)-C(12)-N(3)	-4.9(8)	N(5)-C(21)-C(26)-C(25)	179.0(7)
N(3)-C(12)-C(13)-C(18)	48.4(10)	C(22)-C(21)-C(26)-C(25)	-2.7(11)
C(11)-C(12)-C(13)-C(18)	-66.2(9)	N(5)-C(21)-C(26)-C(27)	1.4(11)
N(3)-C(12)-C(13)-C(14)	-135.4(7)	C(22)-C(21)-C(26)-C(27)	179.6(7)
C(11)-C(12)-C(13)-C(14)	110.0(8)	C(24)-C(25)-C(26)-C(21)	0.2(12)
C(18)-C(13)-C(14)-C(15)	0.0(11)	C(24)-C(25)-C(26)-C(27)	177.9(8)
C(12)-C(13)-C(14)-C(15)	-176.4(7)	C(29)-N(6)-C(27)-O(4)	-4.5(9)
C(13)-C(14)-C(15)-C(16)	0.0(13)	Ni(2)-N(6)-C(27)-O(4)	176.7(5)
C(14)-C(15)-C(16)-C(17)	1.5(13)	C(29)-N(6)-C(27)-C(26)	177.9(7)
C(15)-C(16)-C(17)-C(18)	-3.1(13)	Ni(2)-N(6)-C(27)-C(26)	-0.9(12)
C(16)-C(17)-C(18)-C(13)	3.1(13)	C(28)-O(4)-C(27)-N(6)	-4.6(9)
C(14)-C(13)-C(18)-C(17)	-1.6(12)	C(28)-O(4)-C(27)-C(26)	173.3(7)
C(12)-C(13)-C(18)-C(17)	174.7(7)	C(21)-C(26)-C(27)-N(6)	-8.4(13)
N(1)-N(4)-C(19)-C(1)	1.5(9)	C(25)-C(26)-C(27)-N(6)	173.9(8)
Ni(2)-N(4)-C(19)-C(1)	166.7(5)	C(21)-C(26)-C(27)-O(4)	173.9(7)
N(1)-N(4)-C(19)-C(20)	-175.0(6)	C(25)-C(26)-C(27)-O(4)	-3.7(10)
Ni(2)-N(4)-C(19)-C(20)	-9.8(9)	C(27)-O(4)-C(28)-C(29)	11.1(8)
C(2)-C(1)-C(19)-N(4)	-0.4(9)	C(27)-N(6)-C(29)-C(30)	-110.3(7)
C(2)-C(1)-C(19)-C(20)	175.1(9)	Ni(2)-N(6)-C(29)-C(30)	68.5(8)
C(21)-N(5)-C(20)-O(3)	13.3(13)	C(27)-N(6)-C(29)-C(28)	10.8(8)
Ni(2)-N(5)-C(20)-O(3)	-164.8(7)	Ni(2)-N(6)-C(29)-C(28)	-170.3(5)
C(21)-N(5)-C(20)-C(19)	-169.2(7)	O(4)-C(28)-C(29)-N(6)	-12.9(7)
Ni(2)-N(5)-C(20)-C(19)	12.7(8)	O(4)-C(28)-C(29)-C(30)	107.5(7)
N(4)-C(19)-C(20)-O(3)	175.7(7)	N(6)-C(29)-C(30)-C(31)	-124.5(8)
C(1)-C(19)-C(20)-O(3)	0.3(14)	C(28)-C(29)-C(30)-C(31)	121.2(8)
N(4)-C(19)-C(20)-N(5)	-2.0(10)	N(6)-C(29)-C(30)-C(35)	57.8(9)
C(1)-C(19)-C(20)-N(5)	-177.3(9)	C(28)-C(29)-C(30)-C(35)	-56.5(9)
C(20)-N(5)-C(21)-C(26)	-164.1(7)	C(35)-C(30)-C(31)-C(32)	0.8(13)
Ni(2)-N(5)-C(21)-C(26)	13.7(10)	C(29)-C(30)-C(31)-C(32)	-177.0(8)
C(20)-N(5)-C(21)-C(22)	17.7(11)	C(30)-C(31)-C(32)-C(33)	0.2(15)
Ni(2)-N(5)-C(21)-C(22)	-164.4(6)	C(31)-C(32)-C(33)-C(34)	-0.4(15)
C(26)-C(21)-C(22)-C(23)	3.2(11)	C(32)-C(33)-C(34)-C(35)	-0.4(15)
N(5)-C(21)-C(22)-C(23)	-178.5(7)	C(31)-C(30)-C(35)-C(34)	-1.6(12)
C(21)-C(22)-C(23)-C(24)	-1.2(12)	C(29)-C(30)-C(35)-C(34)	176.2(8)
C(22)-C(23)-C(24)-C(25)	-1.5(13)	C(33)-C(34)-C(35)-C(30)	1.4(14)

N(3)-Ni(1)-O(5)-C(36)	153.6(7)
N(1)-Ni(1)-O(5)-C(36)	-32.0(6)
N(4)-Ni(2)-O(6)-C(36)	-72.8(6)
N(6)-Ni(2)-O(6)-C(36)	112.0(6)
Ni(2)-O(6)-C(36)-O(5)	112.8(7)
Ni(2)-O(6)-C(36)-C(37)	-69.0(8)
Ni(1)-O(5)-C(36)-O(6)	-24.9(10)
Ni(1)-O(5)-C(36)-C(37)	156.9(5)

Table S13. Hydrogen bonds for **25•Ni2OAc** [Å and °].

D-H...A	d(D-H)	d(H...A)	d(D...A)	<(DHA)
C(5)-H(5)...O(1)	0.95	2.19	2.752(10)	116.5
C(11)-H(11A)...O(1)#1	0.99	2.28	3.042(10)	132.8
C(12)-H(12)...O(1)#1	1.00	2.43	3.132(10)	126.9
C(22)-H(22)...O(3)	0.95	2.19	2.763(10)	117.6
C(29)-H(29)...O(3)#1	1.00	2.44	3.278(9)	141.0
C(37)-H(37B)...O(3)#1	0.98	2.51	3.493(10)	178.3

Symmetry transformations used to generate equivalent atoms: #1 x+1,y,z

Figure S3. ORTEP diagram of **30•Pd2Br** with H atoms and solvate atoms removed and ellipsoids drawn at the 30% level.

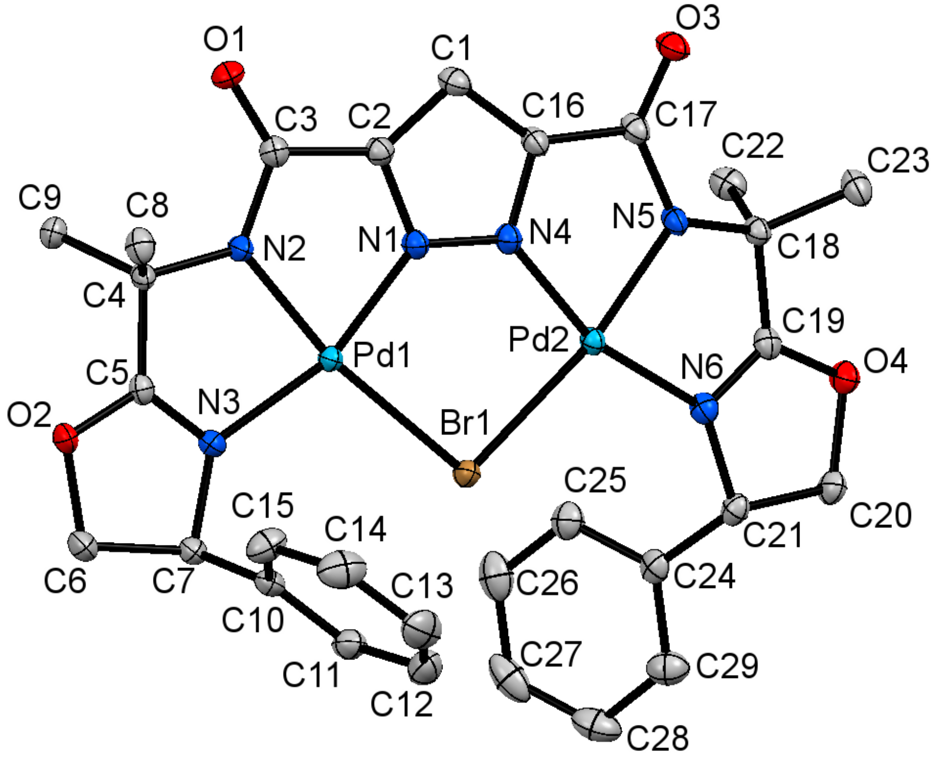

Table S14. Crystal data and structure refinement for **30•Pd2Br**.

Identification code	30-Pd2Br
Empirical formula	C31 H33 Br Cl2 N6 O4 Pd2
Formula weight	917.24
Temperature	100(2) K
Wavelength	0.71073 Å
Crystal system	Monoclinic
Space group	$P2_1$
Unit cell dimensions	a = 9.8982(7) Å α= 90°.
b = 10.0139(7) Å	β= 101.2770(10)°.
c = 16.9675(12) Å	γ = 90°.
Volume	1649.3(2) $Å^3$
Z	2
Density (calculated)	1.847 Mg/m^3
Absorption coefficient	2.510 mm^{-1}
F(000)	908
Crystal size	0.390 x 0.180 x 0.060 mm^3
Theta range for data collection	2.098 to 31.527°.
Index ranges	-14<=h<=14, -14<=k<=14, -24<=l<=24
Reflections collected	20782
Independent reflections	10821 [R(int) = 0.0208]
Completeness to theta = 25.242°	100.0 %
Absorption correction	Semi-empirical from equivalents
Max. and min. transmission	.9999 and 0.7329
Refinement method	Full-matrix least-squares on F^2
Data / restraints / parameters	10821 / 1 / 419
Goodness-of-fit on F^2	0.829
Final R indices [I>2sigma(I)]	R1 = 0.0226, wR2 = 0.0462
R indices (all data)	R1 = 0.0252, wR2 = 0.0469
Absolute structure parameter	0.016(3)
Extinction coefficient	n/a
Largest diff. peak and hole	0.740 and -0.450 e.$Å^{-3}$

Table S15. Atomic coordinates (x 10^4) and equivalent isotropic displacement parameters (Å^{2}x 10^3) for **30•Pd2Br**. U(eq) is defined as one third of the trace of the orthogonalized U^{ij} tensor.

x	y	z	U(eq)	
Pd(1)	5691(1)	8142(1)	3770(1)	13(1)
Pd(2)	2360(1)	7364(1)	2278(1)	13(1)
Br(1)	3181(1)	8780(1)	3513(1)	16(1)
O(1)	8827(2)	5980(2)	3156(1)	21(1)
O(2)	8692(2)	9374(2)	5589(1)	18(1)
O(3)	3180(2)	4631(2)	657(1)	22(1)
O(4)	-1318(2)	7507(2)	744(1)	20(1)
N(1)	5319(2)	6895(3)	2890(2)	16(1)
N(2)	7558(2)	7453(3)	3781(1)	16(1)
N(3)	6690(3)	9199(2)	4720(2)	15(1)
N(4)	4160(2)	6558(3)	2414(2)	16(1)
N(5)	2060(3)	6092(3)	1373(2)	16(1)
N(6)	399(3)	7888(3)	1788(2)	17(1)
C(1)	5834(3)	5408(3)	2019(2)	18(1)
C(2)	6371(3)	6220(3)	2682(2)	16(1)
C(3)	7734(3)	6516(3)	3224(2)	17(1)
C(4)	8672(3)	7885(3)	4449(2)	14(1)
C(5)	7972(3)	8859(3)	4914(2)	15(1)
C(6)	7763(3)	10314(3)	5886(2)	23(1)
C(7)	6322(3)	9986(3)	5390(2)	17(1)
C(8)	9193(3)	6718(3)	5008(2)	20(1)
C(9)	9847(3)	8577(3)	4141(2)	17(1)
C(10)	5418(3)	9113(3)	5803(2)	17(1)
C(11)	4010(3)	9364(3)	5681(2)	22(1)
C(12)	3155(4)	8509(4)	6002(2)	28(1)
C(13)	3695(4)	7407(4)	6434(2)	33(1)
C(14)	5097(4)	7158(4)	6572(2)	33(1)
C(15)	5956(3)	8006(4)	6247(2)	25(1)
C(16)	4418(3)	5672(3)	1865(2)	16(1)
C(17)	3166(3)	5383(3)	1229(2)	17(1)
C(18)	814(3)	6284(3)	750(2)	16(1)

C(19)	-42(3)	7254(3)	1125(2)	17(1)
C(20)	-1867(3)	8535(3)	1217(2)	20(1)
C(21)	-684(3)	8788(3)	1954(2)	16(1)
C(22)	1173(3)	6942(3)	-3(2)	22(1)
C(23)	4(4)	4990(3)	536(2)	23(1)
C(24)	-231(3)	10224(3)	2070(2)	18(1)
C(25)	987(3)	10697(3)	1879(2)	22(1)
C(26)	1355(4)	12025(4)	1999(2)	31(1)
C(27)	518(4)	12903(4)	2310(2)	33(1)
C(28)	-709(4)	12452(4)	2495(2)	33(1)
C(29)	-1066(4)	11115(4)	2374(2)	26(1)
Cl(1)	4021(1)	7049(1)	8913(1)	37(1)
Cl(2)	5857(1)	4284(1)	8905(1)	40(1)
C(30)	5590(4)	6707(4)	9599(2)	34(1)
C(31)	5762(4)	5245(4)	9781(2)	28(1)

Table S16. Bond lengths [Å] and angles [°] for **30•Pd2Br**.

Pd(1)-N(1)	1.925(2)	C(6)-H(6B)	0.9900
Pd(1)-N(2)	1.970(2)	C(7)-C(10)	1.517(4)
Pd(1)-N(3)	2.018(2)	C(7)-H(7)	1.0000
Pd(1)-Br(1)	2.5192(4)	C(8)-H(8A)	0.9800
Pd(2)-N(4)	1.928(3)	C(8)-H(8B)	0.9800
Pd(2)-N(5)	1.972(2)	C(8)-H(8C)	0.9800
Pd(2)-N(6)	2.025(2)	C(9)-H(9A)	0.9800
Pd(2)-Br(1)	2.5295(4)	C(9)-H(9B)	0.9800
O(1)-C(3)	1.233(4)	C(9)-H(9C)	0.9800
O(2)-C(5)	1.329(3)	C(10)-C(15)	1.386(5)
O(2)-C(6)	1.472(4)	C(10)-C(11)	1.391(4)
O(3)-C(17)	1.230(4)	C(11)-C(12)	1.387(5)
O(4)-C(19)	1.326(3)	C(11)-H(11)	0.9500
O(4)-C(20)	1.473(4)	C(12)-C(13)	1.373(6)
N(1)-N(4)	1.312(3)	C(12)-H(12)	0.9500
N(1)-C(2)	1.345(4)	C(13)-C(14)	1.384(5)
N(2)-C(3)	1.366(4)	C(13)-H(13)	0.9500
N(2)-C(4)	1.483(3)	C(14)-C(15)	1.390(5)
N(3)-C(5)	1.293(4)	C(14)-H(14)	0.9500
N(3)-C(7)	1.487(4)	C(15)-H(15)	0.9500
N(4)-C(16)	1.348(4)	C(16)-C(17)	1.504(4)
N(5)-C(17)	1.366(4)	C(18)-C(19)	1.509(4)
N(5)-C(18)	1.472(4)	C(18)-C(23)	1.530(4)
N(6)-C(19)	1.292(4)	C(18)-C(22)	1.539(4)
N(6)-C(21)	1.468(4)	C(20)-C(21)	1.557(4)
C(1)-C(16)	1.400(4)	C(20)-H(20A)	0.9900
C(1)-C(2)	1.405(4)	C(20)-H(20B)	0.9900
C(1)-H(1)	0.9500	C(21)-C(24)	1.508(5)
C(2)-C(3)	1.507(4)	C(21)-H(21)	1.0000
C(4)-C(5)	1.506(4)	C(22)-H(22A)	0.9800
C(4)-C(8)	1.530(4)	C(22)-H(22B)	0.9800
C(4)-C(9)	1.531(4)	C(22)-H(22C)	0.9800
C(6)-C(7)	1.543(4)	C(23)-H(23A)	0.9800
C(6)-H(6A)	0.9900	C(23)-H(23B)	0.9800

C(23)-H(23C)	0.9800	C(28)-H(28)	0.9500
C(24)-C(29)	1.383(5)	C(29)-H(29)	0.9500
C(24)-C(25)	1.391(4)	Cl(1)-C(30)	1.782(4)
C(25)-C(26)	1.384(5)	Cl(2)-C(31)	1.788(4)
C(25)-H(25)	0.9500	C(30)-C(31)	1.500(5)
C(26)-C(27)	1.381(6)	C(30)-H(30A)	0.9900
C(26)-H(26)	0.9500	C(30)-H(30B)	0.9900
C(27)-C(28)	1.388(6)	C(31)-H(31A)	0.9900
C(27)-H(27)	0.9500	C(31)-H(31B)	0.9900
C(28)-C(29)	1.390(5)		

N(1)-Pd(1)-N(2)	79.43(10)	N(1)-N(4)-Pd(2)	130.0(2)
N(1)-Pd(1)-N(3)	161.55(10)	C(16)-N(4)-Pd(2)	119.2(2)
N(2)-Pd(1)-N(3)	82.16(10)	C(17)-N(5)-C(18)	121.8(2)
N(1)-Pd(1)-Br(1)	89.89(7)	C(17)-N(5)-Pd(2)	118.14(19)
N(2)-Pd(1)-Br(1)	169.21(7)	C(18)-N(5)-Pd(2)	116.88(19)
N(3)-Pd(1)-Br(1)	108.55(7)	C(19)-N(6)-C(21)	109.5(2)
N(4)-Pd(2)-N(5)	79.51(10)	C(19)-N(6)-Pd(2)	111.3(2)
N(4)-Pd(2)-N(6)	161.22(10)	C(21)-N(6)-Pd(2)	139.2(2)
N(5)-Pd(2)-N(6)	82.09(10)	C(16)-C(1)-C(2)	104.3(3)
N(4)-Pd(2)-Br(1)	89.73(7)	C(16)-C(1)-H(1)	127.8
N(5)-Pd(2)-Br(1)	169.15(7)	C(2)-C(1)-H(1)	127.8
N(6)-Pd(2)-Br(1)	108.74(7)	N(1)-C(2)-C(1)	108.1(3)
Pd(1)-Br(1)-Pd(2)	98.480(12)	N(1)-C(2)-C(3)	112.7(3)
C(5)-O(2)-C(6)	106.1(2)	C(1)-C(2)-C(3)	139.1(3)
C(19)-O(4)-C(20)	106.6(2)	O(1)-C(3)-N(2)	126.6(3)
N(4)-N(1)-C(2)	109.7(2)	O(1)-C(3)-C(2)	123.6(3)
N(4)-N(1)-Pd(1)	130.9(2)	N(2)-C(3)-C(2)	109.8(2)
C(2)-N(1)-Pd(1)	119.33(19)	N(2)-C(4)-C(5)	103.9(2)
C(3)-N(2)-C(4)	123.6(2)	N(2)-C(4)-C(8)	111.2(2)
C(3)-N(2)-Pd(1)	118.68(19)	C(5)-C(4)-C(8)	107.7(2)
C(4)-N(2)-Pd(1)	117.44(18)	N(2)-C(4)-C(9)	111.9(2)
C(5)-N(3)-C(7)	108.7(2)	C(5)-C(4)-C(9)	110.1(2)
C(5)-N(3)-Pd(1)	111.7(2)	C(8)-C(4)-C(9)	111.7(2)
C(7)-N(3)-Pd(1)	137.22(19)	N(3)-C(5)-O(2)	116.9(3)
N(1)-N(4)-C(16)	109.5(2)	N(3)-C(5)-C(4)	124.5(3)

O(2)-C(5)-C(4)	118.6(2)	C(14)-C(13)-H(13)	119.8
O(2)-C(6)-C(7)	104.5(2)	C(13)-C(14)-C(15)	119.6(3)
O(2)-C(6)-H(6A)	110.9	C(13)-C(14)-H(14)	120.2
C(7)-C(6)-H(6A)	110.9	C(15)-C(14)-H(14)	120.2
O(2)-C(6)-H(6B)	110.9	C(10)-C(15)-C(14)	120.3(3)
C(7)-C(6)-H(6B)	110.9	C(10)-C(15)-H(15)	119.8
H(6A)-C(6)-H(6B)	108.9	C(14)-C(15)-H(15)	119.8
N(3)-C(7)-C(10)	107.6(2)	N(4)-C(16)-C(1)	108.3(3)
N(3)-C(7)-C(6)	101.0(2)	N(4)-C(16)-C(17)	112.5(3)
C(10)-C(7)-C(6)	115.9(3)	C(1)-C(16)-C(17)	138.7(3)
N(3)-C(7)-H(7)	110.6	O(3)-C(17)-N(5)	126.3(3)
C(10)-C(7)-H(7)	110.6	O(3)-C(17)-C(16)	123.3(3)
C(6)-C(7)-H(7)	110.6	N(5)-C(17)-C(16)	110.4(3)
C(4)-C(8)-H(8A)	109.5	N(5)-C(18)-C(19)	104.0(2)
C(4)-C(8)-H(8B)	109.5	N(5)-C(18)-C(23)	112.7(2)
H(8A)-C(8)-H(8B)	109.5	C(19)-C(18)-C(23)	109.5(3)
C(4)-C(8)-H(8C)	109.5	N(5)-C(18)-C(22)	110.8(3)
H(8A)-C(8)-H(8C)	109.5	C(19)-C(18)-C(22)	108.4(3)
H(8B)-C(8)-H(8C)	109.5	C(23)-C(18)-C(22)	111.2(3)
C(4)-C(9)-H(9A)	109.5	N(6)-C(19)-O(4)	117.2(3)
C(4)-C(9)-H(9B)	109.5	N(6)-C(19)-C(18)	124.2(3)
H(9A)-C(9)-H(9B)	109.5	O(4)-C(19)-C(18)	118.5(3)
C(4)-C(9)-H(9C)	109.5	O(4)-C(20)-C(21)	104.6(2)
H(9A)-C(9)-H(9C)	109.5	O(4)-C(20)-H(20A)	110.8
H(9B)-C(9)-H(9C)	109.5	C(21)-C(20)-H(20A)	110.8
C(15)-C(10)-C(11)	119.4(3)	O(4)-C(20)-H(20B)	110.8
C(15)-C(10)-C(7)	120.9(3)	C(21)-C(20)-H(20B)	110.8
C(11)-C(10)-C(7)	119.4(3)	H(20A)-C(20)-H(20B)	108.9
C(12)-C(11)-C(10)	120.0(3)	N(6)-C(21)-C(24)	113.6(2)
C(12)-C(11)-H(11)	120.0	N(6)-C(21)-C(20)	102.1(2)
C(10)-C(11)-H(11)	120.0	C(24)-C(21)-C(20)	114.7(3)
C(13)-C(12)-C(11)	120.1(3)	N(6)-C(21)-H(21)	108.7
C(13)-C(12)-H(12)	119.9	C(24)-C(21)-H(21)	108.7
C(11)-C(12)-H(12)	119.9	C(20)-C(21)-H(21)	108.7
C(12)-C(13)-C(14)	120.5(3)	C(18)-C(22)-H(22A)	109.5
C(12)-C(13)-H(13)	119.8	C(18)-C(22)-H(22B)	109.5

H(22A)-C(22)-H(22B)	109.5	C(26)-C(27)-H(27)	120.1
C(18)-C(22)-H(22C)	109.5	C(28)-C(27)-H(27)	120.1
H(22A)-C(22)-H(22C)	109.5	C(27)-C(28)-C(29)	119.3(4)
H(22B)-C(22)-H(22C)	109.5	C(27)-C(28)-H(28)	120.4
C(18)-C(23)-H(23A)	109.5	C(29)-C(28)-H(28)	120.4
C(18)-C(23)-H(23B)	109.5	C(24)-C(29)-C(28)	121.5(4)
H(23A)-C(23)-H(23B)	109.5	C(24)-C(29)-H(29)	119.2
C(18)-C(23)-H(23C)	109.5	C(28)-C(29)-H(29)	119.2
H(23A)-C(23)-H(23C)	109.5	C(31)-C(30)-Cl(1)	111.8(3)
H(23B)-C(23)-H(23C)	109.5	C(31)-C(30)-H(30A)	109.3
C(29)-C(24)-C(25)	118.4(3)	Cl(1)-C(30)-H(30A)	109.3
C(29)-C(24)-C(21)	118.7(3)	C(31)-C(30)-H(30B)	109.3
C(25)-C(24)-C(21)	122.9(3)	Cl(1)-C(30)-H(30B)	109.3
C(26)-C(25)-C(24)	120.5(3)	H(30A)-C(30)-H(30B)	107.9
C(26)-C(25)-H(25)	119.7	C(30)-C(31)-Cl(2)	112.1(3)
C(24)-C(25)-H(25)	119.7	C(30)-C(31)-H(31A)	109.2
C(27)-C(26)-C(25)	120.5(4)	Cl(2)-C(31)-H(31A)	109.2
C(27)-C(26)-H(26)	119.7	C(30)-C(31)-H(31B)	109.2
C(25)-C(26)-H(26)	119.7	Cl(2)-C(31)-H(31B)	109.2
C(26)-C(27)-C(28)	119.8(3)	H(31A)-C(31)-H(31B)	107.9

Table S17. Anisotropic displacement parameters ($Å^2$x 10^3) for **30•Pd2Br**. The anisotropic displacement factor exponent takes the form: $-2\pi^2[h^2 a^{*2}U^{11} + ... + 2 h k a^* b^* U^{12}]$

U^{11}	U^{22}	U^{33}	U^{23}	U^{13}	U^{12}	
Pd(1)	11(1)	14(1)	13(1)	-2(1)	1(1)	1(1)
Pd(2)	13(1)	14(1)	12(1)	-1(1)	0(1)	0(1)
Br(1)	12(1)	20(1)	16(1)	-4(1)	1(1)	1(1)
O(1)	16(1)	21(1)	26(1)	-5(1)	5(1)	4(1)
O(2)	14(1)	21(1)	18(1)	-7(1)	0(1)	-2(1)
O(3)	22(1)	19(1)	23(1)	-7(1)	-1(1)	5(1)
O(4)	16(1)	24(1)	19(1)	-2(1)	-3(1)	3(1)
N(1)	14(1)	18(1)	13(1)	-2(1)	-1(1)	1(1)
N(2)	12(1)	17(1)	17(1)	-5(1)	0(1)	2(1)
N(3)	15(1)	13(1)	16(1)	-2(1)	4(1)	-1(1)
N(4)	14(1)	18(1)	15(1)	-2(1)	-1(1)	0(1)
N(5)	15(1)	15(1)	16(1)	-3(1)	-2(1)	0(1)
N(6)	17(1)	15(1)	17(1)	0(1)	1(1)	0(1)
C(1)	21(2)	16(1)	18(2)	-2(1)	3(1)	3(1)
C(2)	16(1)	16(1)	16(1)	-1(1)	2(1)	1(1)
C(3)	16(1)	16(1)	17(1)	0(1)	2(1)	-1(1)
C(4)	12(1)	14(1)	16(1)	-3(1)	-1(1)	0(1)
C(5)	15(1)	14(1)	15(1)	1(1)	2(1)	-2(1)
C(6)	19(2)	22(2)	25(2)	-11(1)	2(1)	-2(1)
C(7)	16(1)	16(1)	18(1)	-4(1)	3(1)	-1(1)
C(8)	19(2)	18(2)	20(2)	2(1)	0(1)	-1(1)
C(9)	14(1)	19(2)	20(1)	-2(1)	4(1)	-3(1)
C(10)	18(1)	17(2)	16(1)	-5(1)	4(1)	0(1)
C(11)	20(2)	26(2)	19(2)	-4(1)	4(1)	3(1)
C(12)	21(2)	38(2)	26(2)	-7(2)	7(1)	-3(1)
C(13)	38(2)	30(2)	37(2)	-2(2)	22(2)	-10(2)
C(14)	41(2)	25(2)	35(2)	7(2)	16(2)	6(2)
C(15)	24(2)	25(2)	29(2)	5(2)	10(1)	6(2)
C(16)	17(1)	14(1)	17(1)	-2(1)	-1(1)	0(1)
C(17)	18(1)	14(1)	17(1)	-2(1)	-1(1)	2(1)
C(18)	15(1)	15(1)	16(1)	-2(1)	-1(1)	2(1)

C(19)	16(1)	16(1)	18(1)	1(1)	0(1)	-1(1)
C(20)	14(1)	24(2)	19(1)	-1(1)	-1(1)	2(1)
C(21)	13(1)	21(2)	14(1)	2(1)	1(1)	1(1)
C(22)	26(2)	19(2)	18(2)	0(1)	1(1)	3(1)
C(23)	23(2)	18(2)	25(2)	-3(1)	-3(1)	-2(1)
C(24)	16(1)	23(2)	12(1)	2(1)	-2(1)	1(1)
C(25)	20(2)	24(2)	21(2)	2(1)	0(1)	1(1)
C(26)	30(2)	32(2)	26(2)	5(2)	-7(2)	-6(2)
C(27)	49(2)	21(2)	22(2)	1(1)	-10(2)	-3(2)
C(28)	46(2)	23(2)	28(2)	-3(2)	6(2)	10(2)
C(29)	30(2)	26(2)	24(2)	1(1)	6(1)	6(1)
Cl(1)	37(1)	30(1)	42(1)	7(1)	0(1)	1(1)
Cl(2)	42(1)	36(1)	49(1)	-9(1)	22(1)	3(1)
C(30)	33(2)	27(2)	36(2)	-3(2)	-4(2)	-6(2)
C(31)	25(2)	29(2)	31(2)	0(2)	7(1)	1(1)

Table S18. Hydrogen coordinates (x 10^4) and isotropic displacement parameters ($Å^2$x 10^3) for **30•Pd2Br**.

x	y	z	U(eq)	
H(1)	6322	4815	1738	22
H(6A)	8021	11250	5796	27
H(6B)	7791	10180	6467	27
H(7)	5825	10823	5182	20
H(8A)	8430	6350	5230	29
H(8B)	9917	7034	5447	29
H(8C)	9568	6024	4705	29
H(9A)	10245	7958	3802	26
H(9B)	10558	8852	4599	26
H(9C)	9489	9366	3826	26
H(11)	3634	10121	5379	26
H(12)	2195	8686	5922	33
H(13)	3101	6812	6640	39
H(14)	5470	6412	6887	39
H(15)	6915	7827	6330	30
H(20A)	-2706	8211	1393	24
H(20B)	-2094	9362	899	24
H(21)	-990	8474	2450	20
H(22A)	1628	7800	144	32
H(22B)	327	7088	-403	32
H(22C)	1793	6356	-230	32
H(23A)	583	4337	328	34
H(23B)	-819	5174	126	34
H(23C)	-273	4630	1018	34
H(25)	1570	10103	1664	27
H(26)	2190	12336	1867	37
H(27)	781	13811	2396	40
H(28)	-1298	13051	2702	39
H(29)	-1904	10805	2504	32
H(30A)	6375	7025	9367	40

H(30B)	5601	7203	10105	40
H(31A)	4976	4927	10012	34
H(31B)	6614	5105	10188	34

Table S19. Torsion angles [°] for **30•Pd2Br**.

C(2)-N(1)-N(4)-C(16)	-1.4(4)	C(8)-C(4)-C(5)-O(2)	58.0(3)
Pd(1)-N(1)-N(4)-C(16)	177.9(2)	C(9)-C(4)-C(5)-O(2)	-64.0(3)
C(2)-N(1)-N(4)-Pd(2)	-168.3(2)	C(5)-O(2)-C(6)-C(7)	13.6(3)
Pd(1)-N(1)-N(4)-Pd(2)	10.9(4)	C(5)-N(3)-C(7)-C(10)	-107.5(3)
N(4)-N(1)-C(2)-C(1)	0.7(4)	Pd(1)-N(3)-C(7)-C(10)	52.3(4)
Pd(1)-N(1)-C(2)-C(1)	-178.7(2)	C(5)-N(3)-C(7)-C(6)	14.4(3)
N(4)-N(1)-C(2)-C(3)	-177.8(2)	Pd(1)-N(3)-C(7)-C(6)	174.2(2)
Pd(1)-N(1)-C(2)-C(3)	2.9(4)	O(2)-C(6)-C(7)-N(3)	-16.4(3)
C(16)-C(1)-C(2)-N(1)	0.2(3)	O(2)-C(6)-C(7)-C(10)	99.5(3)
C(16)-C(1)-C(2)-C(3)	178.1(4)	N(3)-C(7)-C(10)-C(15)	69.3(3)
C(4)-N(2)-C(3)-O(1)	-5.9(5)	C(6)-C(7)-C(10)-C(15)	-42.8(4)
Pd(1)-N(2)-C(3)-O(1)	-179.4(3)	N(3)-C(7)-C(10)-C(11)	-105.5(3)
C(4)-N(2)-C(3)-C(2)	174.6(3)	C(6)-C(7)-C(10)-C(11)	142.3(3)
Pd(1)-N(2)-C(3)-C(2)	1.1(3)	C(15)-C(10)-C(11)-C(12)	-0.1(5)
N(1)-C(2)-C(3)-O(1)	178.0(3)	C(7)-C(10)-C(11)-C(12)	174.8(3)
C(1)-C(2)-C(3)-O(1)	0.2(6)	C(10)-C(11)-C(12)-C(13)	-0.5(5)
N(1)-C(2)-C(3)-N(2)	-2.4(4)	C(11)-C(12)-C(13)-C(14)	1.7(5)
C(1)-C(2)-C(3)-N(2)	179.8(4)	C(12)-C(13)-C(14)-C(15)	-2.2(6)
C(3)-N(2)-C(4)-C(5)	-177.2(3)	C(11)-C(10)-C(15)-C(14)	-0.3(5)
Pd(1)-N(2)-C(4)-C(5)	-3.6(3)	C(7)-C(10)-C(15)-C(14)	-175.2(3)
C(3)-N(2)-C(4)-C(8)	-61.6(4)	C(13)-C(14)-C(15)-C(10)	1.5(5)
Pd(1)-N(2)-C(4)-C(8)	111.9(2)	N(1)-N(4)-C(16)-C(1)	1.5(4)
C(3)-N(2)-C(4)-C(9)	64.1(4)	Pd(2)-N(4)-C(16)-C(1)	170.1(2)
Pd(1)-N(2)-C(4)-C(9)	-122.4(2)	N(1)-N(4)-C(16)-C(17)	-172.1(2)
C(7)-N(3)-C(5)-O(2)	-6.8(4)	Pd(2)-N(4)-C(16)-C(17)	-3.5(4)
Pd(1)-N(3)-C(5)-O(2)	-172.2(2)	C(2)-C(1)-C(16)-N(4)	-1.0(3)
C(7)-N(3)-C(5)-C(4)	170.4(3)	C(2)-C(1)-C(16)-C(17)	170.0(4)
Pd(1)-N(3)-C(5)-C(4)	5.0(4)	C(18)-N(5)-C(17)-O(3)	-14.8(5)
C(6)-O(2)-C(5)-N(3)	-4.8(4)	Pd(2)-N(5)-C(17)-O(3)	-174.1(3)
C(6)-O(2)-C(5)-C(4)	177.9(3)	C(18)-N(5)-C(17)-C(16)	163.6(3)
N(2)-C(4)-C(5)-N(3)	-1.1(4)	Pd(2)-N(5)-C(17)-C(16)	4.4(3)
C(8)-C(4)-C(5)-N(3)	-119.1(3)	N(4)-C(16)-C(17)-O(3)	177.9(3)
C(9)-C(4)-C(5)-N(3)	118.9(3)	C(1)-C(16)-C(17)-O(3)	7.2(6)
N(2)-C(4)-C(5)-O(2)	176.0(3)	N(4)-C(16)-C(17)-N(5)	-0.6(4)

C(1)-C(16)-C(17)-N(5)	-171.4(4)	C(21)-C(24)-C(29)-C(28)	-179.9(3)
C(17)-N(5)-C(18)-C(19)	-172.9(3)	C(27)-C(28)-C(29)-C(24)	-0.3(5)
Pd(2)-N(5)-C(18)-C(19)	-13.4(3)	Cl(1)-C(30)-C(31)-Cl(2)	62.4(3)
C(17)-N(5)-C(18)-C(23)	68.7(4)		
Pd(2)-N(5)-C(18)-C(23)	-131.8(2)		
C(17)-N(5)-C(18)-C(22)	-56.7(4)		
Pd(2)-N(5)-C(18)-C(22)	102.8(2)		
C(21)-N(6)-C(19)-O(4)	-0.7(4)		
Pd(2)-N(6)-C(19)-O(4)	179.9(2)		
C(21)-N(6)-C(19)-C(18)	177.4(3)		
Pd(2)-N(6)-C(19)-C(18)	-2.0(4)		
C(20)-O(4)-C(19)-N(6)	1.6(4)		
C(20)-O(4)-C(19)-C(18)	-176.6(3)		
N(5)-C(18)-C(19)-N(6)	9.9(4)		
C(23)-C(18)-C(19)-N(6)	130.6(3)		
C(22)-C(18)-C(19)-N(6)	-108.0(3)		
N(5)-C(18)-C(19)-O(4)	-172.0(3)		
C(23)-C(18)-C(19)-O(4)	-51.3(4)		
C(22)-C(18)-C(19)-O(4)	70.1(3)		
C(19)-O(4)-C(20)-C(21)	-1.7(3)		
C(19)-N(6)-C(21)-C(24)	-124.6(3)		
Pd(2)-N(6)-C(21)-C(24)	54.5(4)		
C(19)-N(6)-C(21)-C(20)	-0.5(3)		
Pd(2)-N(6)-C(21)-C(20)	178.7(2)		
O(4)-C(20)-C(21)-N(6)	1.3(3)		
O(4)-C(20)-C(21)-C(24)	124.7(3)		
N(6)-C(21)-C(24)-C(29)	-168.1(3)		
C(20)-C(21)-C(24)-C(29)	74.9(4)		
N(6)-C(21)-C(24)-C(25)	12.4(4)		
C(20)-C(21)-C(24)-C(25)	-104.5(3)		
C(29)-C(24)-C(25)-C(26)	0.7(5)		
C(21)-C(24)-C(25)-C(26)	-179.9(3)		
C(24)-C(25)-C(26)-C(27)	-0.1(5)		
C(25)-C(26)-C(27)-C(28)	-0.7(5)		
C(26)-C(27)-C(28)-C(29)	0.9(5)		
C(25)-C(24)-C(29)-C(28)	-0.4(5)		

Figure S4. ORTEP diagram of **32•Zn2Cl2** with H atoms and solvate atoms removed and ellipsoids drawn at the 30% level.

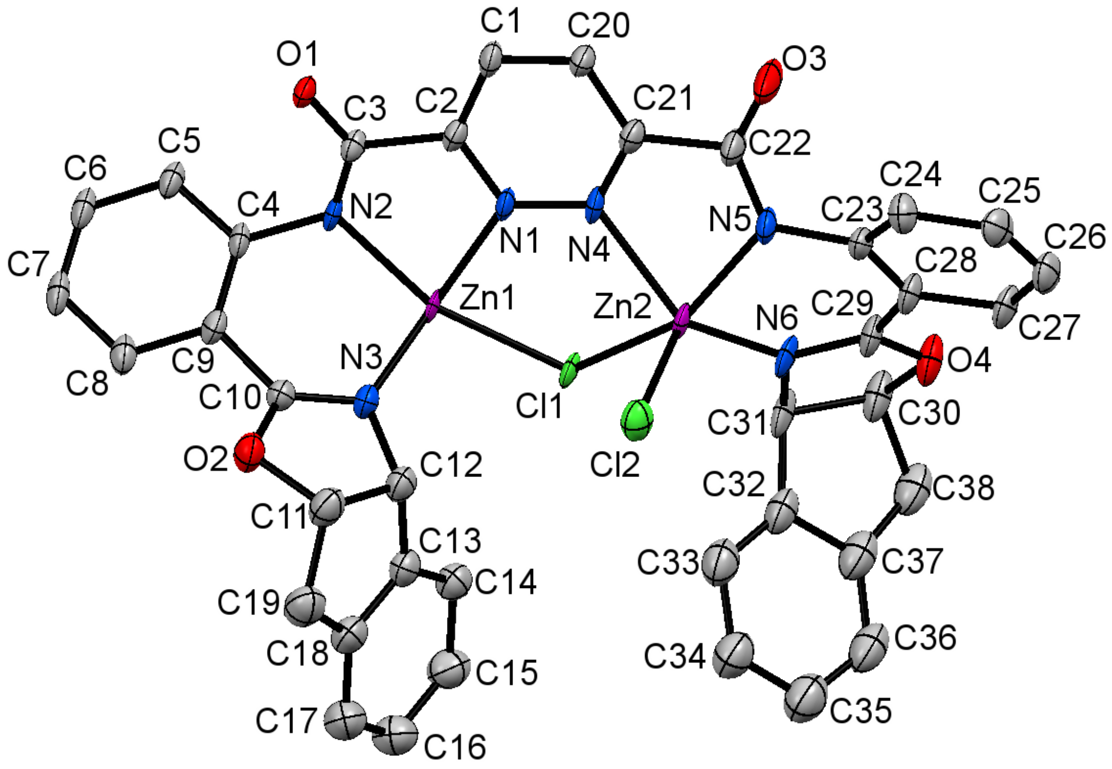

Table S20. Crystal data and structure refinement for **32•Zn2Cl2**.

Identification code	32-Zn2Cl2	
Empirical formula	C42 H38 Cl4 N6 O6 Zn2	
Formula weight	995.32	
Temperature	100(2) K	
Wavelength	0.71073 Å	
Crystal system	Monoclinic	
Space group	C2	
Unit cell dimensions	a = 40.820(5) Å	α= 90°.
b = 7.4084(9) Å	β= 108.589(2)°.	
c = 14.6489(17) Å	γ = 90°.	
Volume	4198.8(9) $Å^3$	
Z	4	
Density (calculated)	1.575 Mg/m^3	
Absorption coefficient	1.453 mm^{-1}	
F(000)	2032	
Crystal size	0.700 x 0.020 x 0.004 mm^3	
Theta range for data collection	2.060 to 25.349°.	
Index ranges	-48<=h<=48, -8<=k<=8, -17<=l<=17	
Reflections collected	17146	
Independent reflections	7576 [R(int) = 0.0836]	
Completeness to theta = 25.242°	99.9 %	
Absorption correction	Semi-empirical from equivalents	
Max. and min. transmission	.9999 and 0.7329	
Refinement method	Full-matrix least-squares on F^2	
Data / restraints / parameters	7576 / 956 / 552	
Goodness-of-fit on F^2	1.158	
Final R indices [I>2sigma(I)]	R1 = 0.0869, wR2 = 0.2009	
R indices (all data)	R1 = 0.1164, wR2 = 0.2165	
Absolute structure parameter	-0.01(3)	
Extinction coefficient	n/a	
Largest diff. peak and hole	1.329 and -0.759 e.$Å^{-3}$	

Table S21. Atomic coordinates (x 10^4) and equivalent isotropic displacement parameters ($Å^2$x 10^3) for **32•Zn2Cl2**. U(eq) is defined as one third of the trace of the orthogonalized U^{ij} tensor.

x	y	z	U(eq)	
Zn(1)	3025(1)	0	5270(1)	21(1)
Zn(2)	3789(1)	-734(3)	7599(1)	23(1)
Cl(1)	3565(1)	1113(5)	6072(2)	23(1)
Cl(2)	3938(1)	-3173(6)	6918(3)	32(1)
N(1)	2994(3)	-1068(16)	6625(8)	20(2)
N(2)	2518(3)	-998(17)	4950(8)	21(2)
N(3)	3093(3)	-1855(18)	4333(8)	25(2)
N(4)	3254(3)	-1108(16)	7461(8)	23(2)
N(5)	3805(3)	-1407(17)	8989(8)	26(2)
N(6)	4156(3)	1156(18)	8169(8)	28(2)
O(1)	2156(2)	-2729(14)	5547(7)	22(2)
O(2)	2910(3)	-4013(15)	3206(7)	31(2)
O(3)	3463(3)	-2330(20)	9896(8)	61(4)
O(4)	4560(3)	2642(15)	9325(8)	37(2)
C(1)	2606(4)	-2290(20)	7419(10)	25(3)
C(2)	2685(3)	-1700(20)	6604(10)	21(2)
C(3)	2419(3)	-1790(20)	5602(9)	20(2)
C(4)	2318(3)	-1218(19)	3950(10)	23(3)
C(5)	1966(3)	-700(20)	3589(10)	26(3)
C(6)	1785(4)	-910(20)	2631(10)	29(3)
C(7)	1932(4)	-1700(20)	2022(10)	31(3)
C(8)	2281(4)	-2280(20)	2364(11)	29(3)
C(9)	2469(4)	-1990(20)	3330(10)	25(3)
C(10)	2841(4)	-2620(20)	3657(10)	24(3)
C(11)	3277(4)	-4540(20)	3695(12)	35(3)
C(12)	3412(4)	-2850(20)	4320(11)	30(3)
C(13)	3619(4)	-1840(20)	3786(11)	32(3)
C(14)	3774(4)	-210(20)	3983(11)	33(3)
C(15)	3968(4)	390(20)	3391(12)	42(3)
C(16)	3991(4)	-600(30)	2638(13)	46(3)
C(17)	3830(5)	-2240(30)	2441(13)	45(3)
C(18)	3639(4)	-2880(30)	3019(12)	38(3)

C(19)	3462(4)	-4670(30)	2961(12)	43(3)
C(20)	2875(3)	-2310(20)	8270(10)	26(3)
C(21)	3200(4)	-1730(20)	8259(10)	26(3)
C(22)	3520(4)	-1860(20)	9159(10)	31(3)
C(23)	4122(4)	-1420(20)	9778(10)	25(3)
C(24)	4186(4)	-2740(20)	10488(11)	31(3)
C(25)	4493(4)	-2630(20)	11280(11)	32(3)
C(26)	4730(4)	-1340(20)	11346(11)	34(3)
C(27)	4681(4)	-70(30)	10636(10)	33(3)
C(28)	4372(3)	-90(20)	9810(10)	28(3)
C(29)	4349(4)	1200(20)	9074(11)	28(3)
C(30)	4484(4)	3880(20)	8500(11)	38(3)
C(31)	4269(4)	2630(20)	7663(11)	34(3)
C(32)	4516(4)	2020(30)	7181(13)	39(3)
C(33)	4483(5)	780(30)	6471(13)	46(3)
C(34)	4755(4)	460(30)	6123(14)	51(4)
C(35)	5059(5)	1410(30)	6458(14)	56(4)
C(36)	5099(5)	2680(30)	7176(14)	49(4)
C(37)	4834(4)	3010(30)	7544(13)	46(3)
C(38)	4810(4)	4370(30)	8261(13)	47(3)
Cl(3A)	4057(4)	-5810(20)	4955(10)	101(4)
Cl(4A)	4800(6)	-3390(30)	4179(15)	137(6)
C(39A)	4382(13)	-4330(70)	5220(40)	125(8)
C(40A)	4688(7)	-4690(80)	4950(40)	132(8)
O(5B)	4361(13)	-4920(80)	5070(40)	122(8)
C(40B)	4475(19)	-5090(110)	4220(40)	128(9)
Cl(5A)	1433(6)	-1970(40)	-726(19)	150
Cl(6A)	2176(6)	-5380(30)	-1168(16)	150
C(41A)	1670(13)	-3800(50)	-630(50)	150
C(42A)	2023(8)	-3770(70)	-630(50)	150
O(6B)	2418(14)	-9610(80)	-240(40)	150
C(41B)	2230(20)	-7980(100)	-650(60)	150
O(7B)	1289(15)	-2290(80)	-540(40)	150
C(42B)	1583(19)	-1130(120)	-510(70)	150
O(8B)	2000(14)	-4300(80)	-1870(40)	150
C(43B)	2150(20)	-3430(120)	-930(50)	150

Table S22. Bond lengths [Å] and angles [°] for **32•Zn2Cl2**.

Zn(1)-N(3)	2.023(12)	C(4)-C(5)	1.419(19)
Zn(1)-O(1)#1	2.060(10)	C(5)-C(6)	1.371(19)
Zn(1)-N(2)	2.105(11)	C(5)-H(5)	0.9500
Zn(1)-N(1)	2.177(11)	C(6)-C(7)	1.36(2)
Zn(1)-Cl(1)	2.297(3)	C(6)-H(6)	0.9500
Zn(2)-N(6)	2.025(12)	C(7)-C(8)	1.41(2)
Zn(2)-N(5)	2.078(12)	C(7)-H(7)	0.9500
Zn(2)-N(4)	2.147(11)	C(8)-C(9)	1.39(2)
Zn(2)-Cl(2)	2.241(4)	C(8)-H(8)	0.9500
Zn(2)-Cl(1)	2.531(4)	C(9)-C(10)	1.52(2)
N(1)-C(2)	1.334(17)	C(11)-C(19)	1.50(2)
N(1)-N(4)	1.341(15)	C(11)-C(12)	1.54(2)
N(2)-C(3)	1.289(17)	C(11)-H(11)	1.0000
N(2)-C(4)	1.440(17)	C(12)-C(13)	1.52(2)
N(3)-C(10)	1.307(18)	C(12)-H(12)	1.0000
N(3)-C(12)	1.503(19)	C(13)-C(14)	1.35(2)
N(4)-C(21)	1.339(18)	C(13)-C(18)	1.39(2)
N(5)-C(22)	1.309(18)	C(14)-C(15)	1.42(2)
N(5)-C(23)	1.433(17)	C(14)-H(14)	0.9500
N(6)-C(29)	1.309(18)	C(15)-C(16)	1.35(2)
N(6)-C(31)	1.47(2)	C(15)-H(15)	0.9500
O(1)-C(3)	1.261(16)	C(16)-C(17)	1.37(3)
O(1)-Zn(1)#2	2.060(10)	C(16)-H(16)	0.9500
O(2)-C(10)	1.303(18)	C(17)-C(18)	1.40(2)
O(2)-C(11)	1.493(18)	C(17)-H(17)	0.9500
O(3)-C(22)	1.226(18)	C(18)-C(19)	1.50(3)
O(4)-C(29)	1.346(18)	C(19)-H(19A)	0.9900
O(4)-C(30)	1.470(19)	C(19)-H(19B)	0.9900
C(1)-C(20)	1.373(19)	C(20)-C(21)	1.40(2)
C(1)-C(2)	1.40(2)	C(20)-H(20)	0.9500
C(1)-H(1)	0.9500	C(21)-C(22)	1.533(19)
C(2)-C(3)	1.524(18)	C(23)-C(24)	1.39(2)
C(4)-C(9)	1.37(2)	C(23)-C(28)	1.41(2)

C(24)-C(25)	1.41(2)	C(39A)-H(39B)	0.9994
C(24)-H(24)	0.9500	C(40A)-H(40A)	1.0483
C(25)-C(26)	1.34(2)	C(40A)-H(40B)	0.9988
C(25)-H(25)	0.9500	O(5B)-C(40B)	1.458(14)
C(26)-C(27)	1.37(2)	O(5B)-H(5B)	0.8667
C(26)-H(26)	0.9500	C(40B)-H(40C)	0.9852
C(27)-C(28)	1.442(18)	C(40B)-H(40D)	0.9779
C(27)-H(27)	0.9500	C(40B)-H(40E)	1.0286
C(28)-C(29)	1.42(2)	Cl(5A)-C(41A)	1.647(14)
C(30)-C(38)	1.52(2)	Cl(6A)-C(42A)	1.659(14)
C(30)-C(31)	1.56(2)	C(41A)-C(42A)	1.440(14)
C(30)-H(30)	1.0000	C(41A)-H(41A)	1.0517
C(31)-C(32)	1.47(2)	C(41A)-H(41B)	0.8824
C(31)-H(31)	1.0000	C(42A)-H(42A)	1.0102
C(32)-C(33)	1.36(3)	C(42A)-H(42B)	1.0542
C(32)-C(37)	1.44(2)	O(6B)-C(41B)	1.443(14)
C(33)-C(34)	1.38(2)	O(6B)-H(6B)	1.0629
C(33)-H(33)	0.9500	C(41B)-H(41C)	1.0054
C(34)-C(35)	1.37(3)	C(41B)-H(41D)	0.9833
C(34)-H(34)	0.9500	C(41B)-H(41E)	0.9706
C(35)-C(36)	1.38(3)	O(7B)-C(42B)	1.462(14)
C(35)-H(35)	0.9500	O(7B)-H(7B)	0.8516
C(36)-C(37)	1.37(3)	C(42B)-H(42C)	1.0113
C(36)-H(36)	0.9500	C(42B)-H(42D)	1.0201
C(37)-C(38)	1.48(3)	C(42B)-H(42E)	0.9997
C(38)-H(38A)	0.9900	O(8B)-C(43B)	1.465(14)
C(38)-H(38B)	0.9900	O(8B)-H(8B)	0.8358
Cl(3A)-C(39A)	1.666(14)	C(43B)-H(43C)	0.9711
Cl(4A)-C(40A)	1.652(14)	C(43B)-H(43D)	1.0769
C(39A)-C(40A)	1.449(14)	C(43B)-H(43E)	1.0428
C(39A)-H(39A)	0.9520		

N(3)-Zn(1)-O(1)#1	105.2(4)	O(1)#1-Zn(1)-N(2)	90.7(4)
N(3)-Zn(1)-N(2)	87.0(5)	N(3)-Zn(1)-N(1)	115.6(5)

O(1)#1-Zn(1)-N(1)	135.4(4)	C(10)-O(2)-C(11)	108.0(11)
N(2)-Zn(1)-N(1)	74.7(4)	C(29)-O(4)-C(30)	108.8(11)
N(3)-Zn(1)-Cl(1)	105.9(3)	C(20)-C(1)-C(2)	116.2(13)
O(1)#1-Zn(1)-Cl(1)	96.4(3)	C(20)-C(1)-H(1)	121.9
N(2)-Zn(1)-Cl(1)	162.9(3)	C(2)-C(1)-H(1)	121.9
N(1)-Zn(1)-Cl(1)	89.4(3)	N(1)-C(2)-C(1)	124.2(12)
N(6)-Zn(2)-N(5)	88.5(5)	N(1)-C(2)-C(3)	114.5(12)
N(6)-Zn(2)-N(4)	136.4(5)	C(1)-C(2)-C(3)	121.3(12)
N(5)-Zn(2)-N(4)	77.0(4)	O(1)-C(3)-N(2)	131.5(12)
N(6)-Zn(2)-Cl(2)	118.1(4)	O(1)-C(3)-C(2)	114.6(12)
N(5)-Zn(2)-Cl(2)	108.3(4)	N(2)-C(3)-C(2)	113.5(12)
N(4)-Zn(2)-Cl(2)	105.5(3)	C(9)-C(4)-C(5)	118.7(13)
N(6)-Zn(2)-Cl(1)	90.1(3)	C(9)-C(4)-N(2)	119.5(12)
N(5)-Zn(2)-Cl(1)	152.4(4)	C(5)-C(4)-N(2)	121.8(13)
N(4)-Zn(2)-Cl(1)	85.2(3)	C(6)-C(5)-C(4)	120.1(14)
Cl(2)-Zn(2)-Cl(1)	96.61(14)	C(6)-C(5)-H(5)	119.9
Zn(1)-Cl(1)-Zn(2)	105.97(14)	C(4)-C(5)-H(5)	119.9
C(2)-N(1)-N(4)	118.6(11)	C(7)-C(6)-C(5)	121.0(14)
C(2)-N(1)-Zn(1)	115.8(9)	C(7)-C(6)-H(6)	119.5
N(4)-N(1)-Zn(1)	125.5(8)	C(5)-C(6)-H(6)	119.5
C(3)-N(2)-C(4)	119.6(11)	C(6)-C(7)-C(8)	120.2(13)
C(3)-N(2)-Zn(1)	120.7(9)	C(6)-C(7)-H(7)	119.9
C(4)-N(2)-Zn(1)	117.6(9)	C(8)-C(7)-H(7)	119.9
C(10)-N(3)-C(12)	104.7(12)	C(9)-C(8)-C(7)	118.7(14)
C(10)-N(3)-Zn(1)	124.3(10)	C(9)-C(8)-H(8)	120.7
C(12)-N(3)-Zn(1)	130.5(9)	C(7)-C(8)-H(8)	120.7
C(21)-N(4)-N(1)	120.2(11)	C(4)-C(9)-C(8)	121.2(13)
C(21)-N(4)-Zn(2)	114.0(9)	C(4)-C(9)-C(10)	122.3(12)
N(1)-N(4)-Zn(2)	124.6(9)	C(8)-C(9)-C(10)	116.5(13)
C(22)-N(5)-C(23)	118.3(12)	O(2)-C(10)-N(3)	118.9(13)
C(22)-N(5)-Zn(2)	119.6(9)	O(2)-C(10)-C(9)	116.1(12)
C(23)-N(5)-Zn(2)	122.0(9)	N(3)-C(10)-C(9)	125.0(13)
C(29)-N(6)-C(31)	107.8(12)	O(2)-C(11)-C(19)	109.3(13)
C(29)-N(6)-Zn(2)	124.3(11)	O(2)-C(11)-C(12)	100.7(12)
C(31)-N(6)-Zn(2)	127.8(9)	C(19)-C(11)-C(12)	108.1(14)
C(3)-O(1)-Zn(1)#2	129.1(9)	O(2)-C(11)-H(11)	112.7

C(19)-C(11)-H(11)	112.7	N(4)-C(21)-C(22)	116.1(12)
C(12)-C(11)-H(11)	112.7	C(20)-C(21)-C(22)	121.6(13)
N(3)-C(12)-C(13)	113.1(13)	O(3)-C(22)-N(5)	132.0(14)
N(3)-C(12)-C(11)	105.0(12)	O(3)-C(22)-C(21)	115.2(13)
C(13)-C(12)-C(11)	104.3(13)	N(5)-C(22)-C(21)	112.8(12)
N(3)-C(12)-H(12)	111.4	C(24)-C(23)-C(28)	120.4(13)
C(13)-C(12)-H(12)	111.4	C(24)-C(23)-N(5)	120.8(14)
C(11)-C(12)-H(12)	111.4	C(28)-C(23)-N(5)	118.7(13)
C(14)-C(13)-C(18)	121.7(15)	C(23)-C(24)-C(25)	118.6(15)
C(14)-C(13)-C(12)	129.0(15)	C(23)-C(24)-H(24)	120.7
C(18)-C(13)-C(12)	109.3(15)	C(25)-C(24)-H(24)	120.7
C(13)-C(14)-C(15)	117.6(15)	C(26)-C(25)-C(24)	122.0(15)
C(13)-C(14)-H(14)	121.2	C(26)-C(25)-H(25)	119.0
C(15)-C(14)-H(14)	121.2	C(24)-C(25)-H(25)	119.0
C(16)-C(15)-C(14)	121.9(16)	C(25)-C(26)-C(27)	120.6(15)
C(16)-C(15)-H(15)	119.1	C(25)-C(26)-H(26)	119.7
C(14)-C(15)-H(15)	119.1	C(27)-C(26)-H(26)	119.7
C(15)-C(16)-C(17)	119.8(16)	C(26)-C(27)-C(28)	120.6(15)
C(15)-C(16)-H(16)	120.1	C(26)-C(27)-H(27)	119.7
C(17)-C(16)-H(16)	120.1	C(28)-C(27)-H(27)	119.7
C(16)-C(17)-C(18)	119.8(17)	C(23)-C(28)-C(29)	125.0(12)
C(16)-C(17)-H(17)	120.1	C(23)-C(28)-C(27)	117.6(14)
C(18)-C(17)-H(17)	120.1	C(29)-C(28)-C(27)	117.4(14)
C(13)-C(18)-C(17)	119.3(17)	N(6)-C(29)-O(4)	115.1(14)
C(13)-C(18)-C(19)	112.6(14)	N(6)-C(29)-C(28)	128.5(14)
C(17)-C(18)-C(19)	128.0(16)	O(4)-C(29)-C(28)	116.4(12)
C(11)-C(19)-C(18)	104.3(13)	O(4)-C(30)-C(38)	111.5(13)
C(11)-C(19)-H(19A)	110.9	O(4)-C(30)-C(31)	101.1(12)
C(18)-C(19)-H(19A)	110.9	C(38)-C(30)-C(31)	105.8(14)
C(11)-C(19)-H(19B)	110.9	O(4)-C(30)-H(30)	112.6
C(18)-C(19)-H(19B)	110.9	C(38)-C(30)-H(30)	112.6
H(19A)-C(19)-H(19B)	108.9	C(31)-C(30)-H(30)	112.6
C(1)-C(20)-C(21)	118.2(14)	C(32)-C(31)-N(6)	112.7(14)
C(1)-C(20)-H(20)	120.9	C(32)-C(31)-C(30)	105.0(13)
C(21)-C(20)-H(20)	120.9	N(6)-C(31)-C(30)	103.4(12)
N(4)-C(21)-C(20)	122.3(13)	C(32)-C(31)-H(31)	111.7

N(6)-C(31)-H(31)	111.7	Cl(4A)-C(40A)-H(40B)	108.3
C(30)-C(31)-H(31)	111.7	H(40A)-C(40A)-H(40B)	101.9
C(33)-C(32)-C(37)	119.0(17)	C(40B)-O(5B)-H(5B)	105.2
C(33)-C(32)-C(31)	130.9(16)	O(5B)-C(40B)-H(40C)	113.2
C(37)-C(32)-C(31)	110.0(16)	O(5B)-C(40B)-H(40D)	114.2
C(32)-C(33)-C(34)	120.0(18)	H(40C)-C(40B)-H(40D)	108.0
C(32)-C(33)-H(33)	120.0	O(5B)-C(40B)-H(40E)	111.8
C(34)-C(33)-H(33)	120.0	H(40C)-C(40B)-H(40E)	104.1
C(35)-C(34)-C(33)	121(2)	H(40D)-C(40B)-H(40E)	104.7
C(35)-C(34)-H(34)	119.3	C(42A)-C(41A)-Cl(5A)	123.1(17)
C(33)-C(34)-H(34)	119.3	C(42A)-C(41A)-H(41A)	98.4
C(34)-C(35)-C(36)	119.6(19)	Cl(5A)-C(41A)-H(41A)	103.9
C(34)-C(35)-H(35)	120.2	C(42A)-C(41A)-H(41B)	107.2
C(36)-C(35)-H(35)	120.2	Cl(5A)-C(41A)-H(41B)	112.7
C(37)-C(36)-C(35)	120.3(18)	H(41A)-C(41A)-H(41B)	109.9
C(37)-C(36)-H(36)	119.9	C(41A)-C(42A)-Cl(6A)	121.0(17)
C(35)-C(36)-H(36)	119.9	C(41A)-C(42A)-H(42A)	114.4
C(36)-C(37)-C(32)	119.6(18)	Cl(6A)-C(42A)-H(42A)	107.1
C(36)-C(37)-C(38)	129.7(17)	C(41A)-C(42A)-H(42B)	107.2
C(32)-C(37)-C(38)	110.5(16)	Cl(6A)-C(42A)-H(42B)	104.0
C(37)-C(38)-C(30)	105.3(15)	H(42A)-C(42A)-H(42B)	100.7
C(37)-C(38)-H(38A)	110.7	C(41B)-O(6B)-H(6B)	110.5
C(30)-C(38)-H(38A)	110.7	O(6B)-C(41B)-H(41C)	111.3
C(37)-C(38)-H(38B)	110.7	O(6B)-C(41B)-H(41D)	111.6
C(30)-C(38)-H(38B)	110.7	H(41C)-C(41B)-H(41D)	105.9
H(38A)-C(38)-H(38B)	108.8	O(6B)-C(41B)-H(41E)	112.3
C(40A)-C(39A)-Cl(3A)	120.6(17)	H(41C)-C(41B)-H(41E)	106.9
C(40A)-C(39A)-H(39A)	105.5	H(41D)-C(41B)-H(41E)	108.6
Cl(3A)-C(39A)-H(39A)	109.8	C(42B)-O(7B)-H(7B)	101.6
C(40A)-C(39A)-H(39B)	104.0	O(7B)-C(42B)-H(42C)	115.1
Cl(3A)-C(39A)-H(39B)	107.2	O(7B)-C(42B)-H(42D)	115.3
H(39A)-C(39A)-H(39B)	109.3	H(42C)-C(42B)-H(42D)	103.1
C(39A)-C(40A)-Cl(4A)	122.2(17)	O(7B)-C(42B)-H(42E)	114.6
C(39A)-C(40A)-H(40A)	107.2	H(42C)-C(42B)-H(42E)	103.3
Cl(4A)-C(40A)-H(40A)	104.9	H(42D)-C(42B)-H(42E)	103.9
C(39A)-C(40A)-H(40B)	110.3	C(43B)-O(8B)-H(8B)	97.2

O(8B)-C(43B)-H(43C)	119.7	O(8B)-C(43B)-H(43E)	116.7
O(8B)-C(43B)-H(43D)	114.1	H(43C)-C(43B)-H(43E)	104.2
H(43C)-C(43B)-H(43D)	101.8	H(43D)-C(43B)-H(43E)	97.1

Symmetry transformations used to generate equivalent atoms:

#1 -x+1/2,y+1/2,-z+1 #2 -x+1/2,y-1/2,-z+1

Table S23. Anisotropic displacement parameters ($Å^2$x 10^3) for **32•Zn2Cl2**. The anisotropic displacement factor exponent takes the form: $-2\pi^2[h^2 a^{*2}U^{11} + ... + 2 h k a^* b^* U^{12}]$

	U^{11}	U^{22}	U^{33}	U^{23}	U^{13}	U^{12}
Zn(1)	6(1)	31(1)	18(1)	-1(1)	-6(1)	0(1)
Zn(2)	9(1)	31(1)	20(1)	0(1)	-6(1)	0(1)
Cl(1)	9(2)	31(2)	22(2)	2(2)	-6(1)	-1(1)
Cl(2)	27(2)	32(2)	31(2)	-1(2)	0(2)	8(2)
N(1)	11(4)	26(6)	17(4)	-2(4)	-4(3)	0(4)
N(2)	9(4)	28(6)	20(4)	-2(4)	-5(3)	-3(4)
N(3)	15(5)	35(6)	21(5)	-2(4)	0(4)	-1(4)
N(4)	12(4)	28(6)	20(4)	-2(4)	-6(4)	-2(4)
N(5)	16(4)	38(6)	17(4)	1(4)	-5(4)	-4(4)
N(6)	12(5)	34(5)	27(5)	7(4)	-9(4)	-1(4)
O(1)	11(4)	32(5)	20(5)	1(4)	-1(4)	-1(4)
O(2)	24(4)	33(5)	31(5)	-6(4)	1(4)	-1(4)
O(3)	21(6)	128(12)	26(6)	16(7)	-5(5)	-15(7)
O(4)	25(5)	36(5)	35(5)	4(4)	-12(4)	-6(4)
C(1)	15(5)	30(6)	23(5)	-4(5)	-3(4)	-1(5)
C(2)	12(5)	23(5)	24(5)	-2(5)	0(4)	0(4)
C(3)	14(5)	22(5)	20(5)	3(5)	0(4)	0(4)
C(4)	15(5)	27(6)	19(5)	-1(4)	-4(4)	-5(4)
C(5)	15(5)	32(6)	25(5)	3(5)	-2(4)	-3(5)
C(6)	17(5)	34(6)	26(5)	5(5)	-6(4)	-6(5)
C(7)	22(6)	34(6)	24(6)	3(5)	-8(5)	-9(5)
C(8)	23(5)	35(6)	23(5)	-1(5)	-2(5)	-6(5)
C(9)	15(5)	32(6)	25(5)	-3(5)	0(4)	-5(5)
C(10)	18(5)	30(6)	21(5)	-2(4)	4(4)	1(4)
C(11)	24(5)	38(6)	41(6)	-5(5)	6(5)	3(4)
C(12)	20(5)	36(6)	29(5)	-3(5)	3(4)	2(4)
C(13)	22(5)	41(6)	28(5)	1(5)	3(4)	3(5)
C(14)	27(6)	38(6)	33(6)	-1(5)	7(5)	3(5)
C(15)	36(6)	47(7)	46(6)	2(6)	16(5)	1(6)
C(16)	41(7)	54(7)	46(6)	-1(6)	19(6)	2(6)
C(17)	37(7)	59(7)	41(6)	-4(6)	14(5)	1(6)

C(18)	25(6)	51(6)	36(6)	-4(5)	7(5)	2(5)
C(19)	34(6)	46(7)	48(6)	-11(6)	12(5)	3(5)
C(20)	14(5)	39(7)	21(5)	1(5)	1(4)	-2(5)
C(21)	14(4)	35(6)	24(5)	2(5)	0(4)	2(5)
C(22)	16(5)	48(7)	23(5)	5(5)	-3(4)	-3(5)
C(23)	15(5)	39(6)	18(5)	0(4)	0(4)	3(4)
C(24)	27(6)	39(7)	23(6)	1(5)	0(5)	1(5)
C(25)	26(6)	41(7)	24(6)	8(5)	1(5)	14(5)
C(26)	23(6)	46(7)	26(6)	1(5)	-4(5)	7(5)
C(27)	16(5)	43(6)	31(6)	3(5)	-7(5)	3(5)
C(28)	15(5)	35(6)	26(5)	2(5)	-6(4)	1(5)
C(29)	15(5)	33(6)	27(5)	-1(5)	-7(4)	-3(5)
C(30)	25(5)	35(6)	39(6)	5(5)	-9(5)	-5(5)
C(31)	19(5)	37(6)	32(5)	8(5)	-12(4)	-3(5)
C(32)	24(5)	45(6)	40(6)	5(5)	-2(5)	-3(5)
C(33)	34(6)	55(7)	41(7)	3(6)	2(5)	-2(6)
C(34)	34(6)	62(8)	52(7)	1(6)	7(6)	0(6)
C(35)	38(6)	67(8)	57(7)	3(6)	8(6)	0(6)
C(36)	30(6)	58(7)	54(7)	9(6)	3(6)	-7(6)
C(37)	31(6)	50(7)	51(6)	8(5)	3(5)	-7(5)
C(38)	32(6)	47(7)	52(7)	7(6)	1(5)	-10(6)
Cl(3A)	111(9)	75(8)	90(8)	-3(7)	-9(7)	19(7)
Cl(4A)	150(12)	128(12)	136(12)	-5(10)	48(10)	1(11)
C(39A)	125(10)	118(12)	126(11)	3(10)	33(8)	3(9)
C(40A)	132(10)	127(12)	133(11)	2(9)	36(9)	5(9)
O(5B)	117(13)	122(14)	127(13)	16(13)	42(11)	8(13)
C(40B)	134(14)	126(15)	123(14)	1(14)	39(12)	7(13)

Table S24. Hydrogen coordinates (x 10^4) and isotropic displacement parameters ($Å^2$x 10^3) for **32•Zn2Cl2**.

x	y	z	U(eq)	
H(1)	2379	-2648	7385	30
H(5)	1855	-200	4012	31
H(6)	1553	-493	2389	34
H(7)	1801	-1871	1364	37
H(8)	2384	-2848	1944	35
H(11)	3299	-5665	4087	42
H(12)	3559	-3190	4985	35
H(14)	3753	508	4498	40
H(15)	4086	1510	3529	51
H(16)	4119	-155	2246	55
H(17)	3847	-2941	1916	54
H(19A)	3296	-4871	2309	52
H(19B)	3632	-5667	3118	52
H(20)	2841	-2699	8850	31
H(24)	4027	-3701	10441	38
H(25)	4534	-3502	11782	38
H(26)	4933	-1303	11890	41
H(27)	4852	825	10687	40
H(30)	4352	4971	8584	45
H(31)	4068	3282	7209	41
H(33)	4273	126	6216	55
H(34)	4731	-436	5641	62
H(35)	5241	1191	6197	67
H(36)	5310	3323	7417	59
H(38A)	4792	5600	7987	56
H(38B)	5015	4311	8845	56
H(39A)	4300	-3187	4951	158
H(39B)	4473	-4233	5937	158
H(40A)	4659	-5980	4640	158
H(40B)	4894	-4849	5532	158

H(5B)	4542	-5149	5555	192
H(40C)	4457	-3955	3861	192
H(40D)	4356	-6032	3773	192
H(40E)	4733	-5426	4412	192
H(41A)	1714	-4229	82	180
H(41B)	1563	-4662	-1035	180
H(42A)	2199	-3578	27	180
H(42B)	2056	-2581	-983	180
H(6B)	2688	-9369	6	225
H(41C)	2358	-6877	-315	225
H(41D)	2214	-7845	-1331	225
H(41E)	2003	-7948	-591	225
H(7B)	1166	-1584	-329	225
H(42C)	1811	-1523	-33	225
H(42D)	1562	178	-322	225
H(42E)	1635	-1043	-1129	225
H(8B)	1798	-4389	-1855	225
H(43C)	2114	-2138	-865	225
H(43D)	2423	-3553	-626	225
H(43E)	2085	-3993	-352	225

Table S25. Torsion angles [°] for **32•Zn2Cl2**.

Symmetry transformations used to generate equivalent atoms: #1 -x+1/2,y+1/2,-z+1 #2 -x+1/2,y-1/2,-z+1

C(2)-N(1)-N(4)-C(21)	-1(2)	C(7)-C(8)-C(9)-C(4)	2(2)
Zn(1)-N(1)-N(4)-C(21)	177.4(11)	C(7)-C(8)-C(9)-C(10)	-179.6(14)
C(2)-N(1)-N(4)-Zn(2)	165.7(10)	C(11)-O(2)-C(10)-N(3)	7.0(18)
Zn(1)-N(1)-N(4)-Zn(2)	-15.6(15)	C(11)-O(2)-C(10)-C(9)	-174.2(13)
N(4)-N(1)-C(2)-C(1)	5(2)	C(12)-N(3)-C(10)-O(2)	4.1(18)
Zn(1)-N(1)-C(2)-C(1)	-174.0(11)	Zn(1)-N(3)-C(10)-O(2)	-168.1(10)
N(4)-N(1)-C(2)-C(3)	-173.6(12)	C(12)-N(3)-C(10)-C(9)	-174.6(14)
Zn(1)-N(1)-C(2)-C(3)	7.6(15)	Zn(1)-N(3)-C(10)-C(9)	13(2)
C(20)-C(1)-C(2)-N(1)	-4(2)	C(4)-C(9)-C(10)-O(2)	147.9(14)
C(20)-C(1)-C(2)-C(3)	173.9(14)	C(8)-C(9)-C(10)-O(2)	-30(2)
Zn(1)#2-O(1)-C(3)-N(2)	54(2)	C(4)-C(9)-C(10)-N(3)	-33(2)
Zn(1)#2-O(1)-C(3)-C(2)	-119.3(11)	C(8)-C(9)-C(10)-N(3)	148.5(15)
C(4)-N(2)-C(3)-O(1)	-3(2)	C(10)-O(2)-C(11)-C(19)	-127.7(14)
Zn(1)-N(2)-C(3)-O(1)	-166.6(12)	C(10)-O(2)-C(11)-C(12)	-14.1(15)
C(4)-N(2)-C(3)-C(2)	170.1(12)	C(10)-N(3)-C(12)-C(13)	100.2(15)
Zn(1)-N(2)-C(3)-C(2)	6.9(17)	Zn(1)-N(3)-C(12)-C(13)	-88.3(15)
N(1)-C(2)-C(3)-O(1)	165.2(12)	C(10)-N(3)-C(12)-C(11)	-12.9(16)
C(1)-C(2)-C(3)-O(1)	-13(2)	Zn(1)-N(3)-C(12)-C(11)	158.6(11)
N(1)-C(2)-C(3)-N(2)	-9.4(18)	O(2)-C(11)-C(12)-N(3)	15.9(15)
C(1)-C(2)-C(3)-N(2)	172.1(14)	C(19)-C(11)-C(12)-N(3)	130.5(13)
C(3)-N(2)-C(4)-C(9)	-119.4(15)	O(2)-C(11)-C(12)-C(13)	-103.2(13)
Zn(1)-N(2)-C(4)-C(9)	44.4(17)	C(19)-C(11)-C(12)-C(13)	11.3(17)
C(3)-N(2)-C(4)-C(5)	59.2(19)	N(3)-C(12)-C(13)-C(14)	61(2)
Zn(1)-N(2)-C(4)-C(5)	-137.0(12)	C(11)-C(12)-C(13)-C(14)	174.9(15)
C(9)-C(4)-C(5)-C(6)	-2(2)	N(3)-C(12)-C(13)-C(18)	-119.5(15)
N(2)-C(4)-C(5)-C(6)	179.3(14)	C(11)-C(12)-C(13)-C(18)	-6.1(17)
C(4)-C(5)-C(6)-C(7)	3(2)	C(18)-C(13)-C(14)-C(15)	-2(2)
C(5)-C(6)-C(7)-C(8)	-2(2)	C(12)-C(13)-C(14)-C(15)	176.8(16)
C(6)-C(7)-C(8)-C(9)	-1(2)	C(13)-C(14)-C(15)-C(16)	2(2)
C(5)-C(4)-C(9)-C(8)	-1(2)	C(14)-C(15)-C(16)-C(17)	-1(3)
N(2)-C(4)-C(9)-C(8)	177.9(14)	C(15)-C(16)-C(17)-C(18)	1(3)
C(5)-C(4)-C(9)-C(10)	-178.8(14)	C(14)-C(13)-C(18)-C(17)	1(2)
N(2)-C(4)-C(9)-C(10)	0(2)	C(12)-C(13)-C(18)-C(17)	-177.8(15)

C(14)-C(13)-C(18)-C(19)	177.6(15)	C(26)-C(27)-C(28)-C(23)	2(2)
C(12)-C(13)-C(18)-C(19)	-1.5(19)	C(26)-C(27)-C(28)-C(29)	-176.3(15)
C(16)-C(17)-C(18)-C(13)	0(3)	C(31)-N(6)-C(29)-O(4)	7.8(18)
C(16)-C(17)-C(18)-C(19)	-176.1(18)	Zn(2)-N(6)-C(29)-O(4)	-175.4(10)
O(2)-C(11)-C(19)-C(18)	96.7(15)	C(31)-N(6)-C(29)-C(28)	-170.0(16)
C(12)-C(11)-C(19)-C(18)	-12.1(17)	Zn(2)-N(6)-C(29)-C(28)	7(2)
C(13)-C(18)-C(19)-C(11)	8.6(19)	C(30)-O(4)-C(29)-N(6)	5.8(18)
C(17)-C(18)-C(19)-C(11)	-175.5(17)	C(30)-O(4)-C(29)-C(28)	-176.1(14)
C(2)-C(1)-C(20)-C(21)	1(2)	C(23)-C(28)-C(29)-N(6)	-19(3)
N(1)-N(4)-C(21)-C(20)	-2(2)	C(27)-C(28)-C(29)-N(6)	158.6(16)
Zn(2)-N(4)-C(21)-C(20)	-170.7(12)	C(23)-C(28)-C(29)-O(4)	163.1(14)
N(1)-N(4)-C(21)-C(22)	175.5(13)	C(27)-C(28)-C(29)-O(4)	-19(2)
Zn(2)-N(4)-C(21)-C(22)	7.2(18)	C(29)-O(4)-C(30)-C(38)	-127.5(15)
C(1)-C(20)-C(21)-N(4)	3(2)	C(29)-O(4)-C(30)-C(31)	-15.5(16)
C(1)-C(20)-C(21)-C(22)	-175.1(14)	C(29)-N(6)-C(31)-C(32)	96.0(15)
C(23)-N(5)-C(22)-O(3)	-1(3)	Zn(2)-N(6)-C(31)-C(32)	-80.7(15)
Zn(2)-N(5)-C(22)-O(3)	178.6(18)	C(29)-N(6)-C(31)-C(30)	-16.9(16)
C(23)-N(5)-C(22)-C(21)	178.4(14)	Zn(2)-N(6)-C(31)-C(30)	166.4(10)
Zn(2)-N(5)-C(22)-C(21)	-2.3(19)	O(4)-C(30)-C(31)-C(32)	-99.4(14)
N(4)-C(21)-C(22)-O(3)	175.8(16)	C(38)-C(30)-C(31)-C(32)	16.9(18)
C(20)-C(21)-C(22)-O(3)	-6(3)	O(4)-C(30)-C(31)-N(6)	19.0(15)
N(4)-C(21)-C(22)-N(5)	-3(2)	C(38)-C(30)-C(31)-N(6)	135.3(14)
C(20)-C(21)-C(22)-N(5)	174.4(15)	N(6)-C(31)-C(32)-C(33)	61(2)
C(22)-N(5)-C(23)-C(24)	38(2)	C(30)-C(31)-C(32)-C(33)	172.6(19)
Zn(2)-N(5)-C(23)-C(24)	-141.5(12)	N(6)-C(31)-C(32)-C(37)	-121.3(16)
C(22)-N(5)-C(23)-C(28)	-143.8(15)	C(30)-C(31)-C(32)-C(37)	-9.4(19)
Zn(2)-N(5)-C(23)-C(28)	37.0(18)	C(37)-C(32)-C(33)-C(34)	1(3)
C(28)-C(23)-C(24)-C(25)	5(2)	C(31)-C(32)-C(33)-C(34)	179.0(18)
N(5)-C(23)-C(24)-C(25)	-176.3(14)	C(32)-C(33)-C(34)-C(35)	-2(3)
C(23)-C(24)-C(25)-C(26)	-3(2)	C(33)-C(34)-C(35)-C(36)	2(3)
C(24)-C(25)-C(26)-C(27)	0(3)	C(34)-C(35)-C(36)-C(37)	-1(3)
C(25)-C(26)-C(27)-C(28)	1(2)	C(35)-C(36)-C(37)-C(32)	0(3)
C(24)-C(23)-C(28)-C(29)	173.0(15)	C(35)-C(36)-C(37)-C(38)	-175.7(19)
N(5)-C(23)-C(28)-C(29)	-5(2)	C(33)-C(32)-C(37)-C(36)	0(3)
C(24)-C(23)-C(28)-C(27)	-5(2)	C(31)-C(32)-C(37)-C(36)	-178.7(16)
N(5)-C(23)-C(28)-C(27)	176.9(13)	C(33)-C(32)-C(37)-C(38)	176.3(16)

C(31)-C(32)-C(37)-C(38)	-2(2)
C(36)-C(37)-C(38)-C(30)	-170.9(19)
C(32)-C(37)-C(38)-C(30)	13(2)
O(4)-C(30)-C(38)-C(37)	91.1(16)
C(31)-C(30)-C(38)-C(37)	-18.0(18)
Cl(3A)-C(39A)-C(40A)-Cl(4A)	117(4)
Cl(5A)-C(41A)-C(42A)-Cl(6A)	-143(5)

Table S26. Hydrogen bonds for **32•Zn2Cl2** [Å and °].

D-H...A	d(D-H)	d(H...A)	d(D...A)	<(DHA)
C(1)-H(1)...O(8B)#3	0.95	2.49	3.33(6)	148.1
C(11)-H(11)...O(1)#2	1.00	2.59	3.346(19)	132.3
C(12)-H(12)...Cl(2)	1.00	2.76	3.734(16)	164.7
C(12)-H(12)...Cl(3A)	1.00	2.82	3.32(2)	111.7
C(19)-H(19B)...Cl(3A)	0.99	2.70	3.26(2)	116.2
C(30)-H(30)...Cl(2)#4	1.00	2.84	3.435(16)	118.8
C(31)-H(31)...Cl(1)	1.00	2.72	3.268(15)	114.9
C(31)-H(31)...Cl(2)#4	1.00	2.69	3.427(17)	131.0
C(40A)-H(40B)...Cl(4A)#5	1.00	1.61	2.29(5)	121.2
C(41A)-H(41A)...O(3)#2	1.05	2.41	2.94(4)	110.0

Symmetry transformations used to generate equivalent atoms:

#1 -x+1/2,y+1/2,-z+1 #2 -x+1/2,y-1/2,-z+1 #3 x,y,z+1

#4 x,y+1,z #5 -x+1,y,-z+1

^1H NMR of **16**-H_2 in $CDCl_3$

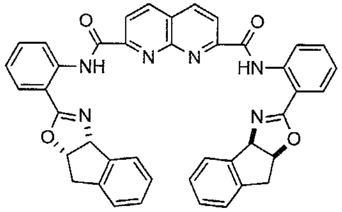

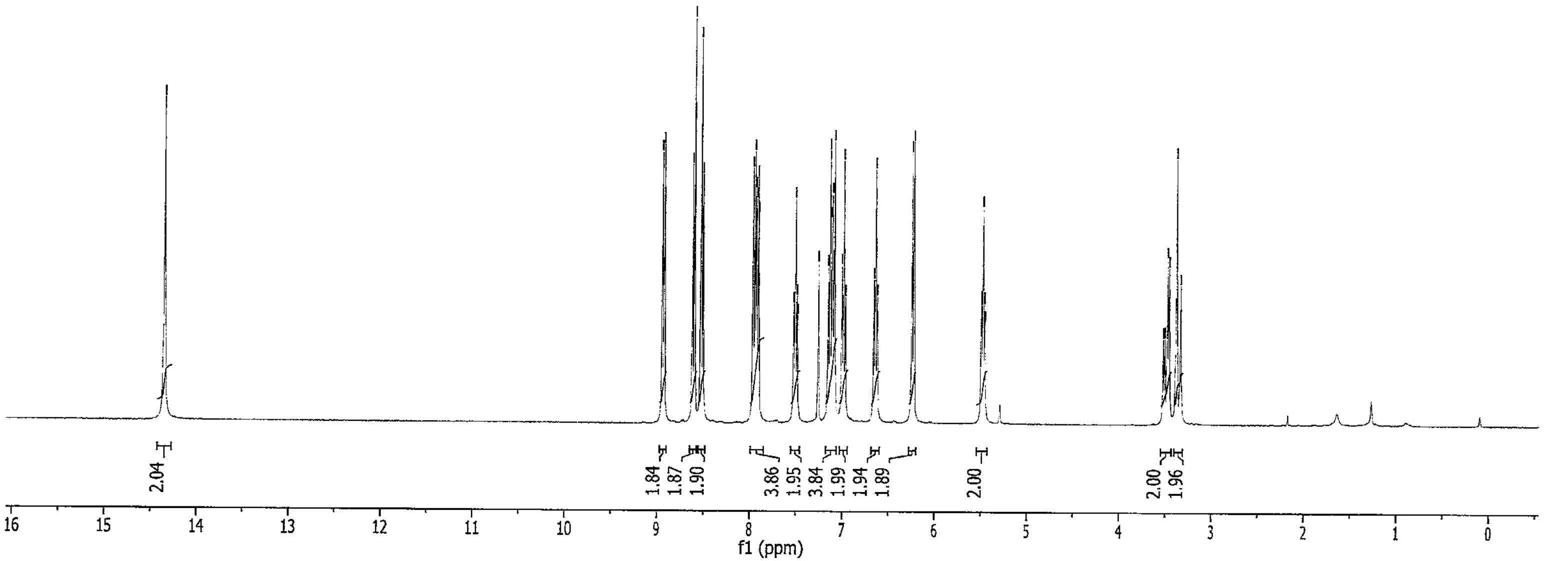

^{13}C NMR of **16**-H_2 in $CDCl_3$

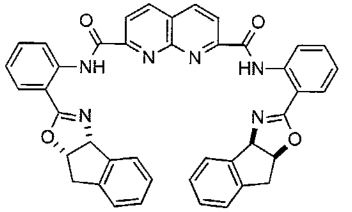

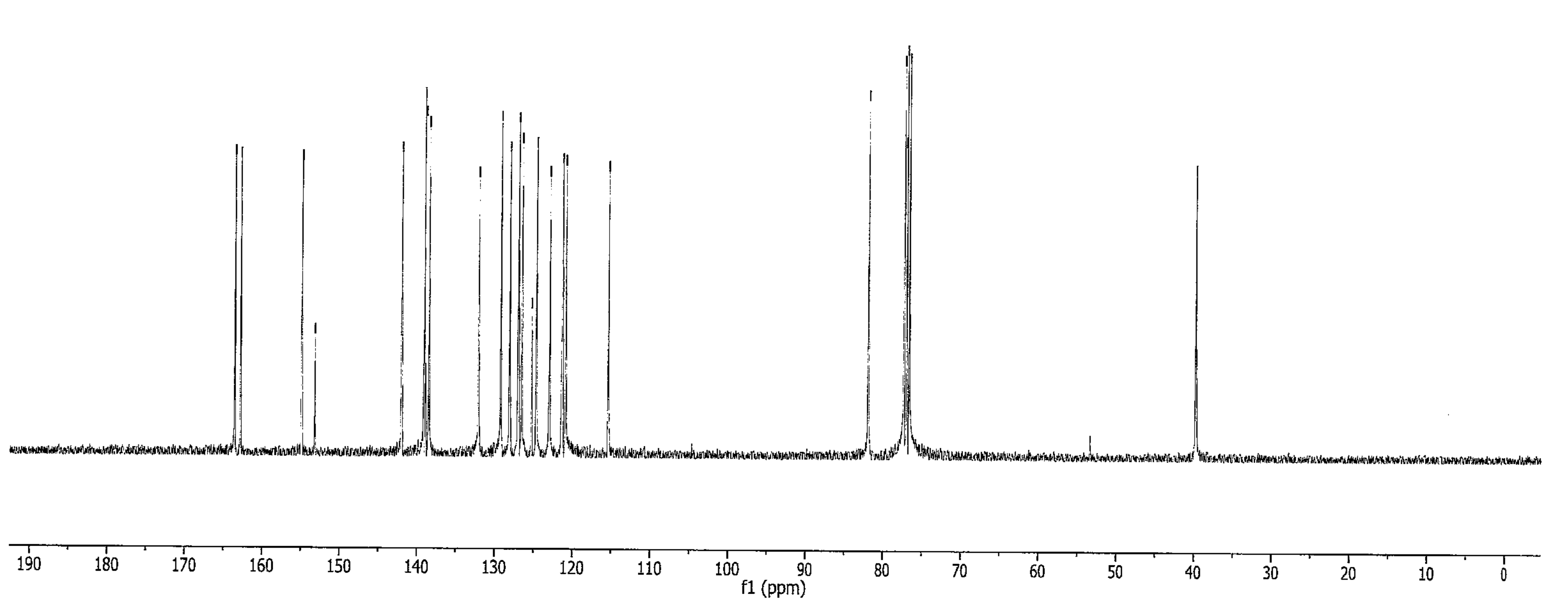

^{1}H NMR of **19** in $CDCl_3$

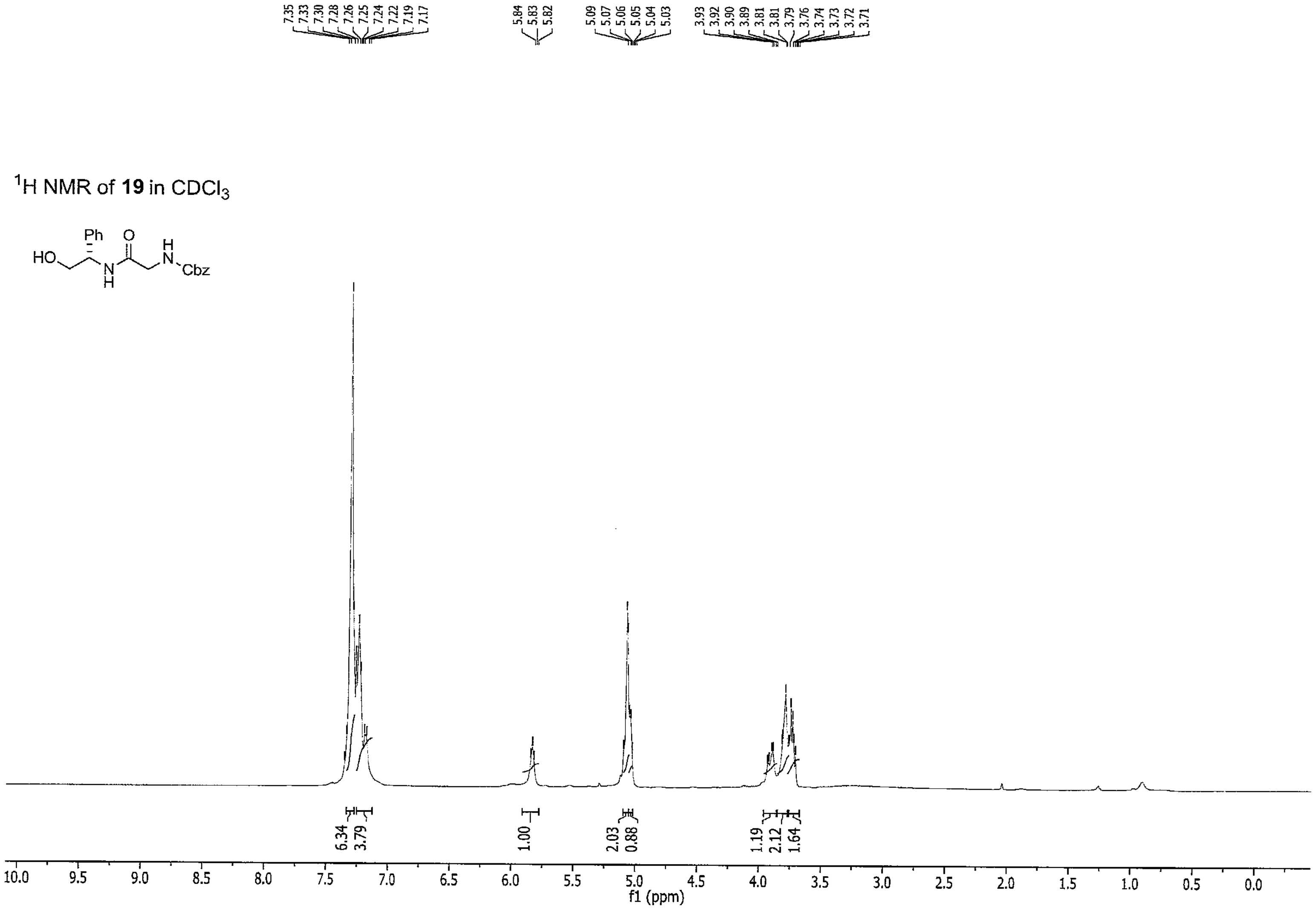

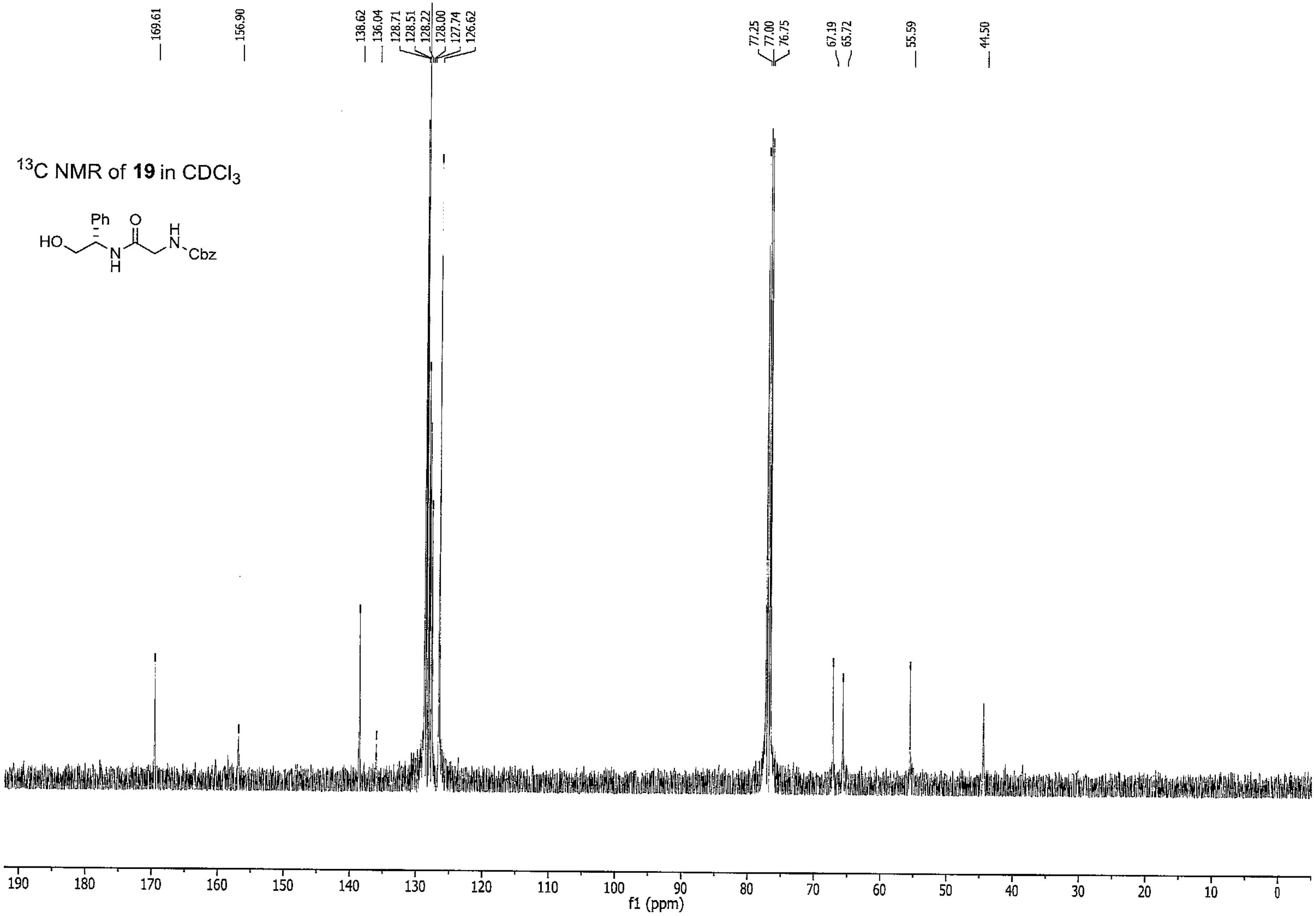

^{13}C NMR of **19** in $CDCl_3$
Ph
O
HO
N
H
N
H
Cbz
169.61
156.90
138.62
136.04
128.71
128.51
128.22
128.00
127.74
126.62
77.25
77.00
76.75
67.19
65.72
55.59
44.50
190
180
170
160
150
140
130
120
110
100
90
80
70
60
50
40
30
20
10
0
f1 (ppm)

^{1}H NMR of **20** in $CDCl_3$

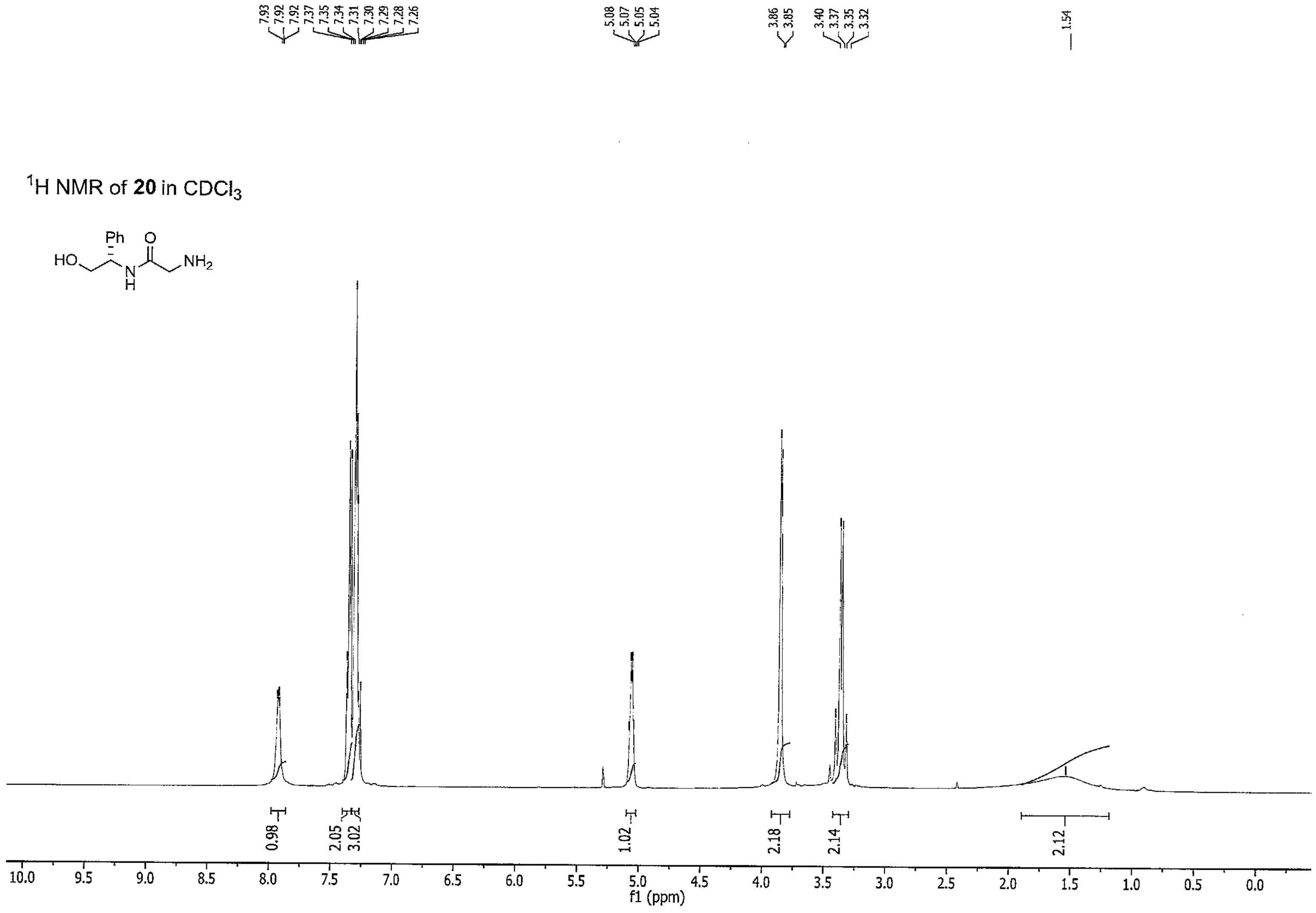

^{13}C NMR of **20** in $CDCl_3$

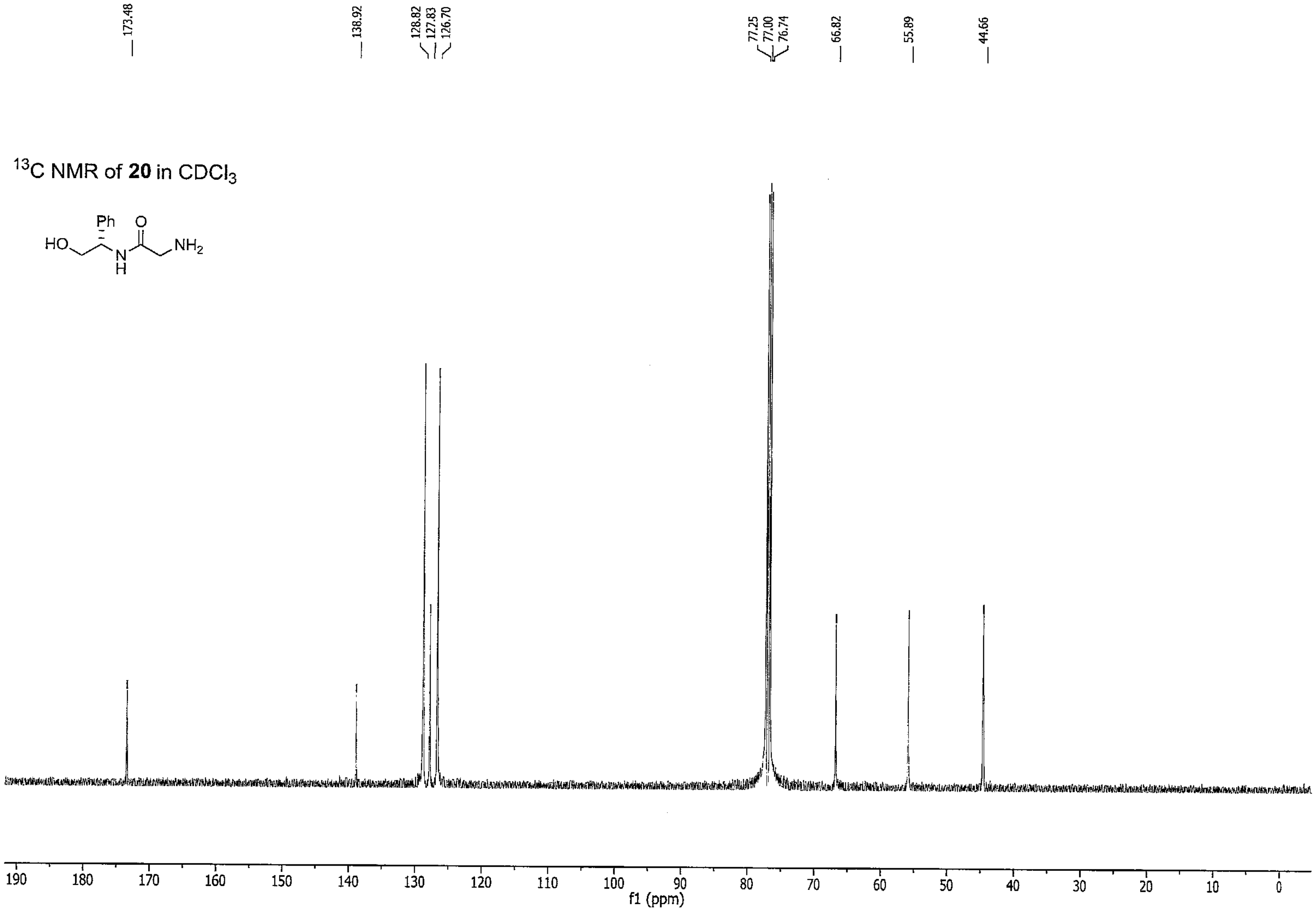

^1H NMR of **21** in dmso-d_6

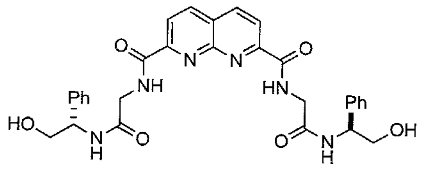

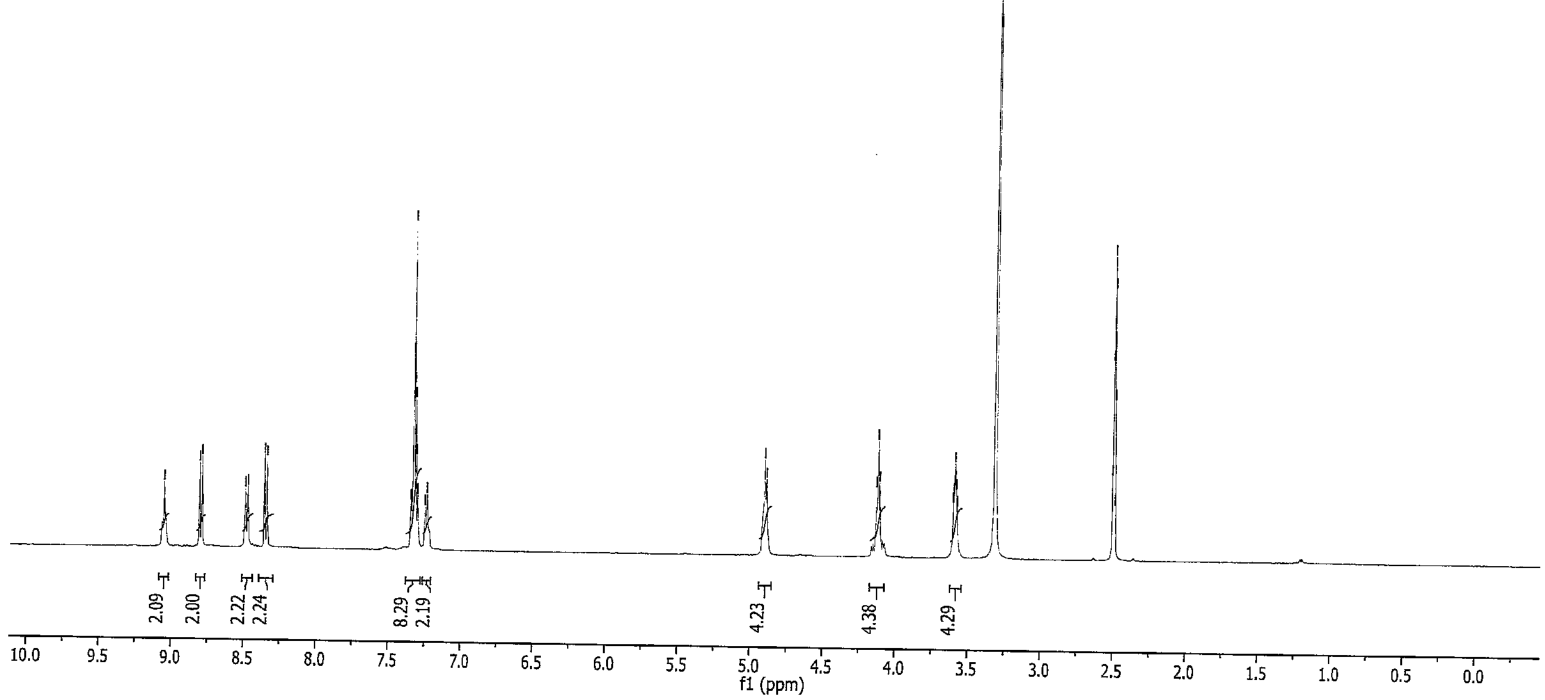

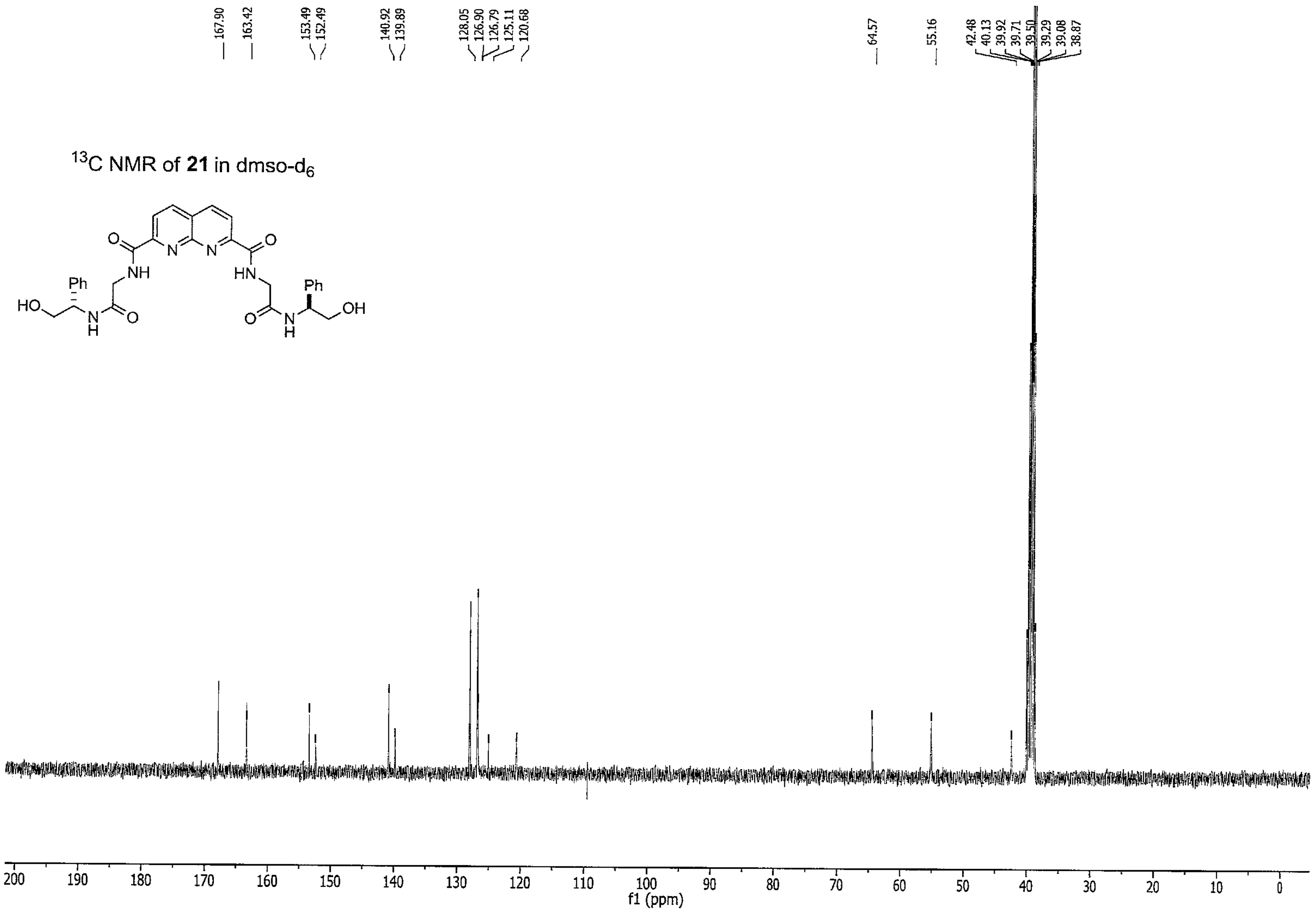

^{13}C NMR of **21** in dmso-d_6

^1H NMR of **22**-H_2 in $CDCl_3$

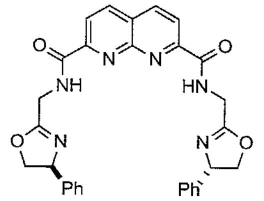

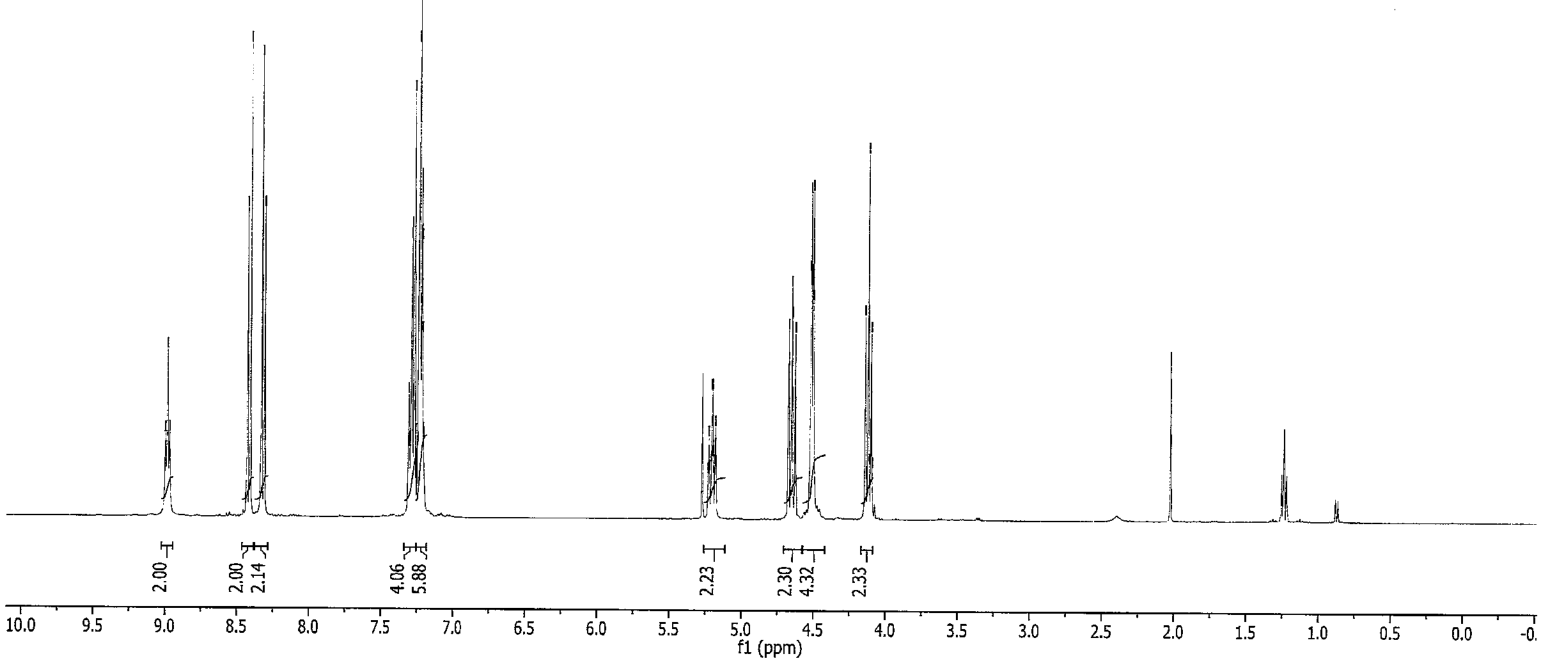

^{13}C NMR of **22**-H_2 in $CDCl_3$

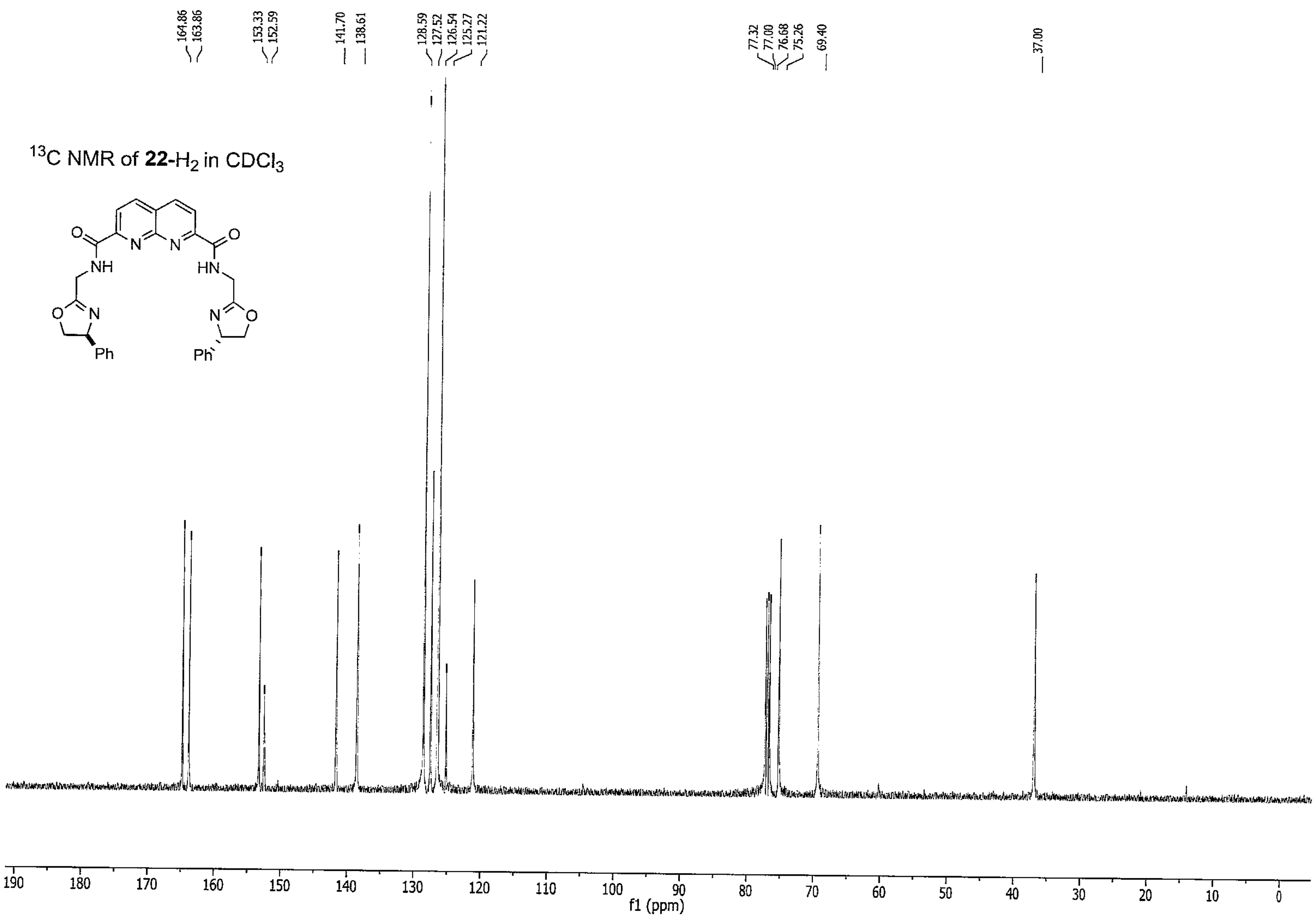

^{1}H NMR of **25**-H_3 in $CDCl_3$

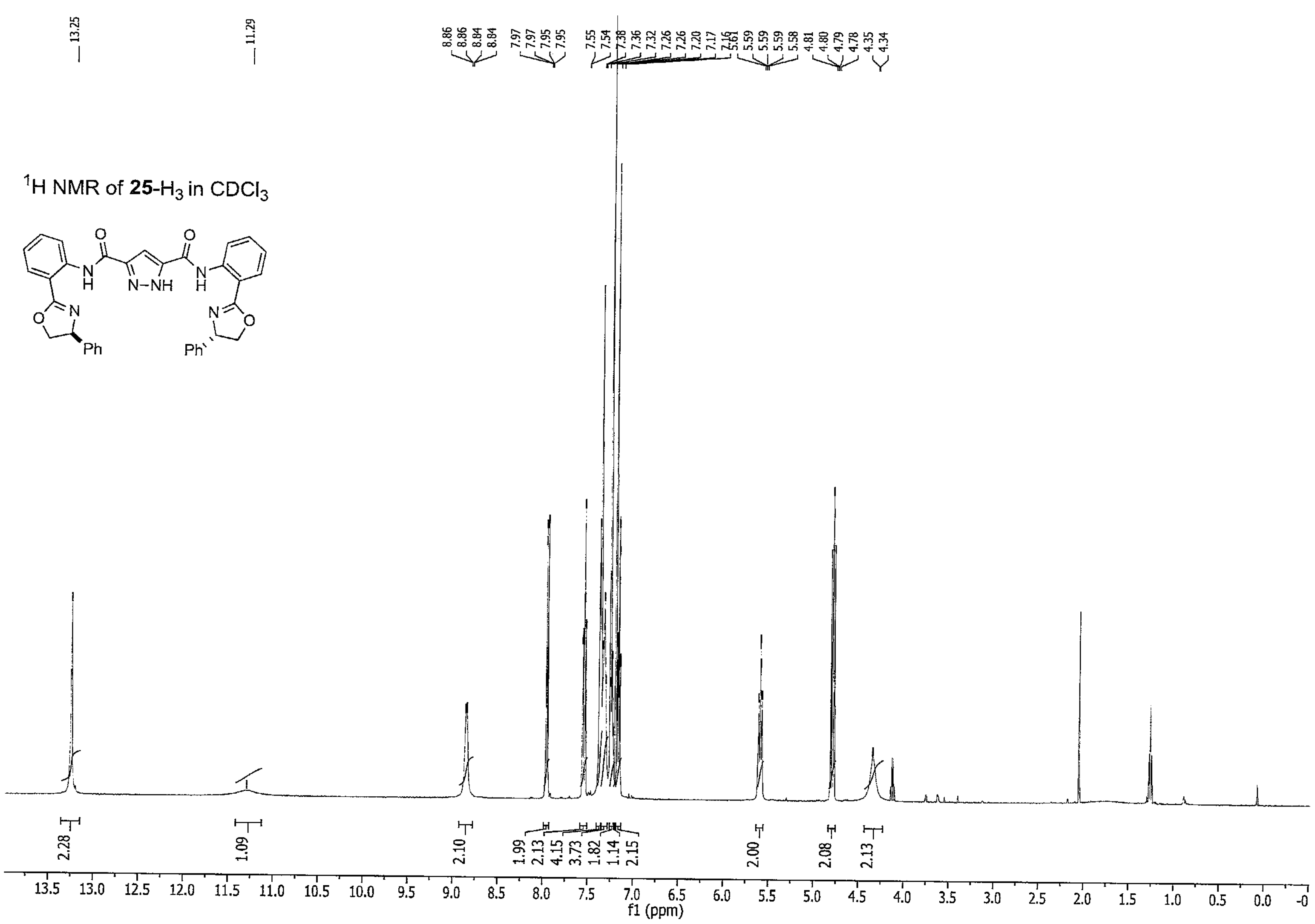

^{13}C NMR of **25**-H_3 in $CDCl_3$

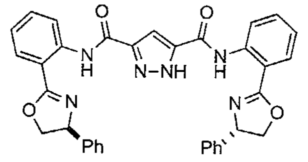

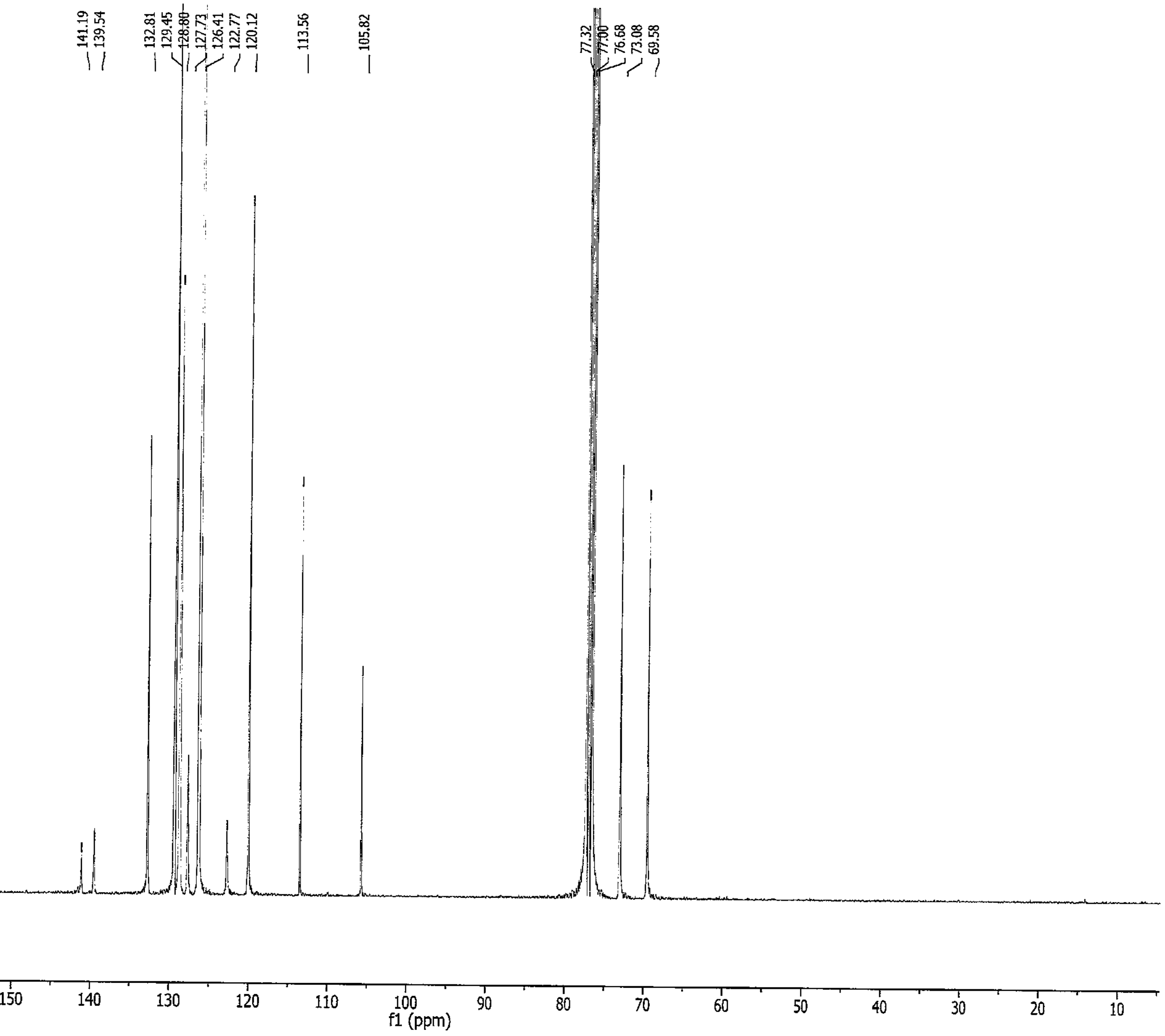

^1H NMR of **27** in $CDCl_3$

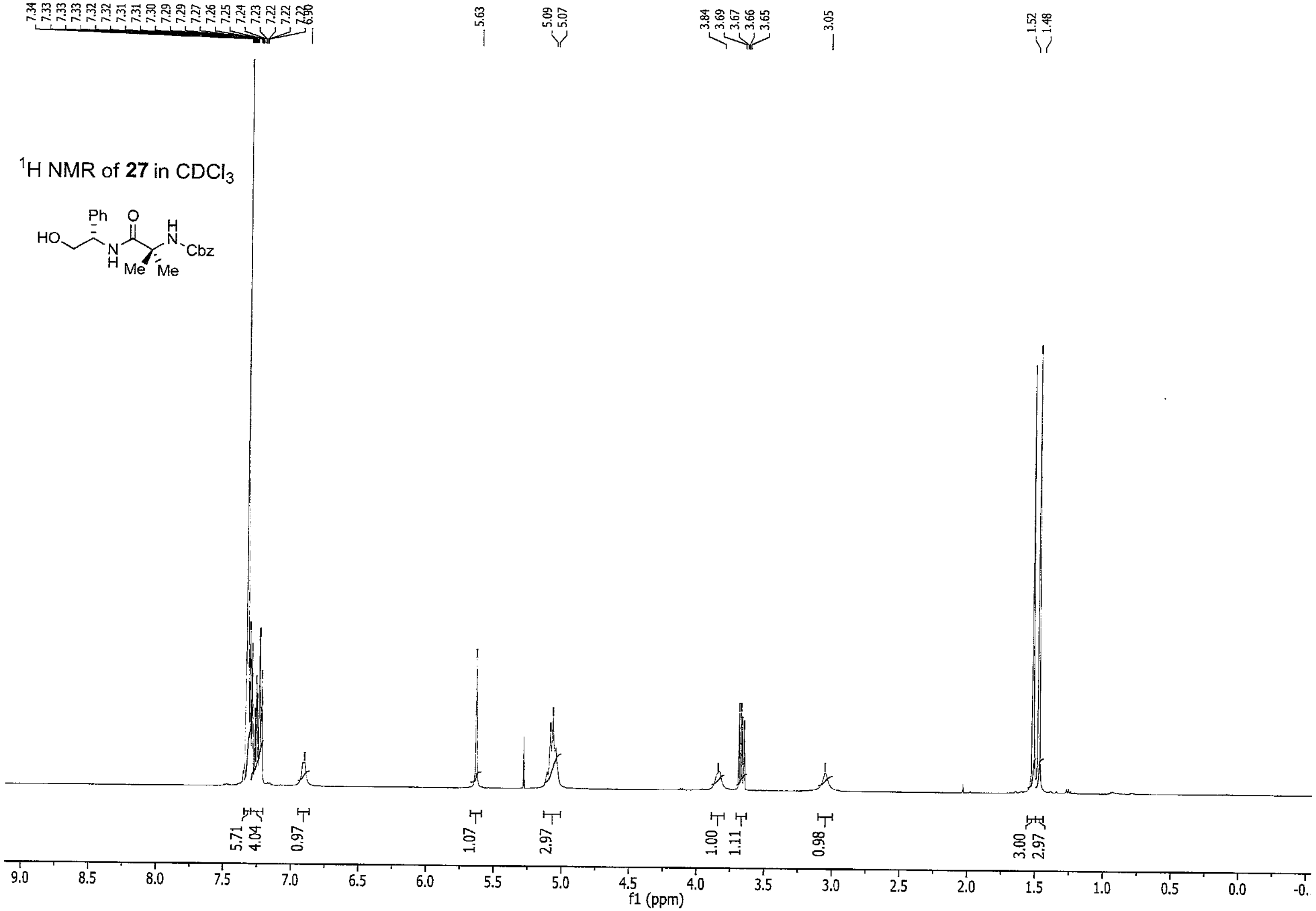

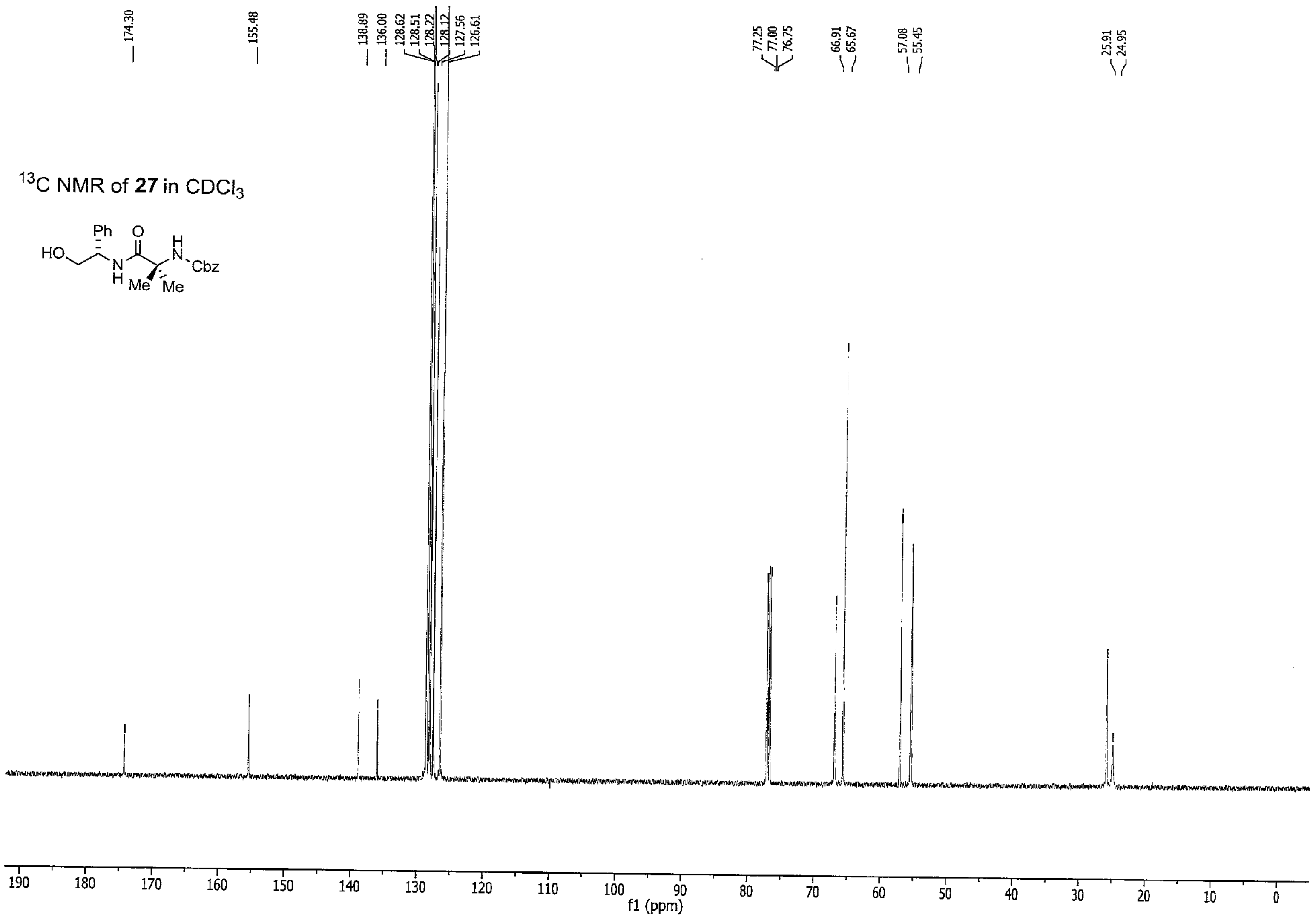
174.30
155.48
138.89
136.00
128.62
128.51
128.22
128.12
127.56
126.61
77.25
77.00
76.75
66.91
65.67
57.08
55.45
25.91
24.95
13C NMR of 27 in CDCl3
Ph
O
H
HO
N
N
Cbz
H
Me
Me
190
180
170
160
150
140
130
120
110
100
90
80
70
60
50
40
30
20
10
0
f1 (ppm)

^{1}H NMR of **29** in $CDCl_3$

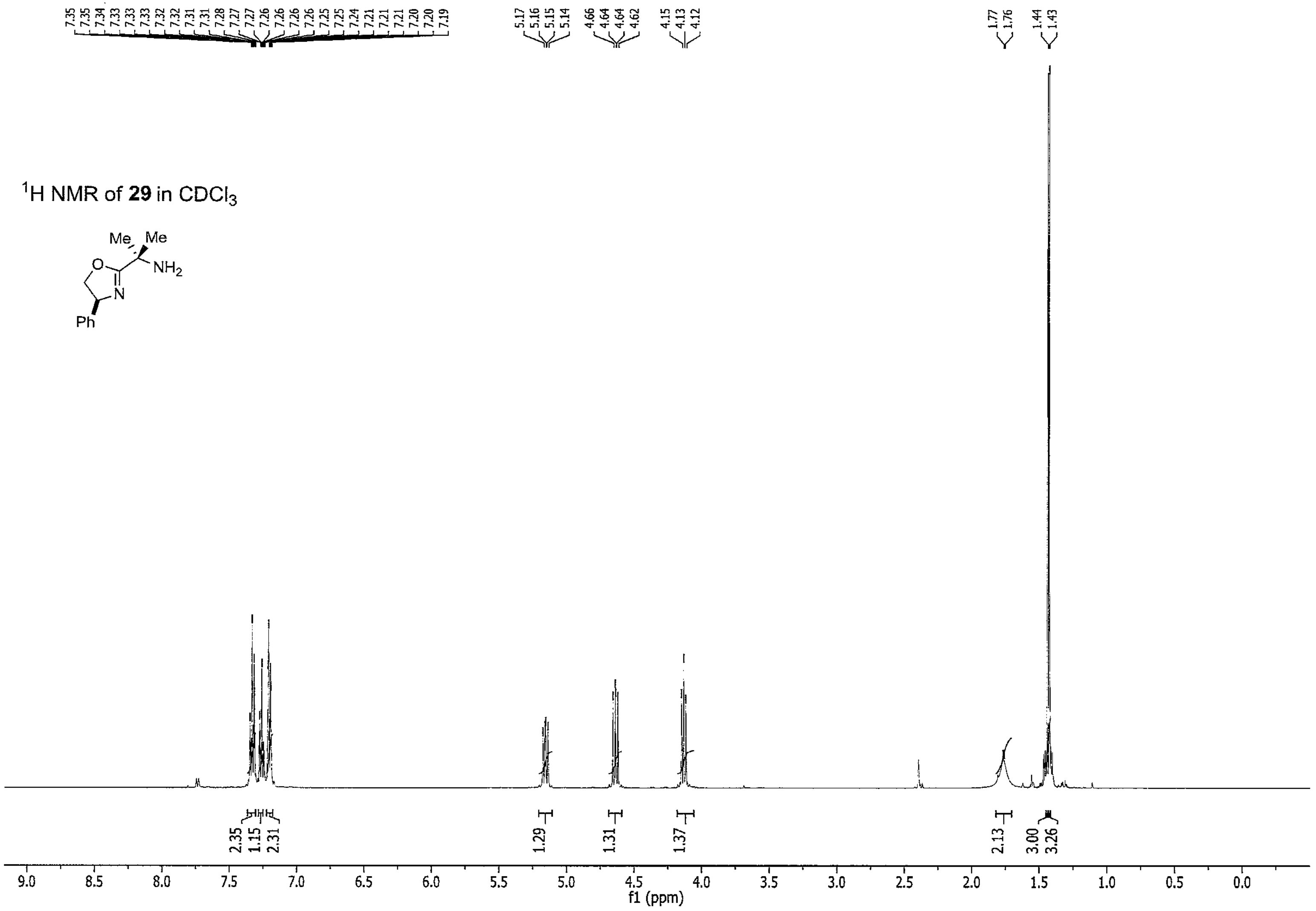

^{13}C NMR of **29** in $CDCl_3$

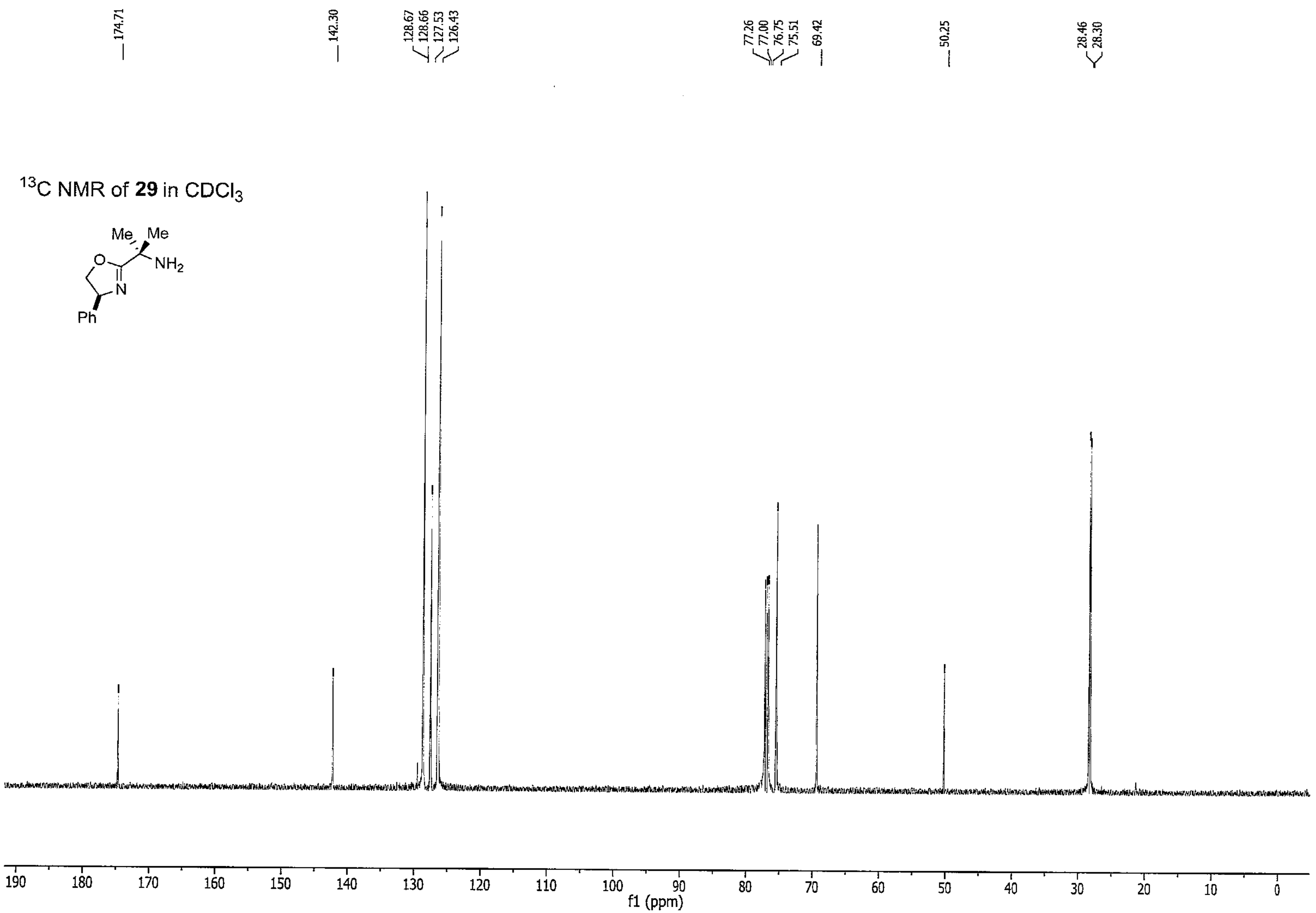

^1H NMR of **30**-H_3 in $CDCl_3$

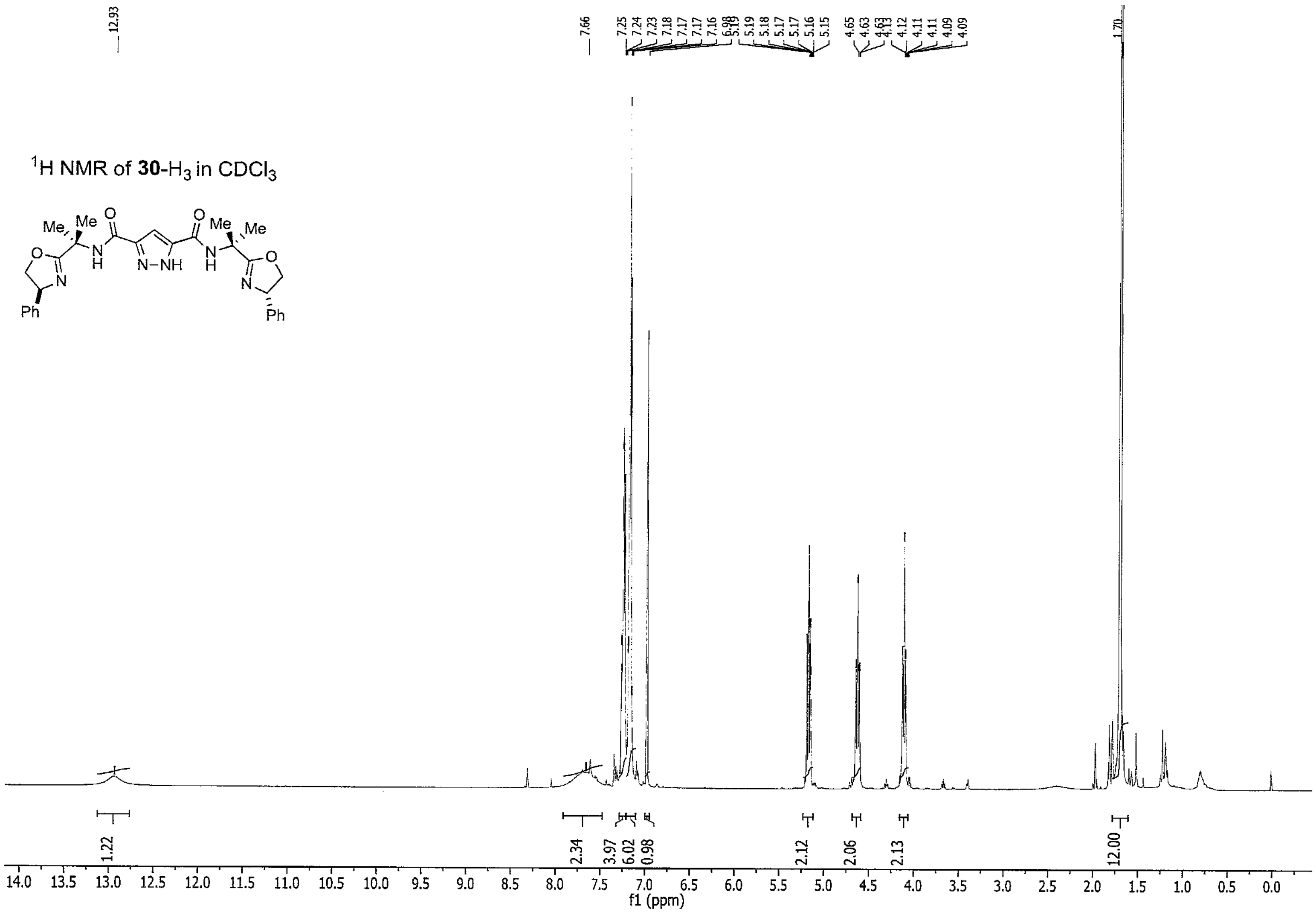

^{13}C NMR of **30**-H_3 in $CDCl_3$

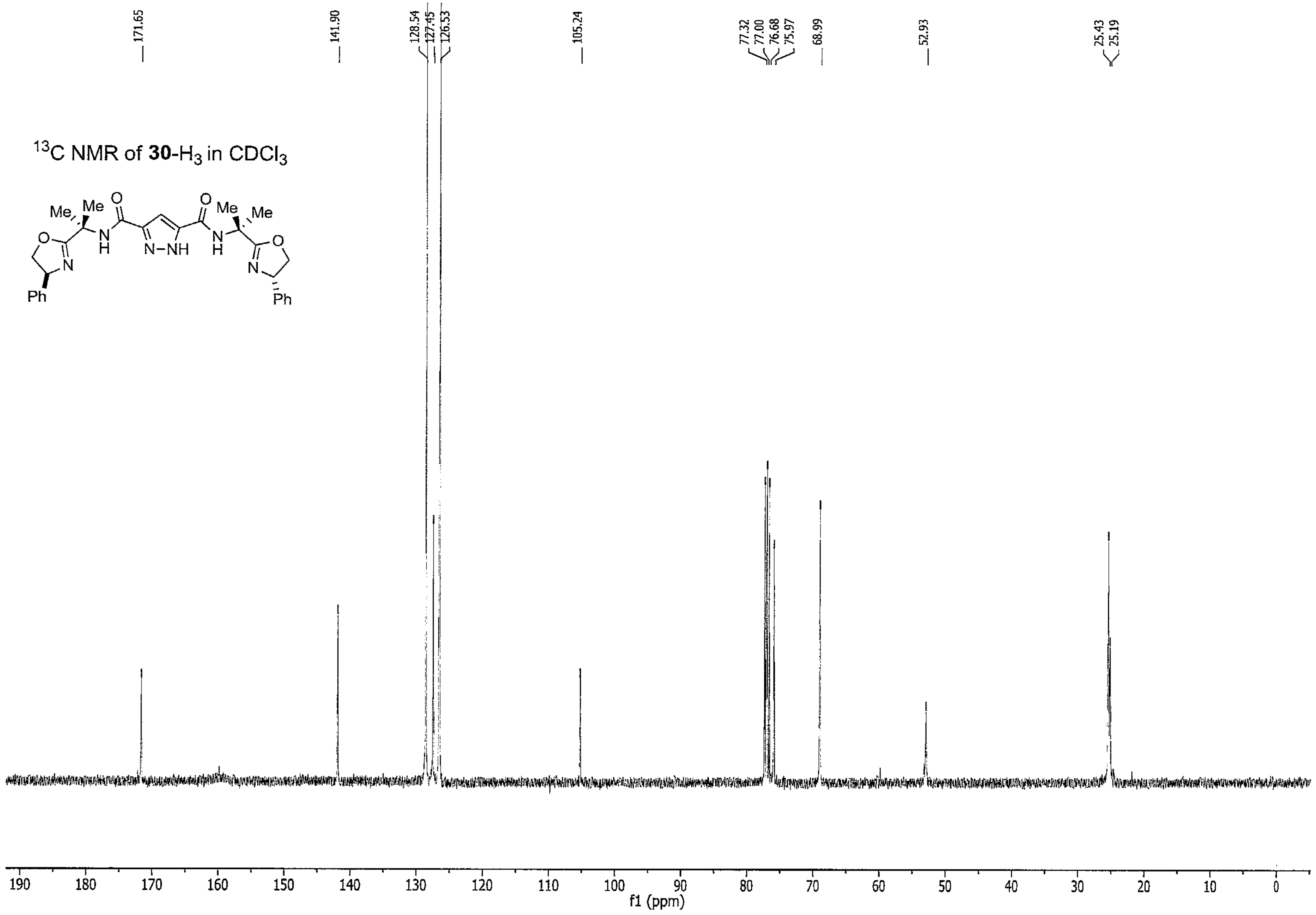

^1H NMR of **32**-H_2 in $CDCl_3$

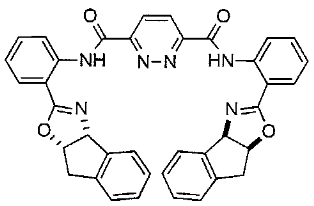

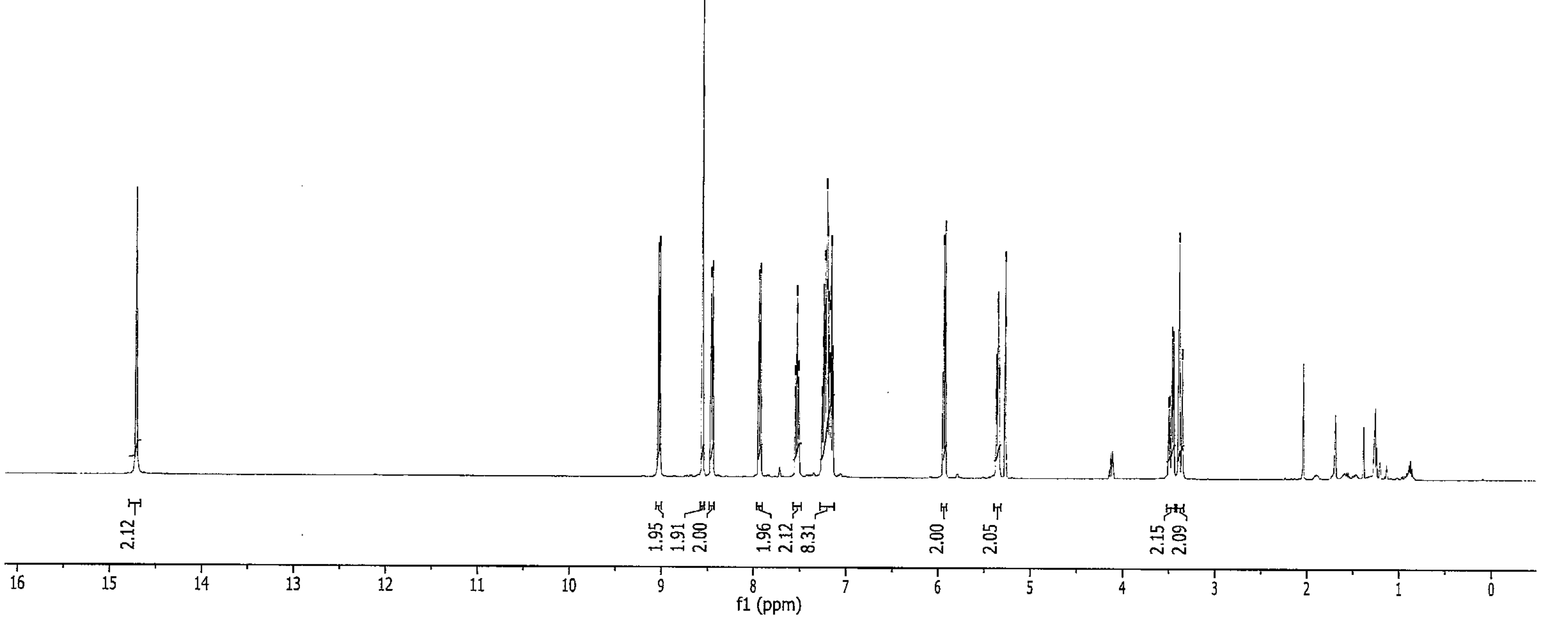

^{13}C NMR of **32**-H_2 in $CDCl_3$

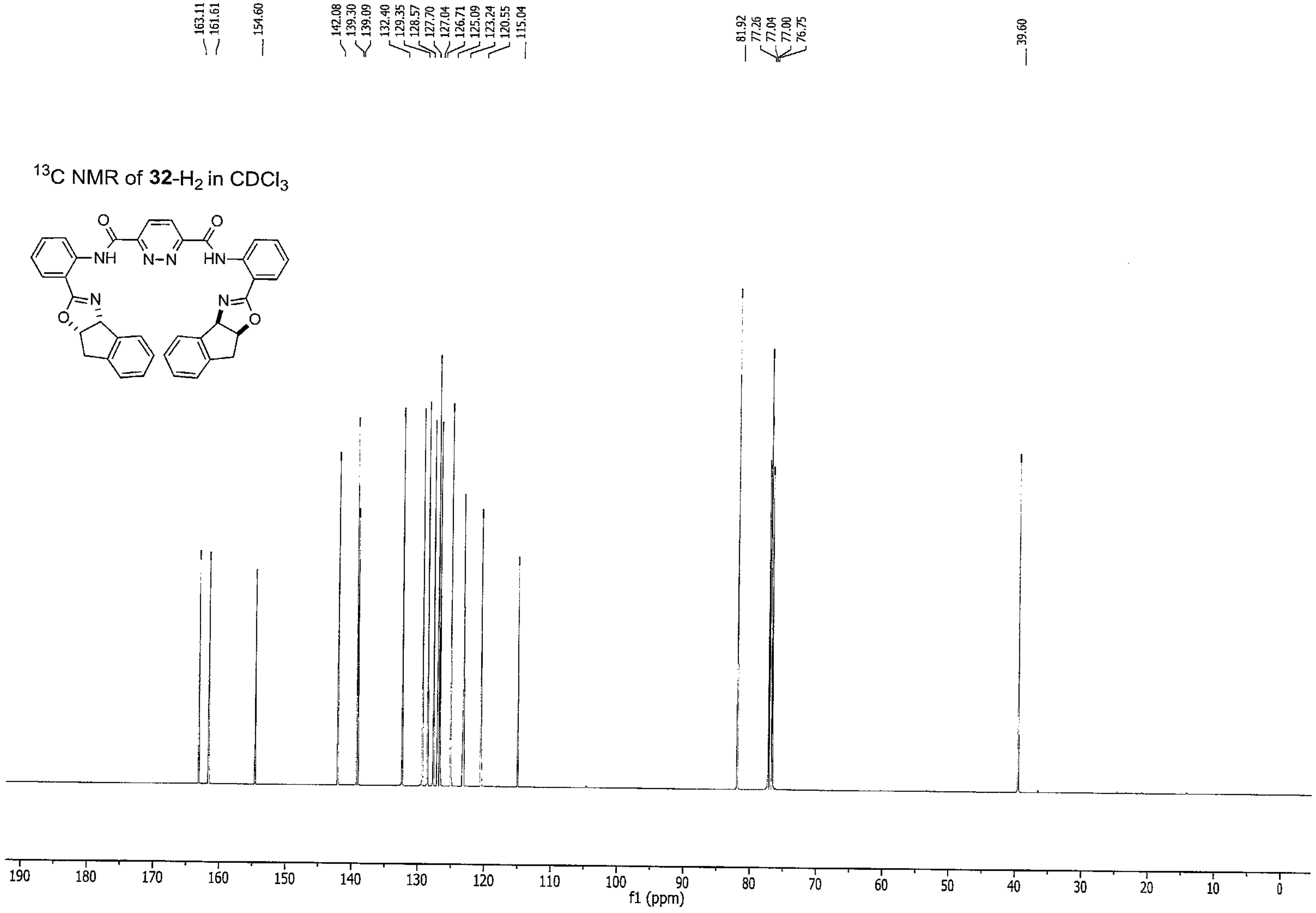

^{1}H NMR of **34**-H_3 in $CDCl_3$

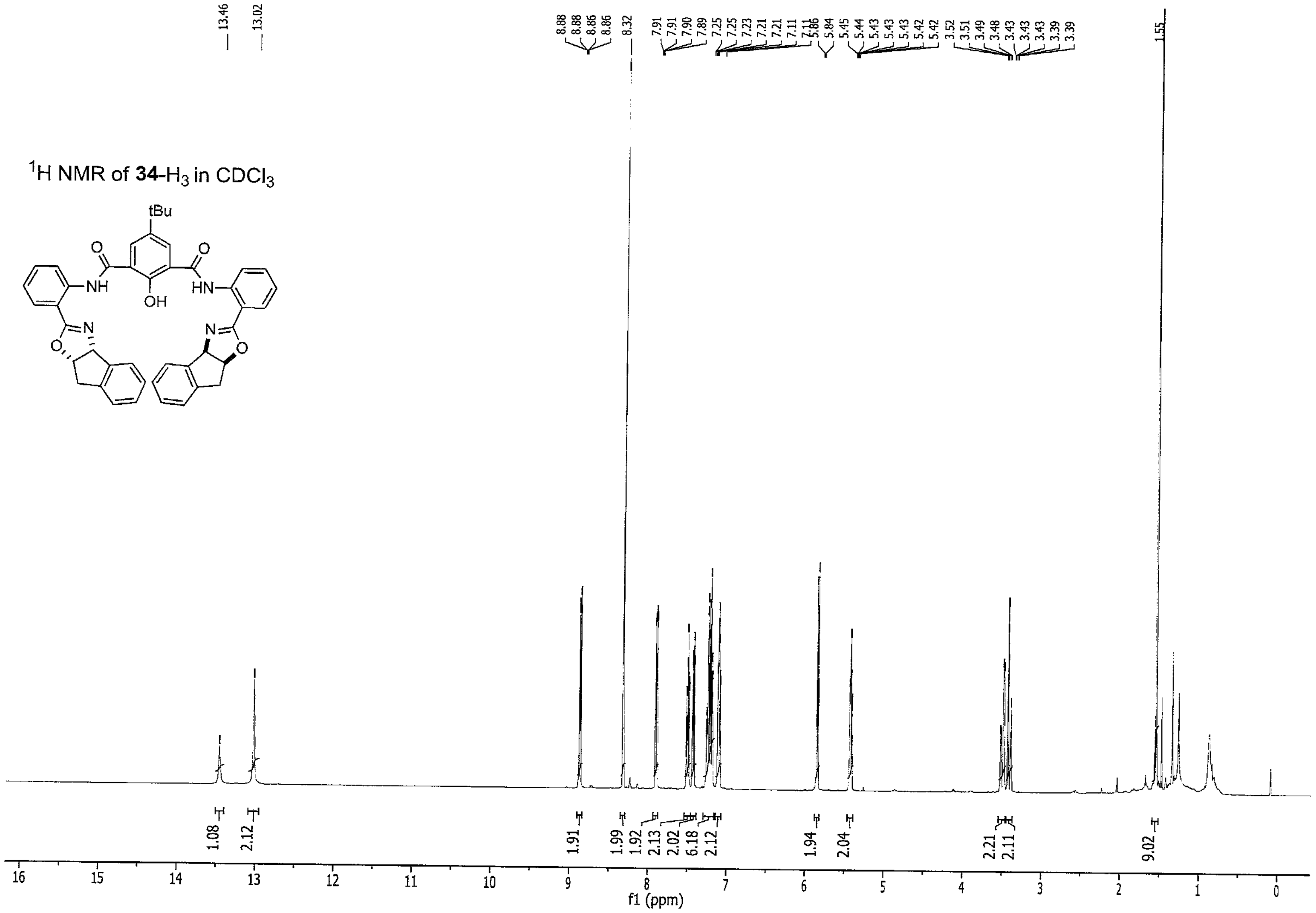

^{13}C NMR of **34**-H_3 in $CDCl_3$

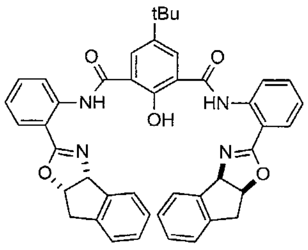

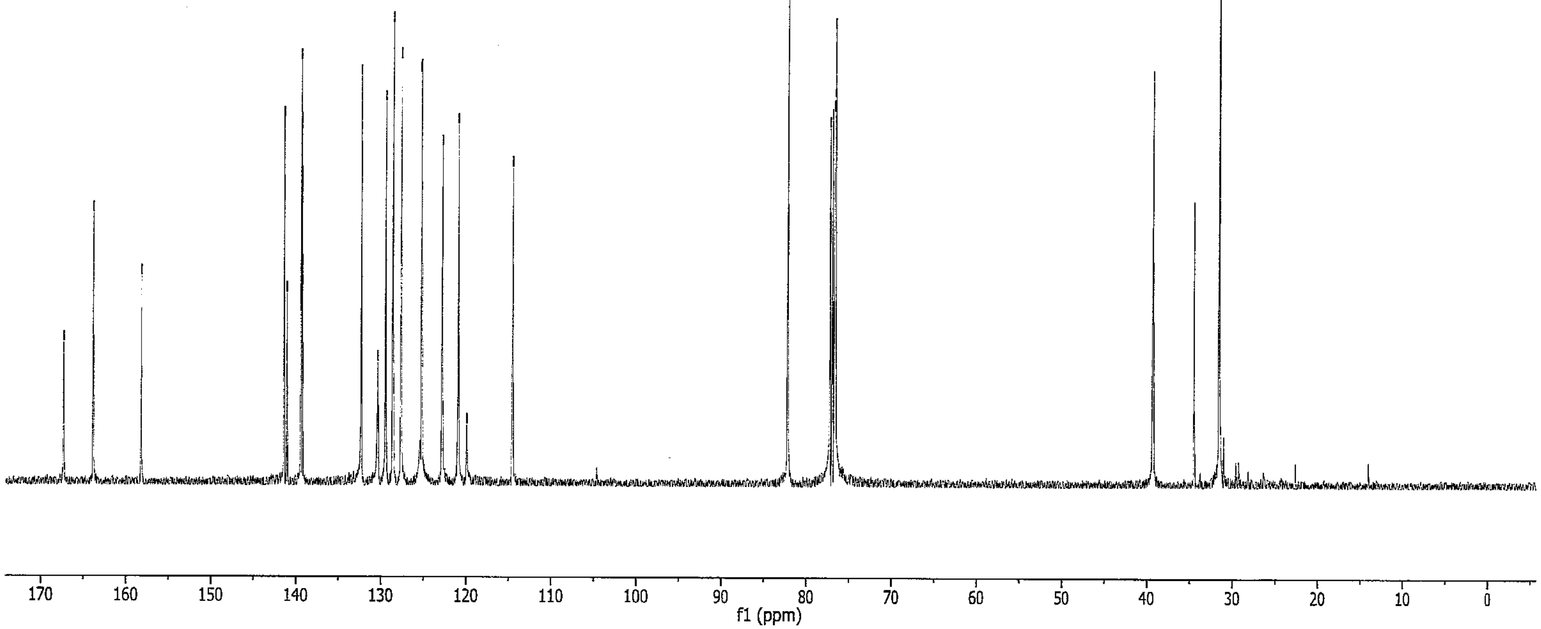

Supplement: File 1 — Experimental procedures and characterization data. [file Beilstein_J_Org_Chem-14-2002-s001.pdf]
